# Exogenous Hormones, Tumor Intrinsic Subtypes, and Breast Cancer

**DOI:** 10.1001/jamanetworkopen.2025.19236

**Published:** 2025-07-07

**Authors:** Charlotte Le Cornet, Audrey Y. Jung, Sabine Behrens, Pooja Middha, Thérèse Truong, Helena Jernström, Manjeet K. Bolla, Qin Wang, Melissa C. Southey, Laura E. Beane Freeman, Stella Koutros, Jennifer Stone, Gad Rennert, Katerina Shulman, Kristan J. Aronson, Rachel A. Murphy, Pascal Guénel, Alpa V. Patel, Clara Bodelon, Lauren R. Teras, Shamim Shahi, James V. Lacey, Laure Dossus, Rudolf Kaaks, Bernd Holleczek, Hermann Brenner, Hiltrud Brauch, Reiner Hoppe, Kamila Czene, Per Frans Leonard Hall, Arto Mannermaa, Anna H. Wu, Nadia Obi, Kyriaki Michailidou, Mihalis I. Panayiotidis, Catriona McLean, Christopher A. Haiman, Annelie Augustinsson, Wei Zheng, Xiao-Ou Shu, Charles M. Perou, Melissa A. Troester, Sarah Van Alsten, A. Heather Eliassen, Mustapha Abubakar, Peter Kraft, Thomas U. Ahearn, D. Gareth Evans, Alicja Wolk, Roger L. Milne, Douglas F. Easton, Paul D. P. Pharoah, Marjanka K. Schmidt, Montserrat García-Closas, Celine M. Vachon, Renée T. Fortner, Jenny Chang-Claude

**Affiliations:** 1 Division of Cancer Epidemiology, German Cancer Research Center, Heidelberg, Germany; 2 Division of General Internal Medicine, Department of Medicine, University of California, San Francisco; 3 Paris-Saclay University, Université de Versailles Saint-Quentin-en-Yvelines, Institut National de la Santé et de la Recherche Médicale, Gustave Roussy, Centre for Epidemiology and Population Health, Villejuif, France; 4 Division of Oncology, Department of Clinical Sciences, Lund University, Lund, Sweden; 5 Department of Public Health and Primary Care, Centre for Cancer Genetic Epidemiology, University of Cambridge, Cambridge, United Kingdom; 6 Precision Medicine, School of Clinical Sciences at Monash Health, Monash University, Clayton, Victoria, Australia; 7 Department of Clinical Pathology, University of Melbourne, Melbourne, Victoria, Australia; 8 Cancer Epidemiology Division, Cancer Council Victoria, Melbourne, Victoria, Australia; 9 Division of Cancer Epidemiology and Genetics, National Cancer Institute, National Institutes of Health, US Department of Health and Human Services, Bethesda, Maryland; 10 Genetic Epidemiology Group, School of Population and Global Health, University of Western Australia, Perth, Western Australia, Australia; 11 Centre for Epidemiology and Biostatistics, Melbourne School of Population and Global Health, University of Melbourne, Melbourne, Victoria, Australia; 12 Technion-Israel Institute of Technology–Association for Promotion of Research in Precision Medicine, Faculty of Medicine, Haifa, Israel; 13 Oncology Department, Clalit Health Services, Haifa and Western Galilee District, Israel; 14 Department of Public Health Sciences, Cancer Research Institute, Queen’s University, Kingston, Ontario, Canada; 15 School of Population and Public Health, University of British Columbia, Vancouver, British Columbia, Canada; 16 Cancer Control Research, BC Cancer Agency, Vancouver, British Columbia, Canada; 17 Department of Population Science, American Cancer Society, Atlanta, Georgia; 18 Department of Computational and Quantitative Medicine, City of Hope, Duarte, California; 19 City of Hope Comprehensive Cancer Center, City of Hope, Duarte, California; 20 Nutrition and Metabolism Branch, International Agency for Research on Cancer, Lyon, France; 21 Saarland Cancer Registry, Saarbrücken, Germany; 22 Division of Clinical Epidemiology and Aging Research, German Cancer Research Center, Heidelberg, Germany; 23 German Cancer Research Center, German Cancer Consortium, Heidelberg, Germany; 24 Dr Margarete Fischer-Bosch Institute of Clinical Pharmacology, Stuttgart, Germany; 25 iFIT (Image-Guided and Functionally Instructed Tumor Therapies) Cluster of Excellence, University of Tübingen, Tübingen, Germany; 26 German Cancer Consortium and German Cancer Research Center Partner Site Tübingen, Tübingen, Germany; 27 University of Tübingen, Tübingen, Germany; 28 Department of Medical Epidemiology and Biostatistics, Karolinska Institutet, Stockholm, Sweden; 29 Department of Oncology, Södersjukhuset, Stockholm, Sweden; 30 Translational Cancer Research Area, University of Eastern Finland, Kuopio, Finland; 31 Pathology and Forensic Medicine, Institute of Clinical Medicine, University of Eastern Finland, Kuopio, Finland; 32 Biobank of Eastern Finland, Kuopio University Hospital, Kuopio, Finland; 33 Department of Population Health and Public Health Sciences, Keck School of Medicine, University of Southern California Norris Comprehensive Cancer Center, Los Angeles; 34 Institute for Occupational and Maritime Medicine, University Medical Center Hamburg-Eppendorf, Hamburg, Germany; 35 Institute for Medical Biometry and Epidemiology, University Medical Center Hamburg-Eppendorf, Hamburg, Germany; 36 Biostatistics Unit, Cyprus Institute of Neurology and Genetics, Nicosia, Cyprus; 37 Department of Cancer Genetics, Therapeutics, and Ultrastructural Pathology, Cyprus Institute of Neurology and Genetics, Nicosia, Cyprus; 38 Now with Department of Comparative Biomedical Sciences, College of Veterinary Medicine, Mississippi State University, Starkville; 39 Anatomical Pathology Unit, Alfred Hospital, Melbourne, Victoria, Australia; 40 Department of Population and Public Health Sciences, Center for Genetic Epidemiology, Keck School of Medicine, University of Southern California, Los Angeles; 41 Division of Epidemiology, Department of Medicine, Vanderbilt Epidemiology Center, Vanderbilt-Ingram Cancer Center, Vanderbilt University School of Medicine, Nashville, Tennessee; 42 Department of Genetics, Lineberger Comprehensive Cancer Center, University of North Carolina, Chapel Hill; 43 Department of Epidemiology, Gillings School of Global Public Health, UNC Lineberger Comprehensive Cancer Center, University of North Carolina, Chapel Hill; 44 Channing Division of Network Medicine, Department of Medicine, Brigham and Women’s Hospital and Harvard Medical School, Boston, Massachusetts; 45 Department of Epidemiology, Harvard T. H. Chan School of Public Health, Boston, Massachusetts; 46 Department of Nutrition, Harvard T. H. Chan School of Public Health, Boston, Massachusetts; 47 Division of Evolution and Genomic Sciences, Faculty of Biology, Medicine, and Health, Manchester Academic Health Science Centre, School of Biological Sciences, University of Manchester, Manchester, United Kingdom; 48 North West Genomics Laboratory Hub, St Mary’s Hospital, Manchester University NHS Foundation Trust, Manchester Academic Health Science Centre, Manchester Centre for Genomic Medicine, Manchester, United Kingdom; 49 Institute of Environmental Medicine, Karolinska Institutet, Stockholm, Sweden; 50 Department of Oncology, Centre for Cancer Genetic Epidemiology, University of Cambridge, Cambridge, United Kingdom; 51 Department of Computational Biomedicine, Cedars-Sinai Medical Center, West Hollywood, California; 52 Division of Molecular Pathology, Netherlands Cancer Institute–Antoni van Leeuwenhoek Hospital, Amsterdam, the Netherlands; 53 Division of Psychosocial Research and Epidemiology, Netherlands Cancer Institute–Antoni van Leeuwenhoek Hospital, Amsterdam, the Netherlands; 54 Department of Clinical Genetics, Leiden University Medical Center, Leiden, the Netherlands; 55 Division of Genetics and Epidemiology, Institute of Cancer Research, London, United Kingdom; 56 Division of Epidemiology, Department of Quantitative Health Sciences, Mayo Clinic, Rochester, Minnesota; 57 Department of Research, Cancer Registry of Norway, Norwegian Institute of Public Health, Oslo, Norway; 58 Cancer Epidemiology Group, University Medical Center Hamburg-Eppendorf, University Cancer Center Hamburg, Hamburg, Germany

## Abstract

**Importance:**

Etiologic heterogeneity in breast carcinogenesis needs to be well characterized for targeted prevention. Associations between menopausal hormonal therapy (MHT) and oral contraceptive (OC) use and breast cancer intrinsic-like subtypes are not well understood.

**Objective:**

To examine whether exogenous hormone use is differentially associated with breast cancer subtypes and to evaluate heterogeneity by intrinsic-like subtypes.

**Design, Setting, and Participants:**

This study pooled data from 31 nested and population-based case-control studies involved in the Breast Cancer Association Consortium. The study population included individuals with breast cancer and control participants from 13 case-control studies nested in prospective cohorts (recruited between 1982 and 2011) and 18 population-based case-control studies (recruited between 1990 and 2013). Data analysis was performed in June 2024.

**Exposure:**

MHT use (estrogen-progestin therapy [EPT] or estrogen-only therapy [ET]) in postmenopausal women and OC use in premenopausal women (never, past use, or current use).

**Main Outcomes and Measures:**

Breast cancer intrinsic-like subtypes (luminal A–like, luminal B–like, luminal B–ERBB2 [formerly HER2 or HER2/neu]-like, ERBB2 enriched–like, or triple-negative) were determined by immunohistochemistry of tumor sections. Polytomous logistic regression was performed to estimate the association between exogenous hormones and risk of breast cancer by intrinsic-like subtypes. Analyses by subtypes were stratified by body mass index (BMI [calculated as weight in kilograms divided by height in meters squared]; healthy weight, 18.5-<25; overweight, 25-<30; or obesity, ≥30).

**Results:**

This study included 42 269 individuals with breast cancer (11 901 [28.2%] premenopausal and 30 368 [71.8%] postmenopausal; 23 353 [55.2%] had a known intrinsic-like subtype) and 71 072 control participants. The mean (SD) age of all participants was 57.9 (10.9) years. In postmenopausal women, associations between current MHT use (EPT or ET) and breast cancer differed by subtype. Current EPT users with healthy weight were more likely to be diagnosed with luminal A–like (odds ratio [OR], 2.51 [95% CI, 2.26-2.80]) or luminal B–ERBB2-like (OR, 1.95 [95% CI, 1.61-2.37]) subtypes. These associations were attenuated but remained for individuals with overweight (OR, 1.40 [95% CI, 1.02-1.92]) or obesity (OR, 1.68 [95% CI, 1.01-2.78]). EPT use increased the odds of being diagnosed with luminal B–like tumors solely in women with healthy weight (OR, 1.47 [95% CI, 1.17-1.86]). Current ET use was positively associated with luminal A–like disease in women with healthy weight only (OR, 1.16 [95% CI, 1.01-1.32]), showing inverse associations with higher BMI (obesity: OR, 0.65 [95% CI, 0.50-0.85]). In premenopausal women, recent OC use was associated with luminal B–ERBB2-like (OR, 1.50 [95% CI, 1.09-2.08]), ERBB2 enriched–like (OR, 2.33 [95% CI, 1.55-3.51]), and triple-negative (OR, 1.75 [95% CI, 1.33-2.29]; *P* < .04 for heterogeneity) tumors.

**Conclusions and Relevance:**

In this study, clear differences were observed in associations between current EPT use and luminal-like breast cancer subtypes and other subtypes. EPT users with healthy weight were more likely to be diagnosed with luminal-like breast cancer compared with nonusers. Subtype heterogeneity was less apparent in associations of OC and ET use. Future studies on contemporary formulations, patterns of use, and routes of administration of exogenous hormone usage are warranted.

## Key Points

**Question:**

Are menopausal hormone therapy types differentially associated with breast cancer subtypes among postmenopausal women, and is premenopausal oral contraceptive use differentially associated with breast cancer subtypes?

**Findings:**

This study pooled data from 31 nested and population-based case-control studies from the Breast Cancer Association Consortium involving 42 269 individuals with breast cancer and 71 072 control participants. Distinct associations were observed between current menopausal estrogen-progestin therapy (EPT) use and luminal-like subtypes but not for other subtypes, particularly in participants with healthy weight; less apparent subtype heterogeneity was observed for estrogen-only and oral contraceptive use.

**Meaning:**

These findings suggest there is heterogeneity in breast carcinogenesis with EPT use, which could be relevant for prevention and risk prediction.

## Teaser

This study uses pooled data from 31 case-control studies involved in the Breast Cancer Association Consortium to explore whether exogenous hormone use is differentially associated with breast cancer risk and to assess heterogeneity by intrinsic-like subtypes.

## Introduction

Menopausal hormonal therapy (MHT) is associated with increased breast cancer risk.^1,2^ The magnitude of the association decreases with time since cessation and reaches a similar level as the general population after 5 years.^1,3^ MHT use is more consistently associated with breast cancer risk in leaner women.^1,2,4,5,6,7^ Associations have been shown to vary by formulation.^2^ Increased breast cancer risk with estrogen-progestin therapy (EPT) use has been found for both estrogen receptor (ER)-positive and ER-negative tumors,^1,3,5^ but with more substantial associations for the ER-positive subtype. With estrogen-only therapy (ET), in contrast, inconsistent associations have been reported by hormone receptor status, with few studies reporting on tumor subtypes^3,8^ beyond those defined by ER or progesterone receptor (PR) status alone.^2^

Oral contraceptive (OC) use is associated with increased risk of breast cancer for up to 5 years following cessation.^9,10,11^ In contrast with MHT, associations between OC use and breast cancer risk do not appear to differ by body mass index (BMI).^9,12^ Previous studies have generally not observed heterogeneity by hormone receptor subtypes^12,13,14,15^ or by intrinsic subtypes,^8,16^ although heterogeneity for select associations has been observed. For example, in one study, associations were found for the ERBB2 (formerly HER2 or HER/neu) enriched–like and triple-negative subtypes in analyses by duration of OC use and time since last OC use.^9^

Under the hypothesis of differential risk associations according to breast cancer subtypes, we combined data from 31 nested and population-based case-control studies to investigate the association between OC and MHT use and breast cancer and to test heterogeneity primarily by intrinsic-like subtype and secondarily by ER subtype. To our knowledge, this study is the first to provide a large-scale evaluation of MHT type and breast cancer by intrinsic-like subtype stratified by BMI.

## Methods

This pooled study included individuals with breast cancer and control participants from 31 nested and population-based case-control studies involved in the Breast Cancer Association Consortium.^17^ Participants were recruited between 1982 and 2011 in cohort studies and between 1990 and 2013 in case-control studies. The study sample was obtained from 13 case-control studies nested in prospective cohorts and from 18 population-based case-control studies. All individual studies were approved by their institutional review boards or medical ethical committees. Written informed consent was obtained from all study participants. This study followed the Strengthening the Reporting of Observational Studies in Epidemiology (STROBE) reporting guideline.

### Available Data

The source of tumor marker data varied across studies and included clinical records and immunohistochemistry involving full-face tumor sections or tissue microarrays.^18^ Breast cancer subtypes were classified as described in the eMethods in Supplement 1.

Exogenous hormone use data were obtained at the reference date (ie, date of diagnosis for individuals with breast cancer and date of interview for control participants) in case-control studies and from the questionnaire closest to the date of diagnosis in cohort studies (eMethods in Supplement 1). Women were defined as postmenopausal if the last menstruation occurred more than 12 months before the reference date. When menopausal status was missing (6.4% of participants), age was used to categorize women as probably premenopausal (<54 years) or postmenopausal (≥54 years), as was done previously.^19^

### Statistical Analysis

Polytomous logistic regression was used to estimate odds ratios (ORs) and 95% CIs for associations between exogenous hormone use and breast cancer subtypes (intrinsic-like and ER status), whereas unconditional logistic regression was used for breast cancer overall. EPT use and ET use (never [reference], past, or current) were assessed in postmenopausal women, and OC use (never [reference], past, or current) was assessed in premenopausal women. Duration of use (never [reference], <5 years, or ≥5 years) and time since last use (never [reference], <5 years, 5-10 years, or ≥10 years) were also assessed. All models were adjusted for age at reference date, study, and BMI. For the analysis of EPT and ET, models were further adjusted for any other exogenous hormone use (never, past, or current). ER status was known for all individuals with breast cancer; a category for missing values was included for all covariates and for intrinsic-like subtypes.

Heterogeneity in subtype associations (eg, ER-positive and ER-negative; across intrinsic-like subtypes) was evaluated by comparing models that assume common associations across subtypes to those allowing different associations by subtype.^20^ To evaluate heterogeneity in associations by BMI, associations were stratified by BMI categories as follows: lean or healthy weight (hereinafter, healthy weight) (18.5-<25 [calculated as weight in kilograms divided by height in meters squared]), overweight (25-<30), or obesity (≥30). For women with a BMI of less than 18.5 or greater than 70 (<2% of the population), BMI was categorized as missing. Heterogeneity in estimates across BMI categories was tested by fitting interaction terms in the regression models and using a Wald test.

Associations were further assessed adjusting for alcohol consumption, first-degree family history of breast cancer, breastfeeding, age at first birth, parity, age at menarche, and age at menopause (in postmenopausal women). Additionally, analyses were performed on studies with at least 10 women in each category of MHT and OC use variables.

Sensitivity analyses evaluated heterogeneity by study design (case-control vs cohort) and by studies established before or after 2000. This time period was selected given substantial changes in ET prescribing practices following the Postmenopausal Estrogen/Progestin Interventions^21^ and Women’s Health Initiative^22^ trials (eTable 1 in Supplement 1).

Statistical analyses were performed using SAS, version 9.4 (SAS Institute). Figures were created using Wolfram Mathematica, version 12.1 (Wolfram Research). *P* < .05 (2-tailed) was considered significant. Data analysis was conducted in June 2024.

## Results

This study included 42 269 individuals with invasive cancer and known ER status (23 353 [55.2%] had a known intrinsic-like subtype) and 71 072 control patients from 31 studies participating in the Breast Cancer Association Consortium.^23,24,25,26,27,28,29,30,31,32,33,34,35,36,37,38,39,40,41,42,43,44,45,46,47,48,49,50,51,52^ The mean (SD) age of all participants was 57.9 (10.9) years, and women were predominantly of European ancestry (approximatively 84%). Key characteristics of the included studies are described in eTable 1 in Supplement 1. The distributions of risk factors and intrinsic-like subtypes according to menopausal status are presented in eTable 2 in Supplement 1.

The majority of individuals with breast cancer (30 368 of 42 269 [71.8%]) were postmenopausal. Postmenopausal women had a mean (SD) age of 62.8 (7.9) years at recruitment and a mean (SD) BMI of 25.9 (5.0). Premenopausal women (11 901 [28.2%]) had a mean (SD) age of 45.7 (6.7) years at recruitment and a mean (SD) BMI of 25.2 (5.2). At the time of data collection, among premenopausal women, more than half of individuals with breast cancer and control participants reported past OC use (61% vs 56%); 10% vs 12% reported current OC use. Among postmenopausal women, a minority of individuals with breast cancer and control participants reported past EPT use (7% vs 7%), past ET use (9% vs 8%), current EPT use (13% vs 9%), or current ET use (9% vs 10%).

### MHT and Breast Cancer Risk

A total of 56 205 postmenopausal women had information available on EPT use, and 55 842 had data on ET use. Associations between MHT and breast cancer risk are presented hereinafter.

#### Combined EPT Use and Tumor Subtypes

In postmenopausal women, associations between EPT use and breast cancer subtypes (ER status and intrinsic-like subtypes) differed by BMI (*P* = .001 for interaction). The results presented hereinafter are stratified by BMI.

**Figure 1.**
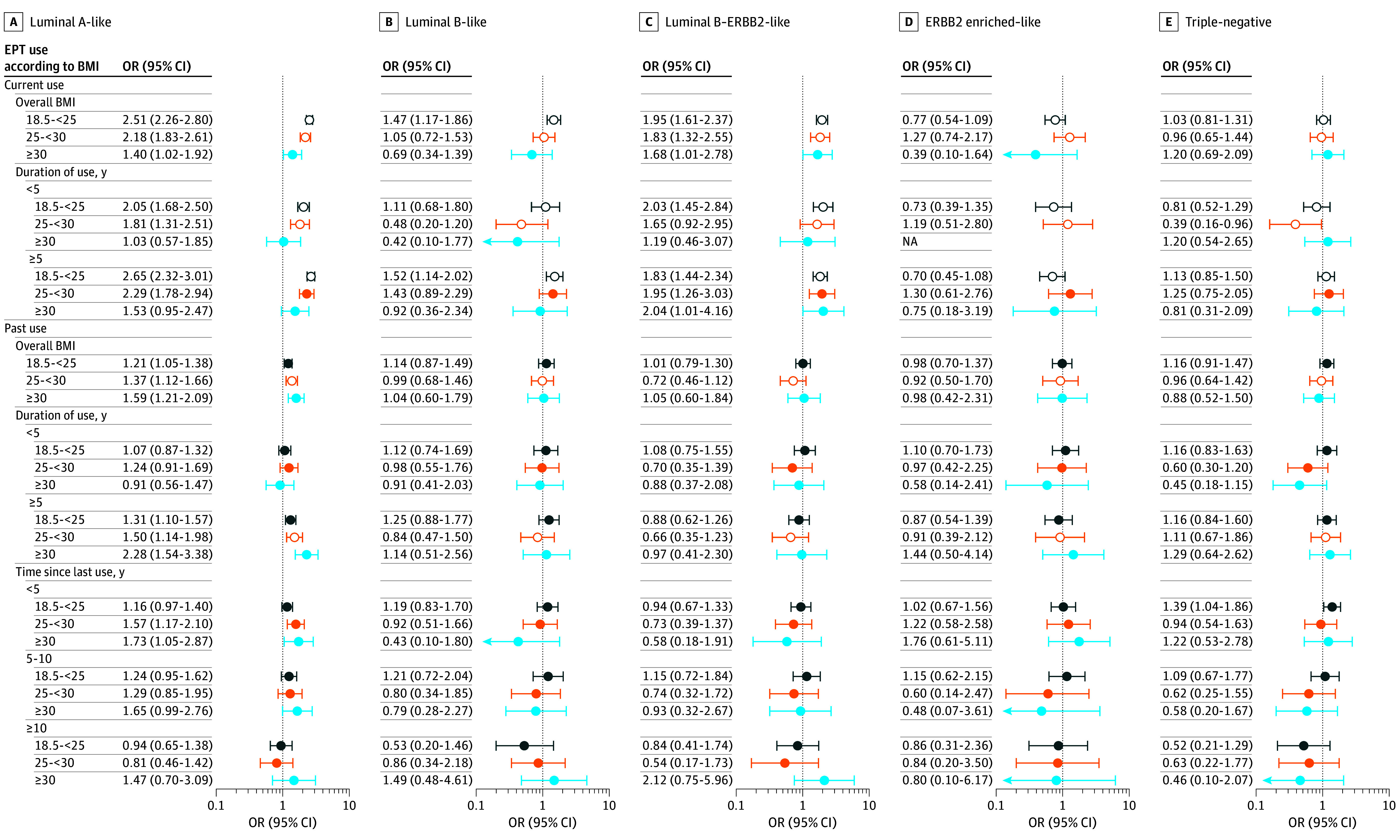
Case-Control Analyses of Associations Between Estrogen-Progestin Therapy (EPT) Use and Intrinsic-Like Subtypes According to Body Mass Index (BMI) A to E, Intrinsic-like subtypes are categorized as follows: luminal A–like (hormone receptor [HR]–positive, ERBB2 [formerly HER2 or HER2/neu]-negative, grades 1 and 2) (A), luminal B–like (HR-positive, ERBB2-negative, grade 3) (B), luminal B–ERBB2-like (HR-positive, ERBB2-positive, any grade) (C), ERBB2 enriched–like (HR-negative, ERBB2-positive, any grade) (D), and triple-negative (HR-negative, ERBB2-negative, any grade) (E). Three polytomous logistic regression models were fit (EPT use, duration of EPT use, and time since last EPT use) in association with intrinsic-like subtypes. The multivariable model was adjusted for reference age (age at diagnosis for individuals with breast cancer and age at interview for control participants), study, estrogen therapy use, and oral contraceptive use. Heterogeneity in EPT use associations between intrinsic-like subtypes was evaluated by comparing models that assume common associations across subtypes to those allowing different associations by subtype. BMI was calculated as weight in kilograms divided by height in meters squared and categorized as follows: healthy weight (18.5-<25 [dark blue markers]), overweight (25-<30 [orange markers]), or obesity (≥30 [light blue markers]). *P* < .05 was considered significant. Intrinsic-like subtype heterogeneity is denoted with open circles. Odds ratios (ORs) are presented with 95% CIs (error bars). ORs and 95% CIs could not be estimated for the association between current EPT use for less than 5 years with risk of ERBB2-enriched–like cancer due to the small number of women with obesity.

The association of current EPT use with breast cancer showed substantial heterogeneity by intrinsic-like subtype (Figure 1 and eTables 3, 4, 18, and 19 in Supplement 1). In women with healthy weight, current EPT users were more likely to be diagnosed with luminal A–like (OR, 2.51 [95% CI, 2.26-2.80]), luminal B–like (OR, 1.47 [95% CI, 1.17-1.86]), or luminal B–ERBB2–like (OR, 1.95 [95% CI, 1.61-2.37]) breast tumors, with significant heterogeneity across subtypes (*P* < .001 for heterogeneity). No association was observed for ERBB2 enriched–like and triple-negative tumors.

In women with overweight, current EPT use was positively associated with luminal A–like (OR, 2.18 [95% CI, 1.83-2.61]) and luminal B–ERBB2-like (OR, 1.83 [95% CI, 1.32-2.55]) tumors but not with other subtypes (*P* < .001 for heterogeneity). In women with obesity, associations for luminal A–like (OR, 1.40 [95% CI, 1.02-1.92]) and luminal B–ERBB2-like (OR, 1.68 [95% CI, 1.01-2.78]) tumors were seen, with smaller effect sizes and nonsignificant heterogeneity (*P* < .11 for heterogeneity).

Associations for luminal A–like and luminal B–ERBB2-like disease increased with a longer duration of EPT use among current users across BMI categories. There was no clear pattern for the other subtypes. Among past EPT users, longer duration of use (OR, 1.50 [95% CI, 1.14-1.98]) and more recent use (<5 years) (OR, 1.57 [95% CI, 1.17-2.10]) were positively associated with luminal A–like tumors in women with overweight only (*P* = .04 for heterogeneity).

#### Combined EPT Use and ER Subtypes

Associations observed for ER-positive disease were similar to those in luminal A–like and luminal B–ERBB2-like tumors. That is, consistent heterogeneity was observed with ER-negative disease for all BMI categories and in former users with obesity (eFigure 1 and eTables 4 and 19 in Supplement 1).

#### ET Use and Tumor Subtypes

In postmenopausal women, associations between ET use and breast cancer subtypes differed by BMI (*P* < .001 for interaction). Results are presented next stratified by BMI.

**Figure 2.**
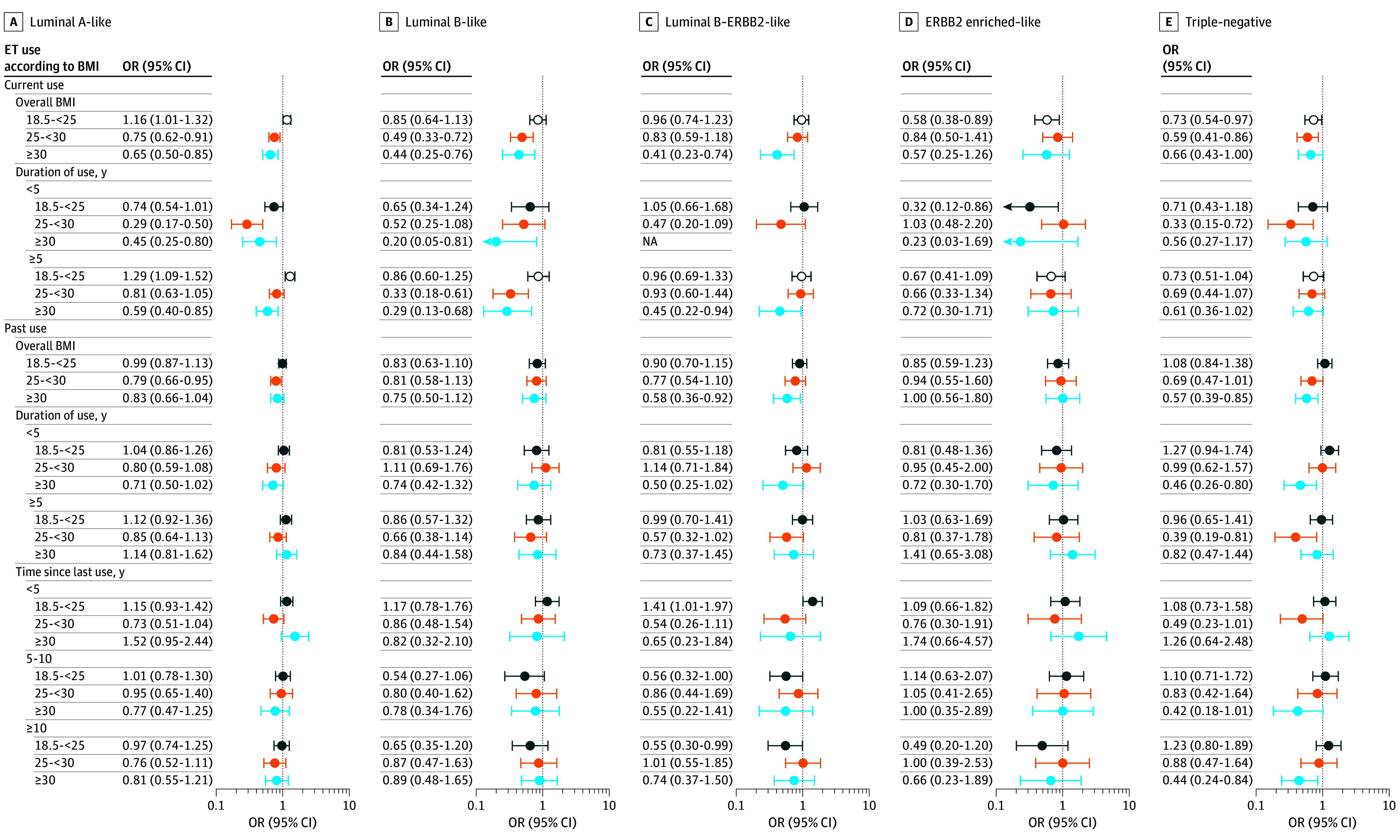
Case-Control Analyses of Associations Between Estrogen Therapy (ET) Use and Intrinsic-Like Subtypes According to Body Mass Index (BMI) A to E, Intrinsic-like subtypes are categorized as follows: luminal A–like (hormone receptor [HR]–positive, ERBB2 [formerly HER2 or HER2/neu]-negative, grades 1 and 2) (A), luminal B–like (HR-positive, ERBB2-negative, grade 3) (B), luminal B–ERBB2-like (HR-positive, ERBB2-positive, any grade) (C), ERBB2 enriched–like (HR-negative, ERBB2-positive, any grade) (D), and triple-negative (HR-negative, ERBB2-negative, any grade) (E). Three polytomous logistic regression models were fit (ET use, duration of ET use, and time since last ET use) in association with intrinsic-like subtypes. The multivariable model was adjusted for reference age (age at diagnosis for individuals with breast cancer and age at interview for control participants), study, estrogen-progestin therapy use, and oral contraceptive use. Heterogeneity in ET use associations between intrinsic-like subtypes was evaluated by comparing models that assume common associations across subtypes to those allowing different associations by subtype. BMI was calculated as weight in kilograms divided by height in meters squared and categorized as follows: healthy weight (18.5-<25 [dark blue markers]), overweight (25-<30 [orange markers]), or obesity (≥30 [light blue markers]). *P* < .05 was considered significant. Intrinsic-like subtype heterogeneity is denoted with open circles. Odds ratios (ORs) are presented with 95% CIs (error bars). Undefined estimate due to small number found in women with obesity for the association between current ET use for less than 5 years with risk of ERBB2-enriched–like cancer.

In women with healthy weight, there was significant heterogeneity in associations between current ET use and different breast cancer subtypes (*P* < .001 for heterogeneity), with a higher occurrence of luminal A–like tumors (OR, 1.16 [95% CI, 1.01-1.32]) and a lower occurrence of ERBB2-enriched–like (OR, 0.58 [95% CI, 0.38-0.89]) and triple-negative (OR, 0.73 [95% CI, 0.54-0.97]) tumors (Figure 2 and eTables 5 and 20 in Supplement 1). Longer duration of current ET use was associated with luminal A–like tumors only (OR, 1.29 [95% CI, 1.09-1.52]; *P* = .002 for heterogeneity).

Women with current ET use and overweight or obesity were less likely to be diagnosed with several subtypes (eTables 5 and 20 in Supplement 1). For example, women with current ET use and overweight were less likely to be diagnosed with luminal A–like (OR, 0.75 [95% CI, 0.62-0.91]), luminal B–like (OR, 0.49 [95% CI, 0.33-0.72]), or triple-negative (OR, 0.59 [95% CI, 0.41-0.86]) tumors. Women with current ET use and obesity were less likely to be diagnosed with luminal A–like (OR, 0.65 [95% CI, 0.50-0.85]), luminal B–like (OR, 0.44 [95% CI, 0.25-0.76]), luminal B–ERBB2-like (OR, 0.41 [95% CI, 0.23-0.74]), or triple-negative (OR, 0.66 [95% CI, 0.43-1.00]) tumors. Notably, there was no heterogeneity across subtypes for these higher BMI groups. Overall, no significant heterogeneity was observed across subtypes with former ET use.

#### ET Use and ER Subtypes

ER-positive tumors showed a similar pattern of association as luminal A–like tumors, whereas associations for ER-negative tumors were similar to triple-negative tumors (eFigure 2 and eTables 4, 5, and 19 in Supplement 1). Significant heterogeneity between ER subtypes was observed for current ET use in women with healthy weight (ER-positive vs ER-negative: OR, 1.12 [95% CI, 1.02-1.23] vs 0.83 [0.69-0.99]; *P* < .001 for heterogeneity) and also for longer duration of use (ER-positive vs ER-negative: OR, 1.17 [95% CI, 1.03-1.33] vs 0.81 [0.64-1.02]; *P* = .002 for heterogeneity).

### OC Use and Breast Cancer Risk

A total of 22 375 premenopausal women had information on OC use. Associations between OC use and breast cancer risk are presented next.

#### OC Use and Tumor Subtypes

**Figure 3.**
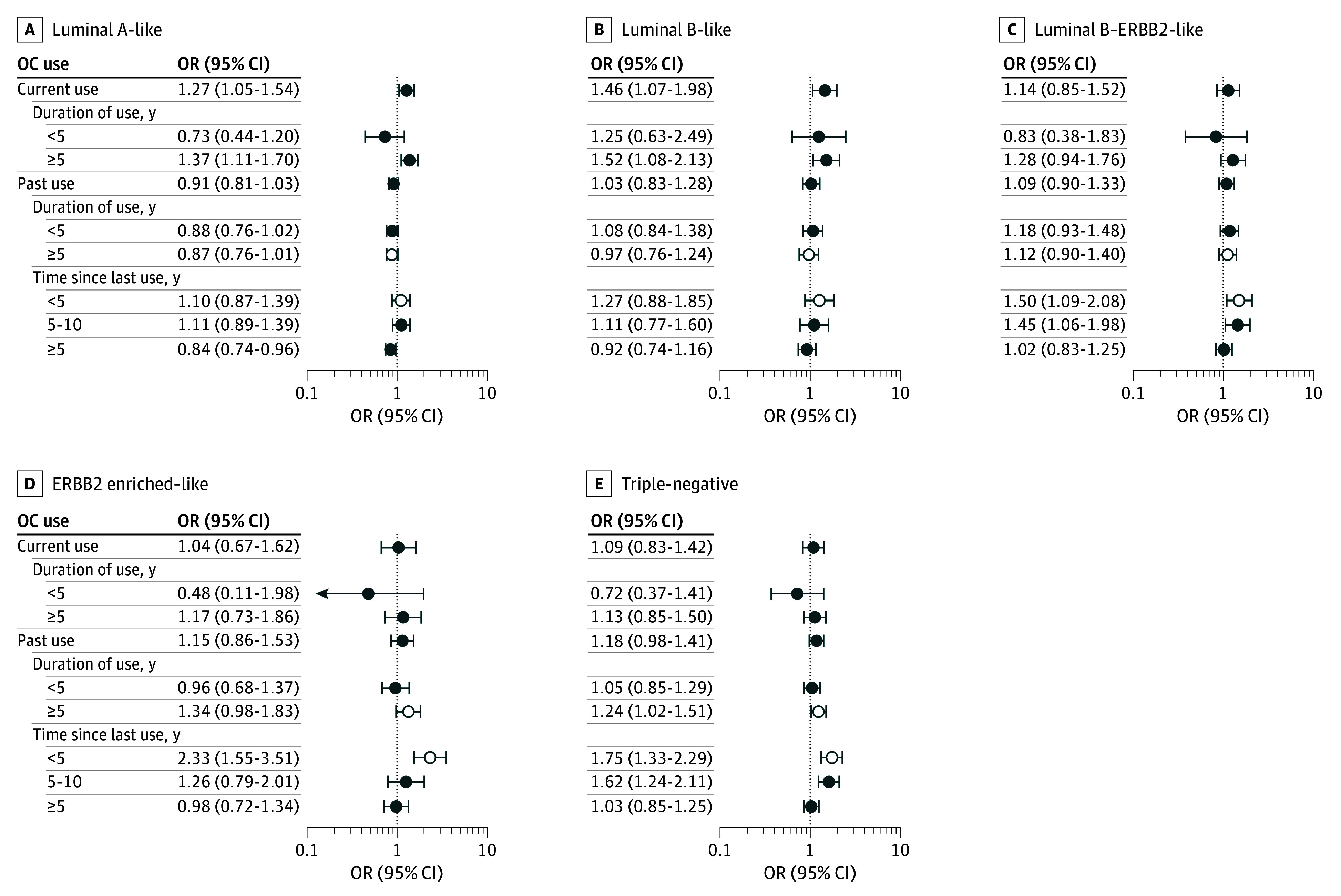
Case-Control Analyses of Associations Between Oral Contraceptive (OC) Use and Intrinsic-Like Subtypes A to E, Intrinsic-like subtypes are categorized as follows: luminal A–like (hormone receptor [HR]–positive, ERBB2 [formerly HER2 or HER2/neu]-negative, grades 1 and 2) (A), luminal B–like (HR-positive, ERBB2-negative, grade 3) (B), luminal B–ERBB2-like (HR-positive, ERBB2-positive, any grade) (C), ERBB2 enriched–like (HR-negative, ERBB2-positive, any grade) (D), and triple-negative (HR-negative, ERBB2-negative, any grade) (E). Three polytomous logistic regression models were fit: OC use, duration of OC use, and time since last OC use in association with intrinsic-like subtypes. The multivariable model was adjusted for reference age (age at diagnosis for individuals with breast cancer and age at interview for control participants), study, and body mass index. Heterogeneity in estrogen therapy use associations between intrinsic-like subtypes was evaluated by comparing models that assume common associations across subtypes to those allowing different associations by subtype. *P* < .05 was considered significant. Intrinsic-like subtype heterogeneity is denoted with open circles. Odds ratios (ORs) are presented with 95% CIs (error bars).

In premenopausal women, current OC users were more likely to be diagnosed with luminal A–like (OR, 1.27 [95% CI, 1.05-1.54]) or luminal B–like (OR, 1.46 [95% CI, 1.07-1.98]) breast cancer (Figure 3 and eTables 6 and 21 in Supplement 1). Associations between current OC use of 5 years or longer and tumor subtypes were observed for both the luminal A–like (OR, 1.37 [95% CI, 1.11-1.70]) and luminal B–like (OR, 1.52 [95% CI, 1.08-2.13]) subtypes; no association with other subtypes was observed, and the findings did not differ significantly by subtype (*P* = .14 for heterogeneity).

For past OC use, there was some indication for subtype heterogeneity. Past OC users with 5 or more years of use were more likely to be diagnosed with triple-negative disease (OR, 1.24 [95% CI, 1.02-1.51]; *P* = .01 for heterogeneity]). Compared with never users, those with recent OC use (ie, <5 years after cessation) were more likely to be diagnosed with triple-negative (OR, 1.75 [95% CI, 1.33-2.29]), ERBB2 enriched–like (OR, 2.33 [95% CI, 1.55-3.51]), or luminal-B–ERBB2-like (OR, 1.50 [95% CI, 1.09-2.08]) disease (*P* = .04 for heterogeneity). The associations between OC use and breast cancer risk were not modified by BMI.

#### OC Use and ER Subtypes

There was no clear evidence of heterogeneity in associations between OC use and breast cancer by ER status. ER-positive tumors were more likely to be diagnosed in current OC users with 5 or more years of use (OR, 1.18 [95% CI, 1.03-1.35]). Both ER subtypes were more likely to be diagnosed in past OC users compared with never users (eFigure 3 and eTables 6 and 21 in Supplement 1).

### Sensitivity Analyses

Models adjusted for additional breast cancer risk factors showed heterogeneity in associations similar to that observed in the primary analysis (eTables 7, 8, 9, 18, 20, and 21 in Supplement 1). Results from models including studies with 10 or more individuals with breast cancer reporting MHT or OC use did not differ substantially from those described earlier except for OC use, for which the association with ER-positive tumors remained for recent users only (eTables 10, 11, 12, 22, 23, and 24 in Supplement 1). In the sensitivity analysis considering study design, the overall pattern of subtype heterogeneity with current EPT use was consistent for both study types (eTables 13 and 25 in Supplement 1). However, the associations for ERBB2 enriched–like tumors differed between cohort and case-control studies, and the attenuation of associations with EPT use with increasing BMI was less apparent in cohort studies. For current ET use (eTables 14 and 26 in Supplement 1), significant heterogeneity across subtypes in women with healthy weight was observed in case-control studies but not cohort studies (*P* = .002 vs .42 for heterogeneity). No substantial differences in heterogeneity were observed by study design for associations with OC use, although the estimates tended to be higher in cohort studies than in case-control studies (eTables 15 and 27 in Supplement 1). In the sensitivity analysis considering studies set up before or after 2000 (eTables 16, 17, 28, and 29 in Supplement 1), results for subtype heterogeneity were generally consistent in both time periods.

## Discussion

This pooled analysis provides evidence for differential associations between MHT use and intrinsic breast cancer subtypes and variations in these associations by BMI. The association between current EPT use and luminal A–like and luminal B–ERBB2-like tumors was consistent across BMI groups, albeit attenuated with greater adiposity. However, the association between current EPT use and luminal B–like tumors was limited to women with healthy weight. With regard to current ET use, women with healthy weight were more likely to be diagnosed with the luminal A–like subtype, whereas women with overweight or obesity were less likely to be diagnosed with luminal A–like, luminal B–like, luminal B–ERBB2-like, or triple-negative disease subtypes. In contrast, the associations between current and past OC use appeared to be largely independent of subtype, which confirms previous findings.

The differential associations observed in this study between MHT and breast cancer risk by BMI are in line with the differences in the underlying alterations in physiologic concentrations of endogenous estrogens and progesterone following menopause.^53^ MHT is the predominant source of estrogens and progesterone among women using this treatment, with adipose tissue being the predominant source of estrogens in postmenopausal women not using MHT and low levels of progesterone produced by the adrenal cortex following cessation of progesterone production by the ovary. EPT use is largely viewed as a promotor, rather than initiator, of breast cancer, supported by both experimental^54^ and epidemiologic observations.^55^ These effects are likely predominantly receptor mediated, observed predominantly for hormone receptor–positive disease. Although ERBB2-enriched and triple-negative tumor cells have low expression of hormonal receptors, exogenous hormones may play a role in receptor-negative tumor growth through hormone-mediated and hormone-independent pathways. Estrogen may enhance angiogenesis, which promotes the development of hormone receptor–positive and hormone receptor–negative tumors.^56^ Progestins, depending on formulation, can have an agonistic or antagonistic effect when interacting with a wide set of receptors.^57^ Progestin-induced RANK-axis signaling was also shown as a potential mechanism involved in breast carcinogenesis.^58,59,60^ Although biological mechanisms are not fully elucidated for the inverse associations observed for ET, estrogen has been shown to have pro- and antiapoptotic effects on mammary tumors,^61^ with biological plausibility for differential effects by BMI given the differences in endogenous estrogen levels by BMI.

Only one prior study considered the association between MHT regimens and breast cancer intrinsic subtypes stratified by BMI.^8^ This nested case-control study found associations between EPT use and breast cancer risk to be modified by BMI, showing associations for luminal A–like and luminal B–like breast cancer risk in leaner women. No associations were observed for luminal B–ERBB2-like, ERBB2 enriched–like, and triple-negative disease overall, even when stratified by BMI. Heterogeneity across subtypes was not formally assessed, and associations with ET use were not reported stratified by BMI. The results of the prior study are in line with ours except for the association between current EPT use and luminal B–ERBB2-like disease, which we observed consistently across BMI categories.

A registry-based cohort study assessed MHT regimens and breast cancer intrinsic subtypes but did not consider potential effect modification by BMI in analyses by subtype.^2^ Current use of estradiol combined with norethisterone acetate (estradiol-NETA) was associated with a 3.5-fold increased risk of luminal A–like tumors as well as increased risk of luminal B–like, luminal B–ERBB2-like, and triple-negative tumors, with significant heterogeneity between subtypes (*P* < .001 for heterogeneity).^2^ Similarly, current estradiol intake was associated with luminal A–like, luminal B–like, and luminal B–ERBB2-like breast cancer. The authors’ observation of an association between estradiol-NETA use and triple-negative disease and between estradiol intake and risk of luminal B–like and luminal B–ERBB2-like tumors are in contrast with our findings and may be due to the study design, the specific study population, the specific types of MHT investigated, and the assessment without stratification by BMI.

We observed decreased risk of ER-positive and ER-negative breast cancer associated with current and recent ET use in women with overweight and obesity. These results differ from the meta-analysis of 58 observational studies published by the Collaborative Group^1^; however, they are in line with those from the Women’s Health Initiative trial, which observed reduced breast cancer risk with ET,^3^ and suggestive findings from other smaller trials.^21^

Relatively few studies have investigated OC use and breast cancer risk by intrinsic subtype, and results are mixed. In the Nurses’ Health Study II, current OC use for more than 5 years was associated with an increased risk of ER-positive/PR-positive/ERBB2-negative breast cancer,^9^ comparable to the associations we found for luminal A–like and luminal B–like tumors. Higher risks of triple-negative breast cancer and ERBB2 enriched–like tumors among former OC users (for >5 years) and those who ceased use within the last 10 years and significant heterogeneity by disease subtype (*P* = .02) are in line with our findings in women with healthy weight. A 2023 meta-analysis similarly found higher risk of triple-negative breast cancer with ever OC use.^62^ However, an earlier study pooling data from 9 prospective studies could not confirm any association between OC use and breast cancer intrinsic subtypes.^16^ Previous studies have also not shown differences in associations with OC use by ER subtypes,^9,16^ consistent with our findings.

### Strengths and Limitations

To our knowledge, this study is the largest to date on associations between exogenous hormone use and breast cancer intrinsic subtypes modified by BMI. There is growing interest in subtype risk prediction for cancer risk reduction medication and future therapeutic options. Our findings can inform how such models can be adapted to provide subtype-specific estimates.

This study has limitations. The studies included predominantly women of European ancestry (approximately 84%), which limits the generalizability of the findings to other racial and ethnic groups.^63^ About 20% of the tumor subtype categorization was based on the immunohistochemistry of ER and PR, which may be inaccurate due to varying thresholds for positivity and interpretation criteria.^64^ Our study is also limited by residual confounding and potential misclassification due to self-reported exposure information, as well as by timing of exogenous hormone use in relation to breast cancer diagnosis. For cohort studies, data on hormone use could be from baseline questionnaire completion, which may be years prior to diagnosis or, if available, from follow-up the closest to the individual’s breast cancer diagnosis; in population-based case-control studies, information was collected at or shortly after cancer diagnosis. Formulations and dosages of MHTs and OCs might also have differed not only across study or country but also over time. Differences in risk associations with EPT when stratified by study design might also partly reflect the different formulations of progestogen used in different countries^65^; a higher proportion of the cohort studies were from the US, whereas case-control studies were predominantly European. Nevertheless, in the analysis stratified by study design, heterogeneity between subtypes remained similar. The associations found between ERBB2 enriched–like and triple-negative subtypes with former OC use, but not with current OC use, could be explained by misclassification or by the fact that OC formulations have changed over time and their effects on breast cancer development differ.^9^ Our results are in line with those found with formulations using second-generation progestins; however, the lack of information on OC formulations hampered further confirmation.

## Conclusions

In this large-scale pooled study, clear differences were observed in associations between current EPT use and luminal-like breast cancer subtypes compared with other subtypes, particularly for women with healthy weight. Less apparent subtype heterogeneity was observed in associations between ET use and OC use. These results provide further evidence of etiologic heterogeneity in breast carcinogenesis. Future studies on contemporary formulations, patterns of use, and routes of administration of exogenous hormone usage are warranted.

## Notes

Supplement 1. **eMethods.** Breast Cancer Risk Factors **eTable 1.** Description of Studies Included in the Analysis **eTable 2.** Baseline Characteristics of Women According to Pre- and Postmenopausal Status **eTable 3.** Odds Ratios and 95% CIs of Associations Between Estrogen-Progestin Therapy (EPT) Use and Breast Cancer Intrinsic-Like Subtypes, in Postmenopausal Women, Stratified by Body Mass Index (BMI) **eTable 4.** Odds Ratios and 95% CIs of Associations Between Menopausal Hormonal Therapy (MHT) Use and Breast Cancer Subtypes, in Postmenopausal Women Overall **eTable 5.** Odds Ratios and 95% CIs of Associations Between Estrogen-Only (ET) Use and Breast Cancer Intrinsic-Like Subtypes, in Postmenopausal Women, Stratified by Body Mass Index (BMI) **eTable 6.** Odds Ratios and 95% CIs of Associations Between Oral Contraceptive Use and Breast Cancer Subtypes, in Premenopausal Women Overall **eTable 7.** Fully Adjusted Odds Ratios and 95% CIs of Associations Between Estrogen-Progestin Therapy (EPT) Use and Breast Cancer Intrinsic-Like Subtypes, in Postmenopausal Women, Stratified by Body Mass Index (BMI) **eTable 8.** Fully Adjusted Odds Ratios and 95% CIs of Associations Between Estrogen-Only Therapy (ET) Use and Breast Cancer Intrinsic-Like Subtypes, in Postmenopausal Women, Stratified by Body Mass Index (BMI) **eTable 9.** Fully Adjusted Odds Ratios and 95% CIs of Associations Between Oral Contraceptive Use and Breast Cancer Subtypes, in Premenopausal Women Overall **eTable 10.** Odds Ratios and 95% CIs of Associations Between Estrogen-Progestin Therapy (EPT) Use and Breast Cancer Intrinsic-Like Subtypes, in Postmenopausal Women, Stratified by Body Mass Index (BMI) in a Restricted Set of Studies With at Least 10 Cases Reporting EPT Use **eTable 11.** Odds Ratios and 95% CIs of Associations Between Estrogen-Only Therapy (ET) Use and Breast Cancer Intrinsic-Like Subtypes, in Postmenopausal Women, Stratified by Body Mass Index (BMI) in a Restricted Set of Studies With at Least 10 Cases Reporting ET Use **eTable 12.** Odds Ratios and 95% CIs of Associations Between Oral Contraceptive (OC) Use and Breast Cancer Subtypes, in Premenopausal Women Overall in a Restricted Set of Studies With at Least 10 Cases Reporting OC Use **eTable 13.** Associations Between Estrogen-Progestin Therapy (EPT) Use and Breast Cancer Intrinsic-Like Subtypes, in Postmenopausal Women, Stratified by Body Mass Index (BMI) and Study Design **eTable 14.** Associations Between Estrogen-Only Therapy (ET) Use and Breast Cancer Intrinsic-Like Subtypes, in Postmenopausal Women, Stratified by Body Mass Index (BMI) and Study Design **eTable 15.** Associations Between Oral Contraceptive (OC) Use and Breast Cancer Intrinsic-Like Subtypes, in Premenopausal Women Overall, Stratified by Study Design **eTable 16.** Heterogeneity Between Studies Set Up Before and After 2000 for the Associations Between Estrogen-Progestin Therapy (EPT) Use and Breast Cancer Intrinsic-Like Subtypes, in Postmenopausal Women, Stratified by Body Mass Index (BMI) **eTable 17.** Heterogeneity Between Studies Set Up Before and After 2000 for the Associations Between Estrogen-Only Therapy (ET) Use and Breast Cancer Intrinsic-Like Subtypes, in Postmenopausal Women, Stratified by Body Mass Index (BMI) **eTable 18.** Number of Women Included in the Associations Between Estrogen-Progestin Therapy (EPT) Use and Breast Cancer Intrinsic-Like Subtypes, in Postmenopausal Women, Stratified by Body Mass Index (BMI) (Corresponds to Results in eTables 3 and 7) **eTable 19.** Number of Women Included in the Associations Between Menopausal Hormonal Therapy (MHT) Use and Breast Cancer Subtypes, in Postmenopausal Women Overall (Corresponds to Results in eTable 4) **eTable 20.** Number of Women Included in the Associations Between Estrogen-Only Therapy (ET) Use and Breast Cancer Intrinsic-Like Subtypes, in Postmenopausal Women, Stratified by Body Mass Index (BMI) (Corresponds to Results in eTables 5 and 8) **eTable 21.** Number of Women Included in the Associations Between Oral Contraceptive Use and Breast Cancer Subtypes, in Premenopausal Women Overall (Corresponds to Results in eTables 6 and 9) **eTable 22.** Number of Women Included in the Associations Between Estrogen-Progestin Therapy (EPT) Use and Breast Cancer Intrinsic-Like Subtypes, in Postmenopausal Women, Stratified by Body Mass Index (BMI) in a Restricted Set of Studies With at Least 10 Cases Reporting EPT Use (Corresponds to Results in eTable 10) **eTable 23.** Number of Women Included in the Associations Between Estrogen-Only Therapy (ET) and Breast Cancer Intrinsic-Like Subtypes, in Postmenopausal Women, Stratified by Body Mass Index (BMI) in a Restricted Set of Studies With at Least 10 Cases Reporting ET Use (Corresponds to Results in eTable 11) **eTable 24.** Number of Women Included in the Associations Between Oral Contraceptive (OC) Use and Breast Cancer Subtypes, in Premenopausal Women Overall in a Restricted Set of Studies With At Least 10 Cases Reporting OC Use (Corresponds to Results in eTable 12) **eTable 25.** Number of Women Included in the Associations Between Estrogen-Progestin Therapy (EPT) Use and Breast Cancer Intrinsic-Like Subtypes, in Postmenopausal Women, Stratified by Body Mass Index (BMI) and Study Design (Corresponds to Results in eTable 13) **eTable 26.** Number of Women Included in the Associations Between Estrogen-Only Therapy (ET) Use and Breast Cancer Intrinsic-Like Subtypes, in Postmenopausal Women, Stratified by Body Mass Index (BMI) and Study Design (Corresponds to Results in eTable 14) **eTable 27.** Number of Women Included in the Associations Between Oral Contraceptive Use and Breast Cancer Intrinsic-Like Subtypes, In Premenopausal Women Overall Stratified by Study Design (Corresponds to Results in eTable 15) **eTable 28.** Number of Women Included in the Analysis on the Heterogeneity Between Studies Set Up Before and After 2000 for the Associations Between Estrogen-Progestin Therapy (EPT) Use and Breast Cancer Intrinsic-Like Subtypes, in Postmenopausal Women, Stratified by Body Mass Index (BMI) (Corresponds to Results in eTable 16) **eTable 29.** Number of Women Included in the Analysis on the Heterogeneity Between Studies Set Up Before and After 2000 for the Associations Between Estrogen-Only Therapy (ET) Use and Breast Cancer Intrinsic-Like Subtypes, In Postmenopausal Women, Stratified by Body Mass Index (BMI) (Corresponds to Results in eTable 17) **eFigure 1.** Odds Ratios and 95% CIs for Case-Control Analyses of Associations Between Estrogen-Progestin Therapy (EPT) Use and Breast Cancer Overall as well as Estrogen Receptor (ER)-Positive and ER-Negative Breast Cancer According to Body Mass Index (BMI) **eFigure 2.** Odds Ratios and 95% CIs for Case-Control Analyses of Associations Between Estrogen Therapy (ET) Use and Breast Cancer Overall as well as Estrogen Receptor (ER)-Positive and ER-Negative Breast Cancer According to Body Mass Index (BMI) **eFigure 3.** Odds Ratios and 95% CIs for Case-Control Analyses of Associations Between Oral Contraceptive (OC) Use and Breast Cancer Overall as well as Estrogen Receptor (ER)-Positive and ER-Negative Breast Cancer
eReferences
**eAppendix 1.** BCAC Funders **eAppendix 2.** BCAC Additional Contributions

Supplement 2.
Data Sharing Statement
