# Supplementary material for: Exogenous Hormones, Tumor Intrinsic Subtypes, and Breast Cancer
Source: JAMA Netw Open. 2025 Jul 7;8(7):e2519236. doi: 10.1001/jamanetworkopen.2025.19236 (PMC12235495; doi:10.1001/jamanetworkopen.2025.19236)
Supplement: Supplement 1. — eMethods. Breast Cancer Risk Factors eTable 1. Description of Studies Included in the Analysis eTable 2. Baseline Characteristics of Women According to Pre- and Postmenopausal Status eTable 3. Odds Ratios and 95% CIs of Associations Between Estrogen-Progestin Therapy (EPT) Use and Breast Cancer Intrinsic-Like Subtypes, in Postmenopausal Women, Stratified by Body Mass Index (BMI) eTable 4. Odds Ratios and 95% CIs of Associations Between Menopausal Hormonal Therapy (MHT) Use and Breast Cancer Subtypes, in Postmenopausal Women Overall eTable 5. Odds Ratios and 95% CIs of Associations Between Estrogen-Only (ET) Use and Breast Cancer Intrinsic-Like Subtypes, in Postmenopausal Women, Stratified by Body Mass Index (BMI) eTable 6. Odds Ratios and 95% CIs of Associations Between Oral Contraceptive Use and Breast Cancer Subtypes, in Premenopausal Women Overall eTable 7. Fully Adjusted Odds Ratios and 95% CIs of Associations Between Estrogen-Progestin Therapy (EPT) Use and Breast Cancer Intrinsic-Like Subtypes, in Postmenopausal Women, Stratified by Body Mass Index (BMI) eTable 8. Fully Adjusted Odds Ratios and 95% CIs of Associations Between Estrogen-Only Therapy (ET) Use and Breast Cancer Intrinsic-Like Subtypes, in Postmenopausal Women, Stratified by Body Mass Index (BMI) eTable 9. Fully Adjusted Odds Ratios and 95% CIs of Associations Between Oral Contraceptive Use and Breast Cancer Subtypes, in Premenopausal Women Overall eTable 10. Odds Ratios and 95% CIs of Associations Between Estrogen-Progestin Therapy (EPT) Use and Breast Cancer Intrinsic-Like Subtypes, in Postmenopausal Women, Stratified by Body Mass Index (BMI) in a Restricted Set of Studies With at Least 10 Cases Reporting EPT Use eTable 11. Odds Ratios and 95% CIs of Associations Between Estrogen-Only Therapy (ET) Use and Breast Cancer Intrinsic-Like Subtypes, in Postmenopausal Women, Stratified by Body Mass Index (BMI) in a Restricted Set of Studies With at Least 10 Cases Reporting ET Use eTable 12. Odds Ratios [file jamanetwopen-e2519236-s001.pdf]

Supplemental Online Content

Le Cornet C, Jung A, Behrens S, et al. Exogenous hormones, tumor intrinsic subtypes, and breast cancer. *JAMA Netw Open*. 2025;8(7):e2519236.
doi:10.1001/jamanetworkopen.2025.19236

eMethods. Breast Cancer Risk Factors

eTable 1. Description of Studies Included in the Analysis

eTable 2. Baseline Characteristics of Women According to Pre- and Postmenopausal Status

eTable 3. Odds Ratios and 95% CIs of Associations Between Estrogen-Progestin Therapy (EPT) Use and Breast Cancer Intrinsic-Like Subtypes, in Postmenopausal Women, Stratified by Body Mass Index (BMI)

eTable 4. Odds Ratios and 95% CIs of Associations Between Menopausal Hormonal Therapy (MHT) Use and Breast Cancer Subtypes, in Postmenopausal Women Overall

eTable 5. Odds Ratios and 95% CIs of Associations Between Estrogen-Only (ET) Use and Breast Cancer Intrinsic-Like Subtypes, in Postmenopausal Women, Stratified by Body Mass Index (BMI)

eTable 6. Odds Ratios and 95% CIs of Associations Between Oral Contraceptive Use and Breast Cancer Subtypes, in Premenopausal Women Overall

eTable 7. Fully Adjusted Odds Ratios and 95% CIs of Associations Between Estrogen-Progestin Therapy (EPT) Use and Breast Cancer Intrinsic-Like Subtypes, in Postmenopausal Women, Stratified by Body Mass Index (BMI)

eTable 8. Fully Adjusted Odds Ratios and 95% CIs of Associations Between Estrogen-Only Therapy (ET) Use and Breast Cancer Intrinsic-Like Subtypes, in Postmenopausal Women, Stratified by Body Mass Index (BMI)

eTable 9. Fully Adjusted Odds Ratios and 95% CIs of Associations Between Oral Contraceptive Use and Breast Cancer Subtypes, in Premenopausal Women Overall

eTable 10. Odds Ratios and 95% CIs of Associations Between Estrogen-Progestin Therapy (EPT) Use and Breast Cancer Intrinsic-Like Subtypes, in Postmenopausal Women, Stratified by Body Mass Index (BMI) in a Restricted Set of Studies With at Least 10 Cases Reporting EPT Use

eTable 11. Odds Ratios and 95% CIs of Associations Between Estrogen-Only Therapy (ET) Use and Breast Cancer Intrinsic-Like Subtypes, in Postmenopausal Women, Stratified by Body Mass Index (BMI) in a Restricted Set of Studies With at Least 10 Cases Reporting ET Use

eTable 12. Odds Ratios and 95% CIs of Associations Between Oral Contraceptive (OC) Use and Breast Cancer Subtypes, in Premenopausal Women Overall in a Restricted Set of Studies With at Least 10 Cases Reporting OC Use

© 2025 Le Cornet C et al. *JAMA Network Open*.

eTable 13. Associations Between Estrogen-Progestin Therapy (EPT) Use and Breast Cancer Intrinsic-Like Subtypes, in Postmenopausal Women, Stratified by Body Mass Index (BMI) and Study Design

eTable 14. Associations Between Estrogen-Only Therapy (ET) Use and Breast Cancer Intrinsic-Like Subtypes, in Postmenopausal Women, Stratified by Body Mass Index (BMI) and Study Design

eTable 15. Associations Between Oral Contraceptive (OC) Use and Breast Cancer Intrinsic-Like Subtypes, in Premenopausal Women Overall, Stratified by Study Design

eTable 16. Heterogeneity Between Studies Set Up Before and After 2000 for the Associations Between Estrogen-Progestin Therapy (EPT) Use and Breast Cancer Intrinsic-Like Subtypes, in Postmenopausal Women, Stratified by Body Mass Index (BMI)

eTable 17. Heterogeneity Between Studies Set Up Before and After 2000 for the Associations Between Estrogen-Only Therapy (ET) Use and Breast Cancer Intrinsic-Like Subtypes, in Postmenopausal Women, Stratified by Body Mass Index (BMI)

eTable 18. Number of Women Included in the Associations Between Estrogen-Progestin Therapy (EPT) Use and Breast Cancer Intrinsic-Like Subtypes, in Postmenopausal Women, Stratified by Body Mass Index (BMI) (Corresponds to Results in eTables 3 and 7)

eTable 19. Number of Women Included in the Associations Between Menopausal Hormonal Therapy (MHT) Use and Breast Cancer Subtypes, in Postmenopausal Women Overall (Corresponds to Results in eTable 4)

eTable 20. Number of Women Included in the Associations Between Estrogen-Only Therapy (ET) Use and Breast Cancer Intrinsic-Like Subtypes, in Postmenopausal Women, Stratified by Body Mass Index (BMI) (Corresponds to Results in eTables 5 and 8)

eTable 21. Number of Women Included in the Associations Between Oral Contraceptive Use and Breast Cancer Subtypes, in Premenopausal Women Overall (Corresponds to Results in eTables 6 and 9)

eTable 22. Number of Women Included in the Associations Between Estrogen-Progestin Therapy (EPT) Use and Breast Cancer Intrinsic-Like Subtypes, in Postmenopausal Women, Stratified by Body Mass Index (BMI) in a Restricted Set of Studies With at Least 10 Cases Reporting EPT Use (Corresponds to Results in eTable 10)

eTable 23. Number of Women Included in the Associations Between Estrogen-Only Therapy (ET) and Breast Cancer Intrinsic-Like Subtypes, in Postmenopausal Women, Stratified by Body Mass Index (BMI) in a Restricted Set of Studies With at Least 10 Cases Reporting ET Use (Corresponds to Results in eTable 11)

eTable 24. Number of Women Included in the Associations Between Oral Contraceptive (OC) Use and Breast Cancer Subtypes, in Premenopausal Women Overall in a Restricted Set of Studies With At Least 10 Cases Reporting OC Use (Corresponds to Results in eTable 12)

eTable 25. Number of Women Included in the Associations Between Estrogen-Progestin Therapy (EPT) Use and Breast Cancer Intrinsic-Like Subtypes, in Postmenopausal Women, Stratified by Body Mass Index (BMI) and Study Design (Corresponds to Results in eTable 13)

© 2025 Le Cornet C et al. *JAMA Network Open*.

eTable 26. Number of Women Included in the Associations Between Estrogen-Only Therapy (ET) Use and Breast Cancer Intrinsic-Like Subtypes, in Postmenopausal Women, Stratified by Body Mass Index (BMI) and Study Design (Corresponds to Results in eTable 14)

eTable 27. Number of Women Included in the Associations Between Oral Contraceptive Use and Breast Cancer Intrinsic-Like Subtypes, In Premenopausal Women Overall Stratified by Study Design (Corresponds to Results in eTable 15)

eTable 28. Number of Women Included in the Analysis on the Heterogeneity Between Studies Set Up Before and After 2000 for the Associations Between Estrogen-Progestin Therapy (EPT) Use and Breast Cancer Intrinsic-Like Subtypes, in Postmenopausal Women, Stratified by Body Mass Index (BMI) (Corresponds to Results in eTable 16)

eTable 29. Number of Women Included in the Analysis on the Heterogeneity Between Studies Set Up Before and After 2000 for the Associations Between Estrogen-Only Therapy (ET) Use and Breast Cancer Intrinsic-Like Subtypes, In Postmenopausal Women, Stratified by Body Mass Index (BMI) (Corresponds to Results in eTable 17)

eFigure 1. Odds Ratios and 95% CIs for Case-Control Analyses of Associations Between Estrogen-Progestin Therapy (EPT) Use and Breast Cancer Overall as well as Estrogen Receptor (ER)-Positive and ER-Negative Breast Cancer According to Body Mass Index (BMI)

eFigure 2. Odds Ratios and 95% CIs for Case-Control Analyses of Associations Between Estrogen Therapy (ET) Use and Breast Cancer Overall as well as Estrogen Receptor (ER)-Positive and ER-Negative Breast Cancer According to Body Mass Index (BMI)

eFigure 3. Odds Ratios and 95% CIs for Case-Control Analyses of Associations Between Oral Contraceptive (OC) Use and Breast Cancer Overall as well as Estrogen Receptor (ER)-Positive and ER-Negative Breast Cancer

eReferences

eAppendix 1. BCAC Funders

eAppendix 2. BCAC Additional Contributions

This supplemental material has been provided by the authors to give readers additional information about their work.

eMethods. Breast Cancer Risk Factors

Studies provided information on at least one reproductive risk factor, or exogenous hormone use and lifestyle risk factors. Data from studies was centrally quality-controlled and harmonized using a common data dictionary (1). Current use of OC and MHT were defined as use within 6 months prior to reference date. About 0.67% of the women reported current concomitant use of ET and EPT, which was accounted for in each variable for EP and E individually. Current BMI (at reference date) was provided by the studies or calculated from weight (in kilograms) and height (in meters).

Breast tumors were classified according to ER status (ER-positive/ER-negative) as well as invasive intrinsic-like subtypes using hormone receptor (HR) expression and histologic grade as proxy for proliferation106: luminal A-like (HR-positive, ERBB2-negative, grade 1&2), luminal B-like (HR-positive, ERBB2-negative, grade 3), luminal B-ERBB2-like (HR-positive, ERBB2-positive, any grade), ERBB2-enriched-like (HR-negative, ERBB2-positive, any grade), and triple-negative (HR-negative, ERBB2-negative, any grade).

© 2025 Le Cornet C et al. *JAMA Network Open*.

eTable 1. Description of Studies Included in the Analysis

					Breast cancer Cases with information on ER, PR, ERBB2 expression and grade in the tumors[1]				
Study, first author, year (reference)	Country	Recruitment years	Recruited Before (B) or After (A) 2000	Control subjects[2] (n = 71,072)	Luminal A-like (n = 12,405)	Luminal B-like (n = 2,832)	Luminal B-ERBB2-like (n = 3,088)	ERBB2-enriched-like (n = 1,498)	Triple negative (n = 3,530)
Prospective cohort									
AHS, Koutros, 2010 (2)	USA	1993-1997	B	1,237	48	7	2	4	8
CPSII, Calle, 2002 (3)	USA	1982	B	3,368	620	124	121	10	31
CTS, Bernstein, 2002 (4)	USA	1998-2008	A	1,621	.	.	.	.	101
EPIC, Riboli, 2002 (5)	France, Germany, Greece, Italy, Spain, The Netherlands, UK	1992-2000	B	3,688	522	157	166	39	58
KARMA, Li, 2016 (6)	Sweden	2010-2013	A	15,292	1,044	302	169	67	122
MCCS, Milne, 2017 (7)	Australia	1990-1994	B	1,365	578	147	99	55	139
MEC, Kolonel, 2000 (8)	USA	1993-2002	--	1,944	82	19	17	5	6
MISS, Olsson, 2003 (9)	Sweden	1990-1992	B	1,656	18	1	28	9	43
MMHS, Olson, 2012 (10)	USA	2003-2006	A	1,716	175	26	16	5	19
NHS, Hankinson, 1998 (11)	USA	1989-1990	B	3,568	456	108	136	41	118
NHS2, Tworoger, 2006 (12)	USA	1996-1999	B	2,164	513	111	110	38	95
PLCO, Pfeiffer, 2013 (13)	USA	1993-2001	B	3,070	1,065	173	142	54	144
SMC, Suzuki, 2005 (14)	Sweden	1987-2011	--	685	221	61	36	19	27
Population-based case-control study									
ABCFS, Dite, 2003 (15)	Australia	1992-1999, 1993-1998	B	1,398	.	.	6	3	4
BCEES, Fritschi, 2013 (16)	Australia	2009-2011	A	858	342	48	49	.	.
BCINIS, Rennert, 2010 (17)	Israel	1990-2000	B	900	966	204	144	84	232
CBCS, Grundy, 2013 (18)	Canada	2005-2009	A	1,179	381	120	239	69	76
CECILE, Menegaux, 2013 (19)	France	2005-2007	A	1,315	.	.	95	41	106
ESTHER, Widschwendter, 2008 (20)	Germany	2001-2003	A	766	79	26	37	16	24
GENICA, Pesch, 2005 (21)	Germany	2000-2004	A	1,015	303	111	138	61	74
GESBC, Chang-Claude, 2000 ((22)	Germany	1992-1998	B	1,381	.	.	.	.	.
KBCP, Hartikainen, 2005 (23)	Finland	1990-1995	B	536	276	49	78	30	52
LAABC, Wu, 2009 (24)	USA	1995-2007	--	1,047	.	.	.	.	.
MARIE, Flesch-Janys, 2008 (25)	Germany	2001-2005	A	7,337	1,864	371	455	212	392
MASTOS, Hadjisavvas, 2010 (26)	Cyprus	1999-2005	A	1,174	291	56	55	22	61
NBHS, Zheng, 2009 (27)	USA	2001-2011	A	1,075	61	31	88	99	217
NCBCS, Newman, 1995 (28)	USA	1993-1996, 1996-2000, 2008-2013	--	2,022	1,478	429	390	230	965
PBCS, Garcia-Closas, 2006 (29)	Poland	2000-2003	A	2,393	772	97	117	124	262
PROCAS, Evans, 2016 (30)	UK	2009-2014	A	1,745	250	54	31	9	21
SASBAC, Wedren, 2004 (31)	Sweden	1993-1995	B	1,515	.	.	.	.	.
SBCGS, Zheng, 2009 (27)	China	1996-2009	--	2,042	.	.	124	152	133

© 2025 Le Cornet C et al. *JAMA Network Open*.

1 Intrinsic-like subtype definitions: luminal A-like (HR-positive, ERBB2-negative, grade 1&2), luminal B-like (HR-positive, ERBB2-negative, grade 3), luminal B-ERBB2-like (HR-positive, ERBB2-positive, any grade), ERBB2-enriched-like (HR-negative, ERBB2-positive, any grade), and triple-negative (HR-negative, ERBB2-negative, any grade).
2 Control subjects in population-based studies were randomly selected from the same source population as the Cases and recruited during the same period of time.

© 2025 Le Cornet C et al. *JAMA Network Open*.

eTable 2. Baseline Characteristics of Women According to Pre- and Postmenopausal Status

	Premenopausal				Postmenopausal			
	Controls		Cases		Controls		Cases	
	N (%)		N (%)		N (%)		N (%)	
N	21,667		11,901		49,405		30,368	
**Age (mean, sd)**	46	(8)	47	(9)	62	(10)	64	(12)
**Breast cancer subtypes**								
ER+			8,624	(72.5)			24,751	(81.5)
ER-			3,277	(27.5)			5,617	(18.5)
Luminal A-like			2,590	(21.8)			9,815	(32.3)
Luminal B-like			753	(6.3)			2,079	(6.8)
Luminal B-ERBB2-like			896	(7.5)			2,192	(7.2)
ERBB2-enriched-like			438	(3.7)			1,060	(3.5)
Triple-negative			1,193	(10.0)			2,337	(7.7)
Unknown			6,031	(50.7)			12,885	(42.4)
**Body mass index (kg/m^2)**								
18.5-<25	11,868	(54.8)	6,305	(53.0)	23345	(47.3)	13,163	(43.3)
25-<30	5,552	(25.6)	2,891	(24.3)	15046	(30.5)	9,143	(30.1)
>=30	2,956	(13.6)	1,814	(15.2)	7446	(15.1)	5,702	(18.8)
Unknown	1,291	(6.0)	891	(7.5)	3568	(7.2)	2,360	(7.8)
**Parity**								
Nulliparous	3,186	(14.7)	2,072	(17.4)	5444	(11.0)	3,718	(12.2)
1	3,960	(18.3)	2,891	(24.3)	7286	(14.7)	4,963	(16.3)
2	8,912	(41.1)	4,153	(34.9)	17652	(35.7)	10,128	(33.4)
3+	5,324	(24.6)	2,564	(21.5)	18642	(37.7)	10,736	(35.4)
Unknown	285	(1.3)	221	(1.9)	381	(0.8)	823	(2.7)
**Age at first full pregnancy (years)**								
Nulliparous	3,186	(14.7)	2,072	(17.4)	5444	(11.0)	3,718	(12.2)
<20	1,498	(6.9)	1,032	(8.7)	5010	(10.1)	3,132	(10.3)
20-<25	5,465	(25.2)	2,759	(23.2)	17713	(35.9)	10,110	(33.3)
25-<30	6,182	(28.5)	3,272	(27.5)	12381	(25.1)	7,374	(24.3)
>=30	4,387	(20.2)	2,055	(17.3)	5222	(10.6)	3,289	(10.8)
Unknown	949	(4.4)	711	(6.0)	3635	(7.4)	2,745	(9.0)
**Age at menarche (years)**								
>14	3,057	(14.1)	1,574	(13.2)	8,984	(18.2)	4,984	(16.4)
14	4,041	(18.7)	1,974	(16.6)	9,110	(18.4)	5,170	(17.0)
13	5,640	(26.0)	3,089	(26.0)	12,365	(25.0)	7,648	(25.2)
<=12	8,259	(38.1)	4,892	(41.1)	15,313	(31.0)	10,023	(33.0)
Unknown	670	(3.1)	372	(3.1)	3,633	(7.4)	2,543	(8.4)
**Age at menopause (years)**								
Premenopause	21,667	(100)	11,901	(100)				
<50					19,399	(39.3)	12,230	(40.3)
50-<54					13,647	(27.6)	9,060	(29.8)
>=54					5,863	(11.9)	4,024	(13.3)
Unknown					10,496	(21.2)	5,054	(16.6)
**Breast feeding**								
No	4,407	(20.3)	3,518	(29.6)	8,287	(16.8)	6,788	(22.4)
Yes	8,406	(38.8)	5,198	(43.7)	17,253	(34.9)	11,509	(37.9)
Unknown	8,854	(40.9)	3,185	(26.8)	23,865	(48.3)	12,071	(39.7)
**Alcohol intakes**								
Non-drinker	4,990	(23.0)	2,859	(24.0)	12,184	(24.7)	7,930	(26.1)
<= 1 drink/day	7,627	(35.2)	2,227	(18.7)	14,017	(28.4)	6,106	(20.1)
<= 3 drinks/day	1,740	(8.0)	710	(6.0)	3,955	(8.0)	1,809	(6.0)
>3 drinks/day	207	(1.0)	133	(1.1)	601	(1.2)	332	(1.1)
Unknown	7,103	(32.8)	5,972	(50.2)	18,648	(37.7)	14,191	(46.7)

© 2025 Le Cornet C et al. *JAMA Network Open*.

Family history of breast cancer								
No	16,012	(73.9)	8,111	(68.2)	34,901	(70.6)	19,566	(64.4)
Yes	1,933	(8.9)	1,510	(12.7)	5,548	(11.2)	4,813	(15.8)
Unknown	3,722	(17.2)	2,280	(19.2)	8,956	(18.1)	5,989	(19.7)
Smoking status								
Never	11,267	(52.0)	6,143	(51.6)	24,534	(49.7)	15,357	(50.6)
Former	5,079	(23.4)	2,436	(20.5)	13,820	(28.0)	7,721	(25.4)
Current	3,482	(16.1)	2,183	(18.3)	6,032	(12.2)	3,789	(12.5)
Unknown	1,839	(8.5)	1,139	(9.6)	5,019	(10.2)	3,501	(11.5)
OC use								
Never	4,312	(19.9)	2,654	(22.3)	19,921	(40.3)	12,983	(42.8)
Former	7,437	(34.3)	5,493	(46.2)	11,595	(23.5)	8,272	(27.2)
Current	1,568	(7.2)	911	(7.7)	62	(0.1)	64	(0.2)
Unknown	8,350	(38.5)	2,843	(23.9)	17,827	(36.1)	9,049	(29.8)
OC duration of use								
Never	4,312	(19.9)	2,654	(22.3)	19,921	(40.3)	12,983	(42.8)
Former <5 years	2,587	(11.9)	2,036	(17.1)	4,396	(8.9)	3,243	(10.7)
Former >=5 years	3,751	(17.3)	3,194	(26.8)	6,278	(12.7)	4,490	(14.8)
Current <5 years	147	(0.7)	94	(0.8)	8	(0.0)	7	(0.0)
Current >=5 years	1,041	(4.8)	758	(6.4)	42	(0.1)	48	(0.2)
Unknown	9,829	(45.4)	3,165	(26.6)	18,760	(38.0)	9,597	(31.6)
OC time since last use								
Never	4,312	(19.9)	2,654	(22.3)	19,921	(40.3)	12,983	(42.8)
Current	1,568	(7.2)	911	(7.7)	62	(0.1)	64	(0.2)
<5 years	932	(4.3)	965	(8.1)	286	(0.6)	204	(0.7)
5-<10 years	880	(4.1)	803	(6.7)	551	(1.1)	391	(1.3)
>=10 years	4,375	(20.2)	3,421	(28.7)	9,430	(19.1)	6,780	(22.3)
Unknown	9,600	(44.3)	3,147	(26.4)	19,155	(38.8)	9,946	(32.8)
EPT use								
Never					29,497	(59.7)	16,882	(55.6)
Former					2,489	(5.0)	1,456	(4.8)
Current					3,073	(6.2)	2,808	(9.2)
Unknown					14,346	(29.0)	9,222	(30.4)
EPT duration of use								
Never					29,497	(59.7)	16,882	(55.6)
Former <5 years					1,018	(2.1)	554	(1.8)
Former >=5 years					1,164	(2.4)	697	(2.3)
Current <5 years					829	(1.7)	639	(2.1)
Current >=5 years					1,450	(2.9)	1,426	(4.7)
Unknown					15,447	(31.3)	10,170	(33.5)
EPT time since last use								
Never					29,497	(59.7)	16,882	(55.6)
Current					3,073	(6.2)	2,808	(9.2)
<5 years					1,085	(2.2)	622	(2.0)
5-<10 years					488	(1.0)	290	(1.0)
>=10 years					241	(0.5)	148	(0.5)
Unknown					15,021	(30.4)	9,618	(31.7)
ET use								
Never					28,666	(58.0)	17,162	(56.5)
Former					2,804	(5.7)	1,869	(6.2)
Current					3,374	(6.8)	1,967	(6.5)
Unknown					14,561	(29.5)	9,370	(30.9)
ET duration of use								
Never					28,666	(58.0)	17,162	(56.5)
Former <5 years					1,094	(2.2)	750	(2.5)

© 2025 Le Cornet C et al. *JAMA Network Open*.

Former >=5 years	954	(1.9)	691	(2.3)
Current <5 years	781	(1.6)	373	(1.2)
Current >=5 years	1,533	(3.1)	986	(3.2)
Unknown	16,377	(33.1)	10,406	(34.3)
ET time since last use				
Never	28,666	(58.0)	17,162	(56.5)
Current	3,374	(6.8)	1,967	(6.5)
<5 years	685	(1.4)	497	(1.6)
5-<10 years	492	(1.0)	330	(1.1)
>=10 years	455	(0.9)	394	(1.3)
Unknown	15,733	(31.8)	10,018	(33.0)

OC: oral contraceptive, EPT: estrogen-progestin therapy, ET: estrogen-only therapy

Intrinsic-like subtype definitions: luminal A-like (HR-positive, ERBB2-negative, grade 1&2), luminal B-like (HR-positive, ERBB2-negative, grade 3), luminal B-ERBB2-like (HR-positive, ERBB2-positive, any grade), ERBB2-enriched-like (HR-negative, ERBB2-positive, any grade), and triple-negative (HR-negative, ERBB2-negative, any grade). ER=estrogen receptor; PR=progesterone receptor.

© 2025 Le Cornet C et al. *JAMA Network Open*.

eTable 3. Odds Ratios and 95% CIs of Associations Between Estrogen-Progestin Therapy (EPT) Use and Breast Cancer Intrinsic-Like Subtypes, in Postmenopausal Women, Stratified by Body Mass Index (BMI)

	Luminal A-like	Luminal B-like	Luminal B-ERBB2-like	ERBB2-enriched-like	Triple-negative	Phet	ER+	ER-	Phet	Invasive breast cancer
Lean/normal										
Current use	2.51 (2.26,2.80)	1.47 (1.17,1.86)	1.95 (1.61,2.37)	0.77 (0.54,1.09)	1.03 (0.81,1.31)	***<.0001***	2.20 (2.03,2.39)	1.12 (0.96,1.32)	***<0.001***	1.97 (1.82,2.12)
Current, duration of use										
<5 years	2.05 (1.68,2.50)	1.11 (0.68,1.80)	2.03 (1.45,2.84)	0.73 (0.39,1.35)	0.81 (0.52,1.29)	***<0.001***	1.88 (1.62,2.18)	1.02 (0.78,1.34)	***<0.001***	1.67 (1.45,1.92)
≥5 years	2.65 (2.32,3.01)	1.52 (1.14,2.02)	1.83 (1.44,2.34)	0.70 (0.45,1.08)	1.13 (0.85,1.50)	***<0.001***	2.42 (2.18,2.69)	1.14 (0.93,1.39)	***<0.001***	2.11 (1.91,2.33)
Past use	1.21 (1.05,1.38)	1.14 (0.87,1.49)	1.01 (0.79,1.30)	0.98 (0.70,1.37)	1.16 (0.91,1.47)	0.50	1.09 (0.99,1.21)	1.10 (0.93,1.31)	0.86	1.10 (1.00,1.21)
Past, duration of use										
<5 years	1.07 (0.87,1.32)	1.12 (0.74,1.69)	1.08 (0.75,1.55)	1.10 (0.70,1.73)	1.16 (0.83,1.63)	0.99	0.97 (0.82,1.14)	1.14 (0.90,1.44)	0.29	1.02 (0.88,1.17)
≥5 years	1.31 (1.10,1.57)	1.25 (0.88,1.77)	0.88 (0.62,1.26)	0.87 (0.54,1.39)	1.16 (0.84,1.60)	0.15	1.16 (1.01,1.34)	0.99 (0.77,1.26)	0.19	1.12 (0.98,1.28)
Past, time since last use										
<5 years	1.16 (0.97,1.40)	1.19 (0.83,1.70)	0.94 (0.67,1.33)	1.02 (0.67,1.56)	1.39 (1.04,1.86)	0.44	1.07 (0.92,1.23)	1.24 (0.99,1.54)	0.28	1.12 (0.98,1.27)
5-10 years	1.24 (0.95,1.62)	1.21 (0.72,2.04)	1.15 (0.72,1.84)	1.15 (0.62,2.15)	1.09 (0.67,1.77)	0.99	1.10 (0.89,1.36)	0.98 (0.68,1.42)	0.54	1.07 (0.88,1.31)
≥10 years	0.94 (0.65,1.38)	0.53 (0.20,1.46)	0.84 (0.41,1.74)	0.86 (0.31,2.36)	0.52 (0.21,1.29)	0.67	0.78 (0.56,1.07)	0.71 (0.40,1.27)	0.80	0.76 (0.56,1.03)
Overweight										
Current use	2.18 (1.83,2.61)	1.05 (0.72,1.53)	1.83 (1.32,2.55)	1.27 (0.74,2.17)	0.96 (0.65,1.44)	***<.0001***	1.97 (1.73,2.24)	1.05 (0.81,1.36)	***<0.001***	1.77 (1.56,2.00)
Current, duration of use										
<5 years	1.81 (1.31,2.51)	0.48 (0.20,1.20)	1.65 (0.92,2.95)	1.19 (0.51,2.80)	0.39 (0.16,0.96)	***0.001***	1.63 (1.29,2.07)	0.69 (0.43,1.10)	***<0.001***	1.39 (1.11,1.74)
≥5 years	2.29 (1.78,2.94)	1.43 (0.89,2.29)	1.95 (1.26,3.03)	1.30 (0.61,2.76)	1.25 (0.75,2.05)	0.06	2.34 (1.92,2.84)	1.27 (0.89,1.79)	***<0.001***	2.08 (1.73,2.51)
Past use	1.37 (1.12,1.66)	0.99 (0.68,1.46)	0.72 (0.46,1.12)	0.92 (0.50,1.70)	0.96 (0.64,1.42)	***0.02***	1.03 (0.89,1.20)	0.80 (0.59,1.07)	0.07	0.98 (0.85,1.14)
Past, duration of use										
<5 years	1.24 (0.91,1.69)	0.98 (0.55,1.76)	0.70 (0.35,1.39)	0.97 (0.42,2.25)	0.60 (0.30,1.20)	0.23	0.96 (0.76,1.21)	0.63 (0.40,0.98)	0.06	0.88 (0.70,1.10)
≥5 years	1.50 (1.14,1.98)	0.84 (0.47,1.50)	0.66 (0.35,1.23)	0.91 (0.39,2.12)	1.11 (0.67,1.86)	***0.04***	1.09 (0.87,1.36)	0.89 (0.59,1.35)	0.33	1.04 (0.85,1.29)
Past, time since last use										
<5 years	1.57 (1.17,2.10)	0.92 (0.51,1.66)	0.73 (0.39,1.37)	1.22 (0.58,2.58)	0.94 (0.54,1.63)	0.06	1.21 (0.96,1.54)	0.90 (0.59,1.37)	0.14	1.14 (0.91,1.43)
5-10 years	1.29 (0.85,1.95)	0.80 (0.34,1.85)	0.74 (0.32,1.72)	0.60 (0.14,2.47)	0.62 (0.25,1.55)	0.33	0.87 (0.62,1.23)	0.58 (0.29,1.15)	0.24	0.81 (0.58,1.12)
≥10 years	0.81 (0.46,1.42)	0.86 (0.34,2.18)	0.54 (0.17,1.73)	0.84 (0.20,3.50)	0.63 (0.22,1.77)	0.96	0.67 (0.43,1.04)	0.60 (0.27,1.33)	0.83	0.65 (0.42,0.98)
Obese										
Current use	1.40 (1.02,1.92)	0.69 (0.34,1.39)	1.68 (1.01,2.78)	0.39 (0.10,1.64)	1.20 (0.69,2.09)	0.11	1.49 (1.19,1.86)	0.91 (0.58,1.42)	***0.01***	1.38 (1.11,1.71)
Current, duration of use										
<5 years	1.03 (0.57,1.85)	0.42 (0.10,1.77)	1.19 (0.46,3.07)	****	1.20 (0.54,2.65)	0.80	1.28 (0.86,1.91)	1.04 (0.55,1.95)	0.37	1.22 (0.84,1.78)
≥5 years	1.53 (0.95,2.47)	0.92 (0.36,2.34)	2.04 (1.01,4.16)	0.75 (0.18,3.19)	0.81 (0.31,2.09)	0.36	1.56 (1.09,2.23)	0.72 (0.35,1.47)	***0.02***	1.37 (0.97,1.94)
Past use	1.59 (1.21,2.09)	1.04 (0.60,1.79)	1.05 (0.60,1.84)	0.98 (0.42,2.31)	0.88 (0.52,1.50)	0.12	1.33 (1.07,1.65)	0.72 (0.47,1.12)	***0.004***	1.21 (0.98,1.49)
Past, duration of use										
<5 years	0.91 (0.56,1.47)	0.91 (0.41,2.03)	0.88 (0.37,2.08)	0.58 (0.14,2.41)	0.45 (0.18,1.15)	0.72	1.06 (0.75,1.49)	0.49 (0.25,0.97)	***0.02***	0.92 (0.67,1.29)
≥5 years	2.28 (1.54,3.38)	1.14 (0.51,2.56)	0.97 (0.41,2.30)	1.44 (0.50,4.14)	1.29 (0.64,2.62)	0.11	1.51 (1.08,2.12)	1.01 (0.55,1.86)	0.16	1.40 (1.01,1.95)
Past, time since last use										
<5 years	1.73 (1.05,2.87)	0.43 (0.10,1.80)	0.58 (0.18,1.91)	1.76 (0.61,5.11)	1.22 (0.53,2.78)	0.15	1.08 (0.71,1.65)	1.26 (0.66,2.38)	0.72	1.11 (0.75,1.65)
5-10 years	1.65 (0.99,2.76)	0.79 (0.28,2.27)	0.93 (0.32,2.67)	0.48 (0.07,3.61)	0.58 (0.20,1.67)	0.17	1.27 (0.81,1.99)	0.44 (0.17,1.14)	***0.02***	1.08 (0.69,1.68)

© 2025 Le Cornet C et al. *JAMA Network Open*.

≥10 years	1.47 (0.70,3.09)	1.49 (0.48,4.61)	2.12 (0.75,5.96)	0.80 (0.10,6.17)	0.46 (0.10,2.07)	0.45	1.85 (0.99,3.46)	0.47 (0.13,1.65)	***0.02***	1.51 (0.81,2.81)

The model was adjusted for reference age (age at diagnosis for cases, age at interview for controls), study, OC and ET use (never, former, current)

Phet: Heterogeneity by breast cancer subtypes (e.g. ER+ and ER- or across intrinsic-like subtypes) was evaluated by comparing models assuming the same association versus different associations with exogenous hormones.

Lean/normal (BMI 18.5-<25), overweight (BMI 25-<30), obese (BMI >=30)

Intrinsic-like subtype definitions: luminal A-like (HR-positive, ERBB2-negative, grade 1&2), luminal B-like (HR-positive, ERBB2-negative, grade 3), luminal B-ERBB2-like (HR-positive, ERBB2-positive, any grade), ERBB2-enriched-like (HR-negative, ERBB2-positive, any grade), and triple-negative (HR-negative, ERBB2-negative, any grade). ER=estrogen receptor; PR=progesterone receptor. ****Undefined estimate due to small number found in obese women for the association between current EPT use for less than 5 years with risk of ERBB2-enriched–like cancer.

© 2025 Le Cornet C et al. *JAMA Network Open*.

eTable 4. Odds Ratios and 95% CIs of Associations Between Menopausal Hormonal Therapy (MHT) Use and Breast Cancer Subtypes, in Postmenopausal Women Overall

	Luminal A-like	Luminal B-like	Luminal B-ERBB2-like	ERBB2-enriched-like	Triple-negative	Phet	ER+	ER-	Phet	Invasive breast cancer
EPT										
Current use	2.34 (2.15,2.55)	1.27 (1.05,1.52)	1.92 (1.65,2.24)	0.92 (0.70,1.21)	1.03 (0.85,1.25)	***<0.001***	2.05 (1.92,2.19)	1.09 (0.96,1.24)	***<0.001***	1.84 (1.73,1.96)
Current,										
<5 years	1.97 (1.68,2.30)	0.81 (0.54,1.22)	1.92 (1.46,2.52)	0.83 (0.51,1.35)	0.75 (0.52,1.07)	***<0.001***	1.78 (1.58,2.00)	0.95 (0.77,1.18)	***<0.001***	1.57 (1.41,1.76)
≥5 years	2.49 (2.23,2.77)	1.42 (1.13,1.79)	1.88 (1.54,2.30)	0.85 (0.59,1.21)	1.10 (0.87,1.39)	***<0.001***	2.29 (2.10,2.50)	1.12 (0.95,1.32)	***<0.001***	2.00 (1.84,2.18)
Past use	1.34 (1.21,1.48)	1.10 (0.90,1.34)	0.99 (0.81,1.21)	0.99 (0.75,1.31)	1.06 (0.88,1.28)	***0.001***	1.13 (1.05,1.22)	0.98 (0.85,1.13)	***0.01***	1.10 (1.02,1.18)
Past, duration of										
<5 years	1.12 (0.96,1.32)	1.06 (0.78,1.44)	1.06 (0.80,1.41)	1.06 (0.73,1.54)	0.90 (0.68,1.19)	0.68	1.01 (0.89,1.14)	0.92 (0.75,1.12)	0.21	0.99 (0.88,1.10)
≥5 years	1.53 (1.33,1.75)	1.12 (0.85,1.48)	0.87 (0.66,1.15)	0.93 (0.64,1.37)	1.14 (0.89,1.47)	***<0.001***	1.21 (1.08,1.34)	0.97 (0.80,1.18)	***0.02***	1.15 (1.04,1.28)
Past, time since										
<5 years	1.35 (1.17,1.56)	1.02 (0.76,1.36)	0.92 (0.70,1.22)	1.12 (0.80,1.59)	1.24 (0.97,1.57)	***0.04***	1.13 (1.01,1.27)	1.15 (0.96,1.38)	0.84	1.14 (1.03,1.27)
5-10 years	1.38 (1.14,1.69)	1.09 (0.74,1.62)	1.05 (0.73,1.52)	1.01 (0.59,1.71)	0.85 (0.58,1.26)	0.10	1.09 (0.93,1.29)	0.82 (0.61,1.10)	***0.04***	1.03 (0.88,1.20)
≥10 years	0.97 (0.73,1.29)	0.81 (0.46,1.43)	1.01 (0.62,1.64)	0.83 (0.39,1.77)	0.51 (0.28,0.95)	0.38	0.90 (0.72,1.14)	0.61 (0.40,0.95)	0.09	0.84 (0.67,1.04)
ET										
Current use	0.96 (0.87,1.06)	0.67 (0.55,0.83)	0.95 (0.79,1.14)	0.70 (0.52,0.94)	0.69 (0.57,0.84)	***<0.001***	0.95 (0.89,1.02)	0.77 (0.68,0.88)	***<0.001***	0.91 (0.86,0.97)
Current,										
<5 years	0.57 (0.45,0.72)	0.54 (0.35,0.84)	0.70 (0.48,1.04)	0.54 (0.30,0.94)	0.57 (0.40,0.82)	0.88	0.73 (0.63,0.84)	0.69 (0.55,0.87)	0.41	0.72 (0.63,0.82)
≥5 years	1.05 (0.92,1.19)	0.58 (0.43,0.78)	1.03 (0.81,1.29)	0.76 (0.53,1.08)	0.72 (0.57,0.92)	***<0.001***	0.99 (0.90,1.08)	0.76 (0.64,0.90)	***<0.001***	0.93 (0.85,1.02)
Past use	0.93 (0.85,1.02)	0.83 (0.69,1.00)	0.86 (0.72,1.03)	0.94 (0.72,1.22)	0.87 (0.73,1.04)	0.60	0.90 (0.84,0.96)	0.84 (0.74,0.96)	0.20	0.89 (0.83,0.95)
Past, duration of										
<5 years	0.94 (0.82,1.09)	0.91 (0.69,1.20)	0.89 (0.69,1.15)	0.86 (0.59,1.26)	1.00 (0.79,1.26)	0.92	0.91 (0.81,1.01)	0.85 (0.71,1.01)	0.30	0.89 (0.81,0.99)
≥5 years	1.11 (0.96,1.27)	0.84 (0.63,1.12)	0.91 (0.70,1.18)	1.09 (0.76,1.56)	0.82 (0.62,1.08)	0.09	0.96 (0.86,1.08)	0.91 (0.74,1.10)	0.41	0.95 (0.85,1.06)
Past, time since										
<5 years	1.12 (0.95,1.32)	1.07 (0.78,1.45)	1.11 (0.84,1.47)	1.09 (0.73,1.63)	0.94 (0.70,1.26)	0.76	1.13 (0.99,1.29)	1.01 (0.81,1.25)	0.22	1.10 (0.97,1.25)
5-10 years	0.97 (0.80,1.17)	0.69 (0.46,1.05)	0.77 (0.54,1.11)	1.15 (0.74,1.79)	0.86 (0.62,1.21)	0.33	0.87 (0.74,1.02)	0.82 (0.63,1.07)	0.60	0.85 (0.74,0.99)
≥10 years	0.91 (0.76,1.10)	0.83 (0.59,1.18)	0.78 (0.55,1.10)	0.71 (0.42,1.20)	0.89 (0.66,1.21)	0.81	0.86 (0.73,1.00)	0.86 (0.68,1.11)	0.94	0.85 (0.73,0.99)

The model was adjusted for reference age (age at diagnosis for cases, age at interview for controls), study, OC and the other MHT use (never, former, current), and BMI (lean, overweight, obese).

Phet: Heterogeneity by breast cancer subtypes (e.g. ER+ and ER- or across intrinsic-like subtypes) was evaluated by comparing models assuming the same association versus different associations with exogenous hormones.

Intrinsic-like subtype definitions: luminal A-like (HR-positive, ERBB2-negative, grade 1&2), luminal B-like (HR-positive, ERBB2-negative, grade 3), luminal B-ERBB2-like (HR-positive, ERBB2-positive, any grade), ERBB2-enriched-like (HR-negative, ERBB2-positive, any grade), and triple-negative (HR-negative, ERBB2-negative, any grade). ER=estrogen receptor; PR=progesterone receptor.

© 2025 Le Cornet C et al. *JAMA Network Open*.

eTable 5. Odds Ratios and 95% CIs of Associations Between Estrogen-Only (ET) Use and Breast Cancer Intrinsic-Like Subtypes, in Postmenopausal Women, Stratified by Body Mass Index (BMI)

	Luminal A-like	Luminal B-like	Luminal B-ERBB2-like	ERBB2-enriched-like	Triple-negative	Phet	ER+	ER-	Phet	Invasive breast cancer
Lean/normal										
Current use	1.16 (1.01,1.32)	0.85 (0.64,1.13)	0.96 (0.74,1.23)	0.58 (0.38,0.89)	0.73 (0.54,0.97)	***<0.001***	1.12 (1.02,1.23)	0.83 (0.69,0.99)	***<0.001***	1.06 (0.97,1.16)
Current, duration of use										
<5 years	0.74 (0.54,1.01)	0.65 (0.34,1.24)	1.05 (0.66,1.68)	0.32 (0.12,0.86)	0.71 (0.43,1.18)	0.27	0.95 (0.78,1.15)	0.78 (0.56,1.08)	0.21	0.91 (0.76,1.09)
≥5 years	1.29 (1.09,1.52)	0.86 (0.60,1.25)	0.96 (0.69,1.33)	0.67 (0.41,1.09)	0.73 (0.51,1.04)	***0.002***	1.17 (1.03,1.33)	0.81 (0.64,1.02)	***0.002***	1.09 (0.97,1.23)
Past use	0.99 (0.87,1.13)	0.83 (0.63,1.10)	0.90 (0.70,1.15)	0.85 (0.59,1.23)	1.08 (0.84,1.38)	0.46	0.99 (0.89,1.09)	1.03 (0.86,1.23)	0.69	1.00 (0.91,1.10)
Past, duration of use										
<5 years	1.04 (0.86,1.26)	0.81 (0.53,1.24)	0.81 (0.55,1.18)	0.81 (0.48,1.36)	1.27 (0.94,1.74)	0.16	0.99 (0.85,1.16)	1.08 (0.85,1.37)	0.55	1.01 (0.88,1.16)
≥5 years	1.12 (0.92,1.36)	0.86 (0.57,1.32)	0.99 (0.70,1.41)	1.03 (0.63,1.69)	0.96 (0.65,1.41)	0.77	1.03 (0.88,1.20)	1.04 (0.80,1.36)	0.93	1.03 (0.89,1.19)
Past, time since last use										
<5 years	1.15 (0.93,1.42)	1.17 (0.78,1.76)	1.41 (1.01,1.97)	1.09 (0.66,1.82)	1.08 (0.73,1.58)	0.85	1.20 (1.01,1.42)	1.13 (0.85,1.49)	0.71	1.19 (1.01,1.39)
5-10 years	1.01 (0.78,1.30)	0.54 (0.27,1.06)	0.56 (0.32,1.00)	1.14 (0.63,2.07)	1.10 (0.71,1.72)	0.11	0.83 (0.67,1.04)	0.96 (0.67,1.36)	0.46	0.86 (0.70,1.05)
≥10 years	0.97 (0.74,1.25)	0.65 (0.35,1.20)	0.55 (0.30,0.99)	0.49 (0.20,1.20)	1.23 (0.80,1.89)	0.06	0.84 (0.67,1.06)	1.07 (0.76,1.51)	0.16	0.89 (0.72,1.09)
Overweight										
Current use	0.75 (0.62,0.91)	0.49 (0.33,0.72)	0.83 (0.59,1.18)	0.84 (0.50,1.41)	0.59 (0.41,0.86)	0.19	0.76 (0.67,0.87)	0.70 (0.55,0.89)	0.31	0.75 (0.66,0.85)
Current, duration of use										
<5 years	0.29 (0.17,0.50)	0.52 (0.25,1.08)	0.47 (0.20,1.09)	1.03 (0.48,2.20)	0.33 (0.15,0.72)	0.12	0.50 (0.37,0.66)	0.56 (0.37,0.87)	0.78	0.52 (0.40,0.67)
≥5 years	0.81 (0.63,1.05)	0.33 (0.18,0.61)	0.93 (0.60,1.44)	0.66 (0.33,1.34)	0.69 (0.44,1.07)	0.06	0.80 (0.67,0.96)	0.72 (0.53,0.97)	0.40	0.78 (0.66,0.92)
Past use	0.79 (0.66,0.95)	0.81 (0.58,1.13)	0.77 (0.54,1.10)	0.94 (0.55,1.60)	0.69 (0.47,1.01)	0.89	0.82 (0.72,0.94)	0.69 (0.53,0.91)	0.16	0.80 (0.71,0.91)
Past, duration of use										
<5 years	0.80 (0.59,1.08)	1.11 (0.69,1.76)	1.14 (0.71,1.84)	0.95 (0.45,2.00)	0.99 (0.62,1.57)	0.78	0.92 (0.74,1.13)	0.79 (0.55,1.13)	0.34	0.89 (0.73,1.08)
≥5 years	0.85 (0.64,1.13)	0.66 (0.38,1.14)	0.57 (0.32,1.02)	0.81 (0.37,1.78)	0.39 (0.19,0.81)	0.21	0.79 (0.64,0.99)	0.59 (0.39,0.91)	0.17	0.75 (0.61,0.93)
Past, time since last use										
<5 years	0.73 (0.51,1.04)	0.86 (0.48,1.54)	0.54 (0.26,1.11)	0.76 (0.30,1.91)	0.49 (0.23,1.01)	0.64	0.87 (0.67,1.14)	0.65 (0.40,1.06)	0.21	0.82 (0.64,1.06)
5-10 years	0.95 (0.65,1.40)	0.80 (0.40,1.62)	0.86 (0.44,1.69)	1.05 (0.41,2.65)	0.83 (0.42,1.64)	0.95	0.88 (0.65,1.20)	0.83 (0.49,1.40)	0.80	0.86 (0.64,1.16)
≥10 years	0.76 (0.52,1.11)	0.87 (0.47,1.63)	1.01 (0.55,1.85)	1.00 (0.39,2.53)	0.88 (0.47,1.64)	0.96	0.82 (0.60,1.11)	0.88 (0.54,1.44)	0.77	0.82 (0.62,1.10)
Obese										
Current use	0.65 (0.50,0.85)	0.44 (0.25,0.76)	0.41 (0.23,0.74)	0.57 (0.25,1.26)	0.66 (0.43,1.00)	0.49	0.64 (0.53,0.77)	0.57 (0.41,0.79)	0.39	0.62 (0.52,0.75)
Current, duration of use										
<5 years	0.45 (0.25,0.80)	0.20 (0.05,0.81)	****	0.23 (0.03,1.69)	0.56 (0.27,1.17)	0.71	0.45 (0.30,0.67)	0.59 (0.34,1.02)	0.77	0.49 (0.34,0.69)
≥5 years	0.59 (0.40,0.85)	0.29 (0.13,0.68)	0.45 (0.22,0.94)	0.72 (0.30,1.71)	0.61 (0.36,1.02)	0.52	0.56 (0.43,0.73)	0.47 (0.30,0.72)	0.40	0.54 (0.42,0.69)
Past use	0.83 (0.66,1.04)	0.75 (0.50,1.12)	0.58 (0.36,0.92)	1.00 (0.56,1.80)	0.57 (0.39,0.85)	0.20	0.67 (0.56,0.80)	0.52 (0.38,0.71)	0.08	0.64 (0.54,0.76)
Past, duration of use										
<5 years	0.71 (0.50,1.02)	0.74 (0.42,1.32)	0.50 (0.25,1.02)	0.72 (0.30,1.70)	0.46 (0.26,0.80)	0.44	0.59 (0.45,0.77)	0.39 (0.25,0.62)	0.34	0.54 (0.42,0.70)
≥5 years	1.14 (0.81,1.62)	0.84 (0.44,1.58)	0.73 (0.37,1.45)	1.41 (0.65,3.08)	0.82 (0.47,1.44)	0.43	0.83 (0.61,1.11)	0.76 (0.48,1.20)	0.17	0.81 (0.61,1.07)
Past, time since last use										

© 2025 Le Cornet C et al. *JAMA Network Open*.

<5 years	1.52 (0.95,2.44)	0.82 (0.32,2.10)	0.65 (0.23,1.84)	1.74 (0.66,4.57)	1.26 (0.64,2.48)	0.35	1.16 (0.77,1.75)	1.22 (0.69,2.15)	0.99	1.17 (0.80,1.73)
5-10 years	0.77 (0.47,1.25)	0.78 (0.34,1.76)	0.55 (0.22,1.41)	1.00 (0.35,2.89)	0.42 (0.18,1.01)	0.61	0.72 (0.48,1.08)	0.44 (0.22,0.89)	0.14	0.65 (0.44,0.96)
≥10 years	0.81 (0.55,1.21)	0.89 (0.48,1.65)	0.74 (0.37,1.50)	0.66 (0.23,1.89)	0.44 (0.24,0.84)	0.37	0.77 (0.54,1.09)	0.40 (0.23,0.70)	***0.01***	0.66 (0.47,0.93)

The model was adjusted for reference age (age at diagnosis for cases, age at interview for controls), study, OC and EPT use (never, former, current).

Phet: Heterogeneity by breast cancer subtypes (e.g. ER+ and ER- or across intrinsic-like subtypes) was evaluated by comparing models assuming the same association versus different associations with exogenous hormones.

Lean/normal (BMI 18.5-<25), overweight (BMI 25-<30), obese (BMI >=30).

Intrinsic-like subtype definitions: luminal A-like (HR-positive, ERBB2-negative, grade 1&2), luminal B-like (HR-positive, ERBB2-negative, grade 3), luminal B-ERBB2-like (HR-positive, ERBB2-positive, any grade), ERBB2-enriched-like (HR-negative, ERBB2-positive, any grade), and triple-negative (HR-negative, ERBB2-negative, any grade). ER=estrogen receptor; PR=progesterone receptor. ****Undefined estimate due to small number found in obese women for the association between current ET use for less than 5 years with risk of ERBB2-enriched–like cancer.

© 2025 Le Cornet C et al. *JAMA Network Open*.

eTable 6. Odds Ratios and 95% CIs of Associations Between Oral Contraceptive Use and Breast Cancer Subtypes, in Premenopausal Women Overall

	Luminal A-like	Luminal B-like	Luminal B-ERBB2-like	ERBB2-enriched-like	Triple-negative	phet	ER+	ER-	Phet	Invasive breast cancer
Current use	1.27 (1.05,1.54)	1.46 (1.07,1.98)	1.14 (0.85,1.52)	1.04 (0.67,1.62)	1.09 (0.83,1.42)	0.14	1.11 (0.98,1.26)	1.01 (0.85,1.20)	***0.05***	1.09 (0.97,1.22)
Current, duration of use										
<5 years	0.73 (0.44,1.20)	1.25 (0.63,2.49)	0.83 (0.38,1.83)	0.48 (0.11,1.98)	0.72 (0.37,1.41)	0.51	0.92 (0.67,1.26)	0.81 (0.53,1.24)	0.46	0.90 (0.68,1.20)
≥5 years	1.37 (1.11,1.70)	1.52 (1.08,2.13)	1.28 (0.94,1.76)	1.17 (0.73,1.86)	1.13 (0.85,1.50)	0.39	1.18 (1.03,1.35)	1.04 (0.86,1.25)	***0.05***	1.14 (1.01,1.29)
Past use	0.91 (0.81,1.03)	1.03 (0.83,1.28)	1.09 (0.90,1.33)	1.15 (0.86,1.53)	1.18 (0.98,1.41)	0.33	1.08 (1.00,1.17)	1.24 (1.10,1.39)	0.24	1.12 (1.04,1.20)
Past, duration of use										
<5 years	0.88 (0.76,1.02)	1.08 (0.84,1.38)	1.18 (0.93,1.48)	0.96 (0.68,1.37)	1.05 (0.85,1.29)	0.17	1.06 (0.96,1.17)	1.09 (0.95,1.26)	0.82	1.07 (0.98,1.17)
≥5 years	0.87 (0.76,1.01)	0.97 (0.76,1.24)	1.12 (0.90,1.40)	1.34 (0.98,1.83)	1.24 (1.02,1.51)	***0.01***	1.12 (1.02,1.23)	1.34 (1.18,1.52)	0.07	1.17 (1.08,1.28)
Past, time since last use										
<5 years	1.10 (0.87,1.39)	1.27 (0.88,1.85)	1.50 (1.09,2.08)	2.33 (1.55,3.51)	1.75 (1.33,2.29)	***0.04***	1.51 (1.32,1.72)	1.88 (1.58,2.22)	0.11	1.64 (1.46,1.85)
>5-10 years	1.11 (0.89,1.39)	1.11 (0.77,1.60)	1.45 (1.06,1.98)	1.26 (0.79,2.01)	1.62 (1.24,2.11)	0.35	1.30 (1.13,1.49)	1.51 (1.27,1.81)	0.39	1.36 (1.21,1.54)
≥10 years	0.84 (0.74,0.96)	0.92 (0.74,1.16)	1.02 (0.83,1.25)	0.98 (0.72,1.34)	1.03 (0.85,1.25)	0.72	1.01 (0.92,1.10)	1.10 (0.97,1.25)	0.66	1.03 (0.95,1.11)

The model was adjusted for reference age (age at diagnosis for cases, age at interview for controls), study, and BMI (lean, overweight, obese).

Phet:Heterogeneity by breast cancer subtypes (e.g. ER+ and ER- or across intrinsic-like subtypes) was evaluated by comparing models assuming the same association versus different associations with exogenous hormones.

Intrinsic-like subtype definitions: luminal A-like (HR-positive, ERBB2-negative, grade 1&2), luminal B-like (HR-positive, ERBB2-negative, grade 3), luminal B-ERBB2-like (HR-positive, ERBB2-positive, any grade), ERBB2-enriched-like (HR-negative, ERBB2-positive, any grade), and triple-negative (HR-negative, ERBB2-negative, any grade). ER=estrogen receptor; PR=progesterone receptor.

© 2025 Le Cornet C et al. *JAMA Network Open*.

eTable 7. Fully Adjusted Odds Ratios and 95% CIs of Associations Between Estrogen-Progestin Therapy (EPT) Use and Breast Cancer Intrinsic-Like Subtypes, in Postmenopausal Women, Stratified by Body Mass Index (BMI)

	Luminal A-like	Luminal B-like	Luminal B-ERBB2-like	ERBB2-enriched-like	Triple-negative	Phet	ER+	ER-	Phet	Invasive breast cancer
Lean/normal										
Current use	2.46 (2.21,2.74)	1.43 (1.13,1.81)	1.90 (1.56,2.32)	0.75 (0.53,1.06)	1.03 (0.81,1.32)	***<0.001***	2.13 (1.96,2.31)	1.11 (0.94,1.30)	***<0.001***	1.91 (1.77,2.06)
Current, duration of use										
<5 years	1.94 (1.59,2.37)	1.05 (0.65,1.71)	1.91 (1.36,2.68)	0.67 (0.36,1.25)	0.81 (0.51,1.28)	***<0.001***	1.75 (1.51,2.04)	0.99 (0.75,1.30)	***<0.001***	1.56 (1.36,1.80)
≥5 years	2.60 (2.28,2.97)	1.48 (1.11,1.97)	1.78 (1.40,2.28)	0.68 (0.44,1.06)	1.14 (0.85,1.52)	***<0.001***	2.36 (2.12,2.63)	1.13 (0.92,1.38)	***<0.001***	2.06 (1.86,2.28)
Past use	1.16 (1.02,1.33)	1.10 (0.84,1.45)	0.98 (0.76,1.26)	0.94 (0.67,1.32)	1.15 (0.90,1.46)	0.53	1.06 (0.95,1.18)	1.09 (0.92,1.30)	0.84	1.07 (0.97,1.17)
Past, duration of use										
<5 years	1.03 (0.83,1.27)	1.08 (0.71,1.64)	1.04 (0.72,1.50)	1.05 (0.67,1.65)	1.16 (0.83,1.62)	0.97	0.93 (0.79,1.09)	1.12 (0.89,1.42)	0.16	0.98 (0.85,1.13)
≥5 years	1.27 (1.06,1.51)	1.21 (0.85,1.72)	0.85 (0.59,1.20)	0.83 (0.52,1.33)	1.14 (0.82,1.58)	0.14	1.12 (0.98,1.30)	0.98 (0.77,1.25)	0.27	1.09 (0.95,1.24)
Past, time since last use										
<5 years	1.11 (0.92,1.34)	1.15 (0.80,1.65)	0.89 (0.63,1.26)	0.95 (0.62,1.45)	1.36 (1.01,1.82)	0.34	1.01 (0.87,1.18)	1.21 (0.97,1.51)	0.18	1.07 (0.93,1.22)
5-10 years	1.22 (0.93,1.59)	1.18 (0.70,1.98)	1.14 (0.71,1.82)	1.14 (0.61,2.12)	1.11 (0.68,1.80)	0.99	1.09 (0.87,1.35)	1.00 (0.69,1.45)	0.64	1.07 (0.87,1.31)
≥10 years	0.91 (0.62,1.34)	0.52 (0.19,1.43)	0.82 (0.40,1.70)	0.88 (0.32,2.43)	0.51 (0.21,1.27)	0.71	0.76 (0.55,1.05)	0.72 (0.41,1.29)	0.88	0.75 (0.56,1.02)
Overweight										
Current use	2.18 (1.82,2.61)	1.08 (0.74,1.58)	1.81 (1.30,2.52)	1.30 (0.75,2.23)	1.00 (0.67,1.50)	***<0.001***	1.96 (1.72,2.24)	1.08 (0.83,1.41)	***<0.001***	1.77 (1.56,2.01)
Current, duration of use										
<5 years	1.79 (1.29,2.47)	0.50 (0.20,1.24)	1.62 (0.90,2.89)	1.18 (0.50,2.79)	0.40 (0.16,0.99)	***0.001***	1.60 (1.26,2.04)	0.71 (0.44,1.13)	***<0.001***	1.37 (1.09,1.73)
≥5 years	2.29 (1.78,2.95)	1.46 (0.91,2.34)	1.92 (1.23,2.98)	1.33 (0.63,2.83)	1.29 (0.78,2.14)	0.07	2.33 (1.92,2.84)	1.31 (0.92,1.86)	***0.001***	2.09 (1.73,2.52)
Past use	1.34 (1.09,1.63)	1.00 (0.68,1.48)	0.71 (0.46,1.11)	0.92 (0.50,1.70)	0.97 (0.65,1.44)	***0.04***	1.02 (0.87,1.19)	0.81 (0.60,1.09)	0.13	0.97 (0.84,1.13)
Past, duration of use										
<5 years	1.19 (0.87,1.63)	0.96 (0.54,1.73)	0.68 (0.34,1.34)	0.96 (0.41,2.22)	0.60 (0.30,1.19)	0.26	0.94 (0.74,1.19)	0.64 (0.41,1.01)	0.10	0.87 (0.69,1.09)
≥5 years	1.49 (1.13,1.96)	0.85 (0.47,1.53)	0.66 (0.35,1.25)	0.91 (0.39,2.13)	1.14 (0.68,1.91)	0.06	1.07 (0.85,1.34)	0.89 (0.59,1.35)	0.40	1.03 (0.83,1.27)
Past, time since last use										
<5 years	1.53 (1.14,2.05)	0.93 (0.52,1.67)	0.72 (0.39,1.36)	1.21 (0.57,2.57)	0.96 (0.55,1.67)	0.10	1.17 (0.92,1.49)	0.89 (0.59,1.36)	0.20	1.11 (0.89,1.39)
5-10 years	1.29 (0.85,1.96)	0.83 (0.36,1.93)	0.72 (0.31,1.67)	0.60 (0.14,2.48)	0.62 (0.25,1.54)	0.32	0.86 (0.61,1.22)	0.57 (0.29,1.15)	0.24	0.80 (0.58,1.11)
≥10 years	0.77 (0.43,1.35)	0.82 (0.32,2.10)	0.53 (0.16,1.72)	0.81 (0.19,3.42)	0.60 (0.21,1.70)	0.97	0.64 (0.41,1.01)	0.58 (0.26,1.31)	0.82	0.62 (0.41,0.95)
Obese										
Current use	1.41 (1.02,1.93)	0.66 (0.33,1.33)	1.65 (0.99,2.75)	0.39 (0.09,1.63)	1.27 (0.73,2.23)	0.12	1.50 (1.19,1.88)	0.97 (0.62,1.52)	***0.03***	1.40 (1.13,1.74)
Current, duration of use										
<5 years	1.04 (0.57,1.88)	0.41 (0.10,1.71)	1.19 (0.46,3.08)	<0.01 (<0.01,****)	1.33 (0.60,2.99)	0.74	1.34 (0.90,2.01)	1.17 (0.61,2.22)	0.56	1.30 (0.89,1.91)
≥5 years	1.58 (0.98,2.56)	0.90 (0.35,2.30)	2.01 (0.98,4.12)	0.75 (0.18,3.19)	0.84 (0.33,2.18)	0.40	1.62 (1.13,2.33)	0.76 (0.37,1.56)	***0.03***	1.43 (1.01,2.04)
Past use	1.52 (1.16,2.01)	1.04 (0.60,1.80)	1.01 (0.58,1.78)	0.99 (0.42,2.34)	0.87 (0.51,1.50)	0.19	1.31 (1.05,1.64)	0.76 (0.49,1.18)	***0.01***	1.20 (0.97,1.49)
Past, duration of use										
<5 years	0.89 (0.54,1.45)	0.95 (0.43,2.13)	0.87 (0.36,2.06)	0.62 (0.15,2.61)	0.46 (0.18,1.18)	0.78	1.13 (0.80,1.59)	0.55 (0.28,1.09)	***0.03***	0.99 (0.71,1.39)
≥5 years	2.23 (1.50,3.32)	1.15 (0.51,2.59)	0.95 (0.40,2.26)	1.43 (0.49,4.11)	1.32 (0.65,2.69)	0.14	1.49 (1.06,2.10)	1.06 (0.58,1.94)	0.22	1.39 (1.00,1.94)
Past, time since last use										
<5 years	1.74 (1.05,2.89)	0.44 (0.10,1.83)	0.58 (0.18,1.89)	1.82 (0.63,5.32)	1.26 (0.55,2.90)	0.15	1.08 (0.70,1.66)	1.32 (0.69,2.52)	0.59	1.12 (0.75,1.67)
5-10 years	1.49 (0.89,2.52)	0.80 (0.28,2.31)	0.88 (0.31,2.54)	0.48 (0.06,3.58)	0.59 (0.20,1.71)	0.27	1.19 (0.76,1.88)	0.44 (0.17,1.16)	***0.03***	1.02 (0.65,1.59)

© 2025 Le Cornet C et al. *JAMA Network Open*.

≥10 years	1.42 (0.67,2.99)	1.49 (0.48,4.59)	2.05 (0.72,5.82)	0.81 (0.10,6.23)	0.42 (0.09,1.89)	0.43	1.80 (0.96,3.37)	0.43 (0.12,1.53)	***0.02***	1.45 (0.77,2.70)

Adjusted for age at reference date, study, OC and ET use (never, former, current), alcohol consumption (g per day), family history of breast cancer, breastfeeding duration (months), age at first birth, parity, age at menarche, and, in postmenopausal women, age at menopause.

Phet: Heterogeneity by breast cancer subtypes (e.g. ER+ and ER- or across intrinsic-like subtypes) was evaluated by comparing models assuming the same association versus different associations with exogenous hormones.

Lean/normal (BMI 18.5-<25), overweight (BMI 25-<30), obese (BMI >=30).

Intrinsic-like subtype definitions: luminal A-like (HR-positive, ERBB2-negative, grade 1&2), luminal B-like (HR-positive, ERBB2-negative, grade 3), luminal B-ERBB2-like (HR-positive, ERBB2-positive, any grade), ERBB2-enriched-like (HR-negative, ERBB2-positive, any grade), and triple-negative (HR-negative, ERBB2-negative, any grade). ER=estrogen receptor; PR=progesterone receptor.

© 2025 Le Cornet C et al. *JAMA Network Open*.

eTable 8. Fully Adjusted Odds Ratios and 95% CIs of Associations Between Estrogen-Only Therapy (ET) Use and Breast Cancer Intrinsic-Like Subtypes, in Postmenopausal Women, Stratified by Body Mass Index (BMI)

	Luminal A-like	Luminal B-like	Luminal B-ERBB2-like	ERBB2-enriched-like	Triple-negative	Phet	ER+	ER-	Phet	Invasive breast cancer
Lean/normal										
Current use	1.18 (1.03,1.35)	0.85 (0.64,1.14)	0.97 (0.76,1.26)	0.61 (0.39,0.93)	0.73 (0.55,0.98)	***<0.001***	1.15 (1.05,1.27)	0.84 (0.70,1.01)	***<0.001***	1.09 (1.00,1.19)
Current, duration of use										
<5 years	0.76 (0.56,1.04)	0.67 (0.35,1.27)	1.06 (0.66,1.70)	0.32 (0.12,0.87)	0.73 (0.44,1.21)	0.27	0.98 (0.80,1.19)	0.79 (0.57,1.10)	0.21	0.94 (0.78,1.12)
≥5 years	1.31 (1.10,1.55)	0.86 (0.59,1.26)	0.96 (0.69,1.34)	0.70 (0.42,1.15)	0.73 (0.51,1.05)	***0.001***	1.20 (1.06,1.37)	0.82 (0.65,1.03)	***0.001***	1.12 (0.99,1.26)
Past use	0.99 (0.87,1.13)	0.82 (0.62,1.09)	0.90 (0.70,1.14)	0.86 (0.59,1.24)	1.07 (0.83,1.37)	0.51	0.99 (0.89,1.09)	1.03 (0.86,1.23)	0.74	1.00 (0.91,1.09)
Past, duration of use										
<5 years	1.03 (0.85,1.25)	0.81 (0.53,1.25)	0.81 (0.55,1.18)	0.82 (0.49,1.38)	1.26 (0.92,1.72)	0.19	0.99 (0.85,1.15)	1.08 (0.85,1.36)	0.52	1.01 (0.87,1.16)
≥5 years	1.13 (0.93,1.37)	0.85 (0.56,1.30)	1.00 (0.70,1.41)	1.04 (0.63,1.71)	0.95 (0.65,1.40)	0.73	1.04 (0.89,1.22)	1.05 (0.80,1.37)	0.99	1.04 (0.90,1.20)
Past, time since last use										
<5 years	1.17 (0.94,1.44)	1.19 (0.79,1.79)	1.42 (1.02,1.98)	1.11 (0.66,1.84)	1.09 (0.74,1.60)	0.86	1.22 (1.03,1.45)	1.14 (0.87,1.51)	0.66	1.21 (1.03,1.42)
5-10 years	1.02 (0.79,1.32)	0.54 (0.27,1.06)	0.56 (0.32,1.00)	1.13 (0.62,2.06)	1.09 (0.70,1.70)	0.11	0.84 (0.67,1.05)	0.97 (0.68,1.38)	0.51	0.87 (0.71,1.07)
≥10 years	0.95 (0.73,1.24)	0.64 (0.34,1.19)	0.55 (0.31,1.00)	0.51 (0.21,1.25)	1.20 (0.78,1.84)	0.08	0.84 (0.67,1.06)	1.07 (0.76,1.51)	0.18	0.89 (0.72,1.09)
Overweight										
Current use	0.76 (0.63,0.92)	0.49 (0.33,0.72)	0.81 (0.57,1.15)	0.86 (0.51,1.45)	0.59 (0.40,0.86)	0.16	0.78 (0.68,0.88)	0.72 (0.57,0.93)	0.43	0.76 (0.68,0.87)
Current, duration of use										
<5 years	0.30 (0.17,0.51)	0.54 (0.26,1.12)	0.47 (0.20,1.09)	1.08 (0.50,2.31)	0.33 (0.15,0.72)	0.11	0.51 (0.38,0.69)	0.59 (0.38,0.91)	0.70	0.54 (0.42,0.70)
≥5 years	0.81 (0.63,1.05)	0.32 (0.17,0.60)	0.89 (0.58,1.38)	0.67 (0.33,1.37)	0.67 (0.43,1.06)	0.06	0.81 (0.67,0.97)	0.74 (0.54,1.01)	0.53	0.78 (0.66,0.93)
Past use	0.79 (0.66,0.96)	0.79 (0.57,1.11)	0.75 (0.52,1.07)	0.92 (0.54,1.57)	0.68 (0.46,0.99)	0.91	0.82 (0.72,0.94)	0.70 (0.54,0.92)	0.23	0.80 (0.71,0.91)
Past, duration of use										
<5 years	0.79 (0.58,1.07)	1.07 (0.67,1.71)	1.11 (0.69,1.78)	0.91 (0.44,1.92)	0.97 (0.61,1.54)	0.73	0.90 (0.73,1.11)	0.80 (0.56,1.15)	0.46	0.87 (0.72,1.07)
≥5 years	0.87 (0.65,1.15)	0.67 (0.39,1.17)	0.56 (0.31,1.00)	0.81 (0.37,1.79)	0.39 (0.19,0.81)	0.21	0.81 (0.65,1.00)	0.61 (0.40,0.94)	0.20	0.77 (0.62,0.95)
Past, time since last use										
<5 years	0.73 (0.51,1.05)	0.86 (0.48,1.56)	0.51 (0.25,1.07)	0.76 (0.30,1.91)	0.47 (0.22,0.97)	0.56	0.88 (0.67,1.16)	0.65 (0.40,1.06)	0.19	0.83 (0.64,1.07)
5-10 years	0.95 (0.64,1.39)	0.80 (0.39,1.61)	0.85 (0.43,1.68)	1.02 (0.40,2.59)	0.84 (0.42,1.65)	0.98	0.87 (0.64,1.19)	0.84 (0.50,1.41)	0.84	0.86 (0.64,1.15)
≥10 years	0.77 (0.52,1.12)	0.86 (0.46,1.61)	0.99 (0.54,1.82)	0.98 (0.39,2.50)	0.88 (0.47,1.65)	0.95	0.81 (0.60,1.10)	0.88 (0.54,1.44)	0.74	0.82 (0.61,1.09)
Obese										
Current use	0.69 (0.52,0.90)	0.44 (0.25,0.76)	0.43 (0.24,0.77)	0.55 (0.25,1.22)	0.65 (0.43,0.99)	0.40	0.70 (0.58,0.84)	0.61 (0.44,0.86)	0.32	0.68 (0.57,0.81)
Current, duration of use										
<5 years	0.48 (0.27,0.85)	0.20 (0.05,0.84)	<0.01 (<0.01,****)	0.22 (0.03,1.62)	0.56 (0.27,1.19)	0.69	0.50 (0.33,0.74)	0.64 (0.37,1.13)	0.50	0.54 (0.37,0.77)
≥5 years	0.64 (0.44,0.94)	0.30 (0.13,0.69)	0.48 (0.23,1.01)	0.72 (0.30,1.72)	0.61 (0.36,1.03)	0.50	0.63 (0.48,0.83)	0.51 (0.33,0.80)	0.27	0.61 (0.47,0.78)
Past use	0.85 (0.67,1.07)	0.74 (0.49,1.11)	0.57 (0.36,0.92)	0.98 (0.54,1.76)	0.55 (0.37,0.83)	0.14	0.70 (0.58,0.84)	0.54 (0.39,0.75)	0.08	0.67 (0.56,0.79)
Past, duration of use										
<5 years	0.75 (0.53,1.07)	0.76 (0.43,1.35)	0.51 (0.25,1.04)	0.73 (0.31,1.74)	0.46 (0.26,0.81)	0.37	0.67 (0.51,0.88)	0.44 (0.28,0.69)	***0.04***	0.61 (0.47,0.79)
≥5 years	1.15 (0.81,1.65)	0.82 (0.43,1.54)	0.73 (0.37,1.45)	1.36 (0.62,2.98)	0.79 (0.45,1.39)	0.40	0.85 (0.63,1.15)	0.77 (0.49,1.23)	0.61	0.83 (0.62,1.10)
Past, time since last use										
<5 years	1.51 (0.93,2.44)	0.78 (0.30,2.01)	0.64 (0.23,1.82)	1.67 (0.63,4.41)	1.26 (0.64,2.50)	0.34	1.14 (0.75,1.74)	1.23 (0.70,2.19)	0.89	1.16 (0.78,1.72)

© 2025 Le Cornet C et al. *JAMA Network Open*.

5-10 years	0.74 (0.45,1.21)	0.77 (0.34,1.75)	0.54 (0.21,1.40)	0.94 (0.33,2.74)	0.40 (0.17,0.96)	0.59	0.71 (0.48,1.07)	0.44 (0.22,0.88)	0.14	0.64 (0.44,0.95)
≥10 years	0.83 (0.55,1.24)	0.87 (0.47,1.63)	0.73 (0.36,1.48)	0.63 (0.22,1.83)	0.41 (0.22,0.78)	0.28	0.78 (0.55,1.11)	0.38 (0.22,0.68)	***0.009***	0.67 (0.47,0.94)

Adjusted for age at reference date, study, OC and EPT use (never, former, current), alcohol consumption (g per day), family history of breast cancer, breastfeeding duration (months), age at first birth, parity, age at menarche, and, in postmenopausal women, age at menopause.

Phet: Heterogeneity by breast cancer subtypes (e.g. ER+ and ER- or across intrinsic-like subtypes) was evaluated by comparing models assuming the same association versus different associations with exogenous hormones.

Lean/normal (BMI 18.5-<25), overweight (BMI 25-<30), obese (BMI >=30).

Intrinsic-like subtype definitions: luminal A-like (HR-positive, ERBB2-negative, grade 1&2), luminal B-like (HR-positive, ERBB2-negative, grade 3), luminal B-ERBB2-like (HR-positive, ERBB2-positive, any grade), ERBB2-enriched-like (HR-negative, ERBB2-positive, any grade), and triple-negative (HR-negative, ERBB2-negative, any grade). ER=estrogen receptor; PR=progesterone receptor.

© 2025 Le Cornet C et al. *JAMA Network Open*.

eTable 9. Fully Adjusted Odds Ratios and 95% CIs of Associations Between Oral Contraceptive Use and Breast Cancer Subtypes, in Premenopausal Women Overall

	Luminal A-like	Luminal B-like	Luminal B-ERBB2-like	ERBB2-enriched-like	Triple-negative	Phet	ER+	ER-	Phet	Invasive breast cancer
Current use	1.14 (0.94,1.39)	1.34 (0.98,1.84)	1.04 (0.77,1.39)	0.98 (0.63,1.52)	1.09 (0.83,1.42)	0.36	1.02 (0.90,1.15)	0.96 (0.80,1.14)	0.23	1.00 (0.89,1.12)
Current, duration										
<5 years	0.67 (0.40,1.10)	1.21 (0.61,2.41)	0.77 (0.35,1.69)	0.45 (0.11,1.90)	0.76 (0.39,1.48)	0.52	0.87 (0.64,1.20)	0.80 (0.52,1.23)	0.64	0.87 (0.65,1.15)
≥5 years	1.22 (0.98,1.52)	1.39 (0.99,1.96)	1.16 (0.84,1.58)	1.09 (0.68,1.75)	1.12 (0.85,1.49)	0.71	1.09 (0.95,1.25)	0.99 (0.82,1.20)	0.22	1.06 (0.94,1.20)
Past use	0.89 (0.78,1.01)	1.02 (0.82,1.27)	1.08 (0.89,1.31)	1.11 (0.83,1.49)	1.16 (0.97,1.39)	0.16	1.06 (0.97,1.15)	1.19 (1.06,1.34)	0.14	1.09 (1.01,1.18)
Past, duration of										
<5 years	0.88 (0.76,1.02)	1.10 (0.86,1.42)	1.20 (0.95,1.52)	0.94 (0.66,1.33)	1.02 (0.82,1.26)	0.10	1.09 (0.99,1.21)	1.08 (0.94,1.24)	0.77	1.09 (1.00,1.20)
≥5 years	0.83 (0.72,0.96)	0.94 (0.74,1.20)	1.08 (0.87,1.35)	1.28 (0.94,1.76)	1.23 (1.01,1.50)	***0.004***	1.09 (0.99,1.19)	1.30 (1.14,1.47)	***0.02***	1.14 (1.05,1.24)
Past, time since										
<5 years	1.04 (0.82,1.32)	1.23 (0.85,1.79)	1.42 (1.03,1.96)	2.24 (1.48,3.37)	1.74 (1.32,2.28)	***0.02***	1.43 (1.25,1.63)	1.80 (1.52,2.13)	***0.03***	1.56 (1.38,1.76)
>5-10 years	1.05 (0.84,1.32)	1.08 (0.75,1.57)	1.38 (1.01,1.89)	1.21 (0.76,1.92)	1.62 (1.24,2.12)	0.17	1.22 (1.07,1.40)	1.46 (1.22,1.74)	0.14	1.30 (1.15,1.47)
≥10 years	0.84 (0.73,0.96)	0.93 (0.74,1.17)	1.02 (0.82,1.25)	0.94 (0.69,1.29)	1.00 (0.82,1.21)	0.52	1.00 (0.91,1.09)	1.06 (0.93,1.21)	0.47	1.01 (0.93,1.10)

Adjusted for reference age (age at diagnosis for cases, age at interview for controls), study, and BMI (lean, overweight, obese), alcohol consumption (g per day), family history of breast cancer, breastfeeding duration (months), age at first birth, parity, and age at menarche.

Phet: Heterogeneity by breast cancer subtypes (e.g. ER+ and ER- or across intrinsic-like subtypes) was evaluated by comparing models assuming the same association versus different associations with exogenous hormones.

Intrinsic-like subtype definitions: luminal A-like (HR-positive, ERBB2-negative, grade 1&2), luminal B-like (HR-positive, ERBB2-negative, grade 3), luminal B-ERBB2-like (HR-positive, ERBB2-positive, any grade), ERBB2-enriched-like (HR-negative, ERBB2-positive, any grade), and triple-negative (HR-negative, ERBB2-negative, any grade). ER=estrogen receptor; PR=progesterone receptor.

© 2025 Le Cornet C et al. *JAMA Network Open*.

eTable 10. Odds Ratios and 95% CIs of Associations Between Estrogen-Progestin Therapy (EPT) Use and Breast Cancer Intrinsic-Like Subtypes, in Postmenopausal Women, Stratified by Body Mass Index (BMI) in a Restricted Set of Studies With at Least 10 Cases Reporting EPT Use

	Luminal A-like	Luminal B-like	Luminal B-ERBB2-like	ERBB2-enriched-like	Triple-negative	Phet	ER+	ER-	Phet	Invasive breast cancer
Lean/normal										
Current use	2.48 (2.22,2.76)	1.46 (1.15,1.86)	1.87 (1.53,2.27)	0.76 (0.54,1.08)	1.01 (0.80,1.29)	***<0.001***	2.16 (1.99,2.35)	1.12 (0.95,1.32)	***<0.001***	1.94 (1.79,2.10)
Current, duration										
<5 years	2.02 (1.66,2.47)	1.13 (0.70,1.84)	1.94 (1.39,2.73)	0.71 (0.38,1.32)	0.79 (0.50,1.26)	***<0.001***	1.87 (1.61,2.17)	1.01 (0.76,1.33)	***<0.001***	1.66 (1.44,1.91)
≥5 years	2.64 (2.32,3.01)	1.55 (1.16,2.06)	1.80 (1.41,2.30)	0.69 (0.45,1.07)	1.14 (0.86,1.52)	***<0.001***	2.42 (2.17,2.69)	1.15 (0.95,1.41)	***<0.001***	2.11 (1.91,2.33)
Past use	1.19 (1.04,1.37)	1.14 (0.87,1.50)	0.98 (0.76,1.26)	0.97 (0.69,1.36)	1.17 (0.92,1.49)	0.56	1.08 (0.97,1.20)	1.11 (0.94,1.32)	0.75	1.09 (0.99,1.20)
Past, duration of										
<5 years	1.06 (0.86,1.31)	1.14 (0.75,1.72)	1.05 (0.73,1.52)	1.10 (0.70,1.73)	1.17 (0.84,1.64)	0.98	0.96 (0.82,1.13)	1.16 (0.92,1.47)	0.15	1.01 (0.88,1.17)
≥5 years	1.31 (1.10,1.56)	1.27 (0.89,1.80)	0.87 (0.61,1.24)	0.86 (0.54,1.38)	1.18 (0.86,1.63)	0.15	1.16 (1.01,1.33)	1.00 (0.78,1.28)	0.27	1.12 (0.98,1.28)
Past, time since										
<5 years	1.15 (0.96,1.39)	1.20 (0.84,1.72)	0.92 (0.65,1.29)	1.01 (0.66,1.54)	1.39 (1.04,1.86)	0.41	1.06 (0.91,1.23)	1.25 (1.01,1.56)	0.17	1.11 (0.97,1.27)
5-10 years	1.24 (0.95,1.61)	1.22 (0.73,2.06)	1.13 (0.71,1.81)	1.16 (0.62,2.17)	1.13 (0.70,1.83)	0.99	1.10 (0.88,1.36)	1.00 (0.69,1.44)	0.63	1.07 (0.88,1.31)
≥10 years	0.94 (0.64,1.37)	0.53 (0.20,1.45)	0.84 (0.40,1.72)	0.86 (0.31,2.35)	0.55 (0.22,1.37)	0.73	0.77 (0.56,1.07)	0.73 (0.41,1.30)	0.86	0.76 (0.56,1.03)
Overweight										
Current use	2.19 (1.82,2.62)	1.01 (0.69,1.49)	1.85 (1.33,2.57)	1.13 (0.64,1.98)	0.92 (0.61,1.37)	***<0.001***	1.97 (1.72,2.25)	1.01 (0.78,1.32)	***<0.001***	1.76 (1.55,2.00)
Current, duration										
<5 years	1.81 (1.31,2.51)	0.48 (0.19,1.19)	1.58 (0.88,2.83)	1.14 (0.48,2.68)	0.35 (0.14,0.88)	***<0.001***	1.64 (1.29,2.08)	0.69 (0.43,1.10)	***<0.001***	1.39 (1.11,1.75)
≥5 years	2.27 (1.76,2.92)	1.41 (0.88,2.26)	1.94 (1.25,3.01)	1.27 (0.60,2.69)	1.21 (0.73,2.00)	***0.06***	2.34 (1.92,2.84)	1.27 (0.90,1.80)	***<0.001***	2.08 (1.73,2.51)
Past use	1.34 (1.10,1.63)	0.97 (0.66,1.44)	0.73 (0.47,1.14)	0.87 (0.47,1.61)	0.93 (0.63,1.38)	***0.05***	1.03 (0.88,1.20)	0.80 (0.60,1.08)	0.12	0.98 (0.84,1.13)
Past, duration of										
<5 years	1.22 (0.89,1.67)	0.97 (0.54,1.74)	0.70 (0.35,1.40)	0.94 (0.41,2.19)	0.58 (0.29,1.16)	***0.24***	0.96 (0.76,1.22)	0.63 (0.40,1.00)	0.09	0.88 (0.70,1.10)
≥5 years	1.47 (1.11,1.93)	0.82 (0.46,1.48)	0.68 (0.36,1.27)	0.87 (0.37,2.03)	1.11 (0.66,1.85)	0.07	1.08 (0.86,1.35)	0.91 (0.60,1.37)	0.43	1.04 (0.84,1.28)
Past, time since										
<5 years	1.56 (1.17,2.09)	0.92 (0.51,1.65)	0.73 (0.39,1.37)	1.16 (0.55,2.46)	0.91 (0.53,1.59)	0.08	1.21 (0.96,1.54)	0.90 (0.59,1.37)	0.18	1.14 (0.91,1.43)
5-10 years	1.26 (0.83,1.91)	0.79 (0.34,1.83)	0.76 (0.33,1.75)	0.56 (0.14,2.32)	0.61 (0.24,1.53)	0.38	0.86 (0.61,1.22)	0.58 (0.29,1.17)	0.29	0.80 (0.58,1.11)
≥10 years	0.78 (0.45,1.37)	0.84 (0.33,2.15)	0.57 (0.18,1.83)	0.80 (0.19,3.37)	0.64 (0.23,1.81)	0.98	0.65 (0.42,1.02)	0.61 (0.27,1.36)	0.87	0.63 (0.42,0.97)
Obese										
Current use	1.50 (1.09,2.07)	0.63 (0.31,1.28)	1.85 (1.11,3.08)	0.40 (0.10,1.66)	1.20 (0.68,2.09)	***0.05***	1.60 (1.28,2.01)	0.96 (0.61,1.50)	***0.02***	1.48 (1.18,1.84)
Current, duration										
<5 years	1.10 (0.61,1.99)	0.41 (0.10,1.70)	1.29 (0.50,3.35)	<0.01 (<0.01, .)	1.20 (0.54,2.66)	0.72	1.42 (0.95,2.13)	1.13 (0.60,2.13)	0.47	1.35 (0.92,1.98)
≥5 years	1.60 (0.98,2.59)	0.92 (0.36,2.36)	2.14 (1.05,4.36)	0.75 (0.18,3.20)	0.81 (0.32,2.10)	0.33	1.64 (1.15,2.36)	0.74 (0.36,1.52)	***0.03***	1.44 (1.02,2.05)
Past use	1.61 (1.22,2.12)	1.06 (0.62,1.83)	1.08 (0.61,1.90)	0.98 (0.42,2.32)	0.89 (0.52,1.52)	0.17	1.40 (1.12,1.74)	0.77 (0.49,1.19)	***0.007***	1.27 (1.03,1.58)
Past, duration of										
<5 years	0.93 (0.57,1.52)	0.94 (0.42,2.08)	0.92 (0.39,2.17)	0.58 (0.14,2.44)	0.46 (0.18,1.18)	0.72	1.13 (0.80,1.60)	0.53 (0.27,1.05)	***0.03***	0.99 (0.71,1.38)
≥5 years	2.25 (1.51,3.35)	1.19 (0.53,2.68)	0.98 (0.41,2.32)	1.43 (0.50,4.12)	1.29 (0.63,2.61)	0.17	1.55 (1.10,2.18)	1.06 (0.58,1.94)	0.21	1.45 (1.04,2.01)
Past, time since										

© 2025 Le Cornet C et al. *JAMA Network Open*.

<5 years	1.78 (1.07,2.95)	0.42 (0.10,1.75)	0.61 (0.18,1.99)	1.73 (0.59,5.01)	1.18 (0.51,2.70)	0.15	1.12 (0.73,1.71)	1.27 (0.67,2.41)	0.70	1.15 (0.77,1.71)
5-10 years	1.63 (0.97,2.73)	0.83 (0.29,2.37)	0.93 (0.32,2.67)	0.48 (0.06,3.59)	0.58 (0.20,1.67)	0.21	1.29 (0.82,2.03)	0.45 (0.17,1.17)	***0.03***	1.10 (0.70,1.71)
≥10 years	1.42 (0.67,3.01)	1.61 (0.52,4.95)	2.06 (0.73,5.81)	0.80 (0.10,6.13)	0.47 (0.10,2.09)	0.47	1.84 (0.98,3.45)	0.48 (0.14,1.70)	***0.03***	1.50 (0.80,2.81)

The model was adjusted for reference age (age at diagnosis for cases, age at interview for controls), study, OC and ET use (never, former, current)

Phet: Heterogeneity by breast cancer subtypes (e.g. ER+ and ER- or across intrinsic-like subtypes) was evaluated by comparing models assuming the same association versus different associations with exogenous hormones.

Lean/normal (BMI 18.5-<25), overweight (BMI 25-<30), obese (BMI >=30)

Intrinsic-like subtype definitions: luminal A-like (HR-positive, ERBB2-negative, grade 1&2), luminal B-like (HR-positive, ERBB2-negative, grade 3), luminal B-ERBB2-like (HR-positive, ERBB2-positive, any grade), ERBB2-enriched-like (HR-negative, ERBB2-positive, any grade), and triple-negative (HR-negative, ERBB2-negative, any grade). ER=estrogen receptor; PR=progesterone receptor.

Studies excluded: ABCFS, AHS, BCEES, BCINIS, CTS, ESTHER, GESBC, KARMA, KBCP, LAABC, MASTOS, MCCS, MISS, NBHS, PLCO, SBCGS, SMC.

© 2025 Le Cornet C et al. *JAMA Network Open*.

eTable 11. Odds Ratios and 95% CIs of Associations Between Estrogen-Only Therapy (ET) Use and Breast Cancer Intrinsic-Like Subtypes, in Postmenopausal Women, Stratified by Body Mass Index (BMI) in a Restricted Set of Studies With at Least 10 Cases Reporting ET Use

	Luminal A-like	Luminal B-like	Luminal B-ERBB2-like	ERBB2-enriched-like	Triple-negative	Phet	ER+	ER-	Phet	Invasive breast cancer
Lean/normal										
Current use	1.16 (1.01,1.33)	0.89 (0.67,1.19)	0.96 (0.75,1.24)	0.58 (0.38,0.88)	0.72 (0.54,0.97)	***0.001***	1.13 (1.03,1.24)	0.84 (0.70,1.01)	***0.002***	1.08 (0.98,1.17)
Current, duration										
<5 years	0.72 (0.53,0.98)	0.66 (0.35,1.26)	1.02 (0.63,1.63)	0.31 (0.11,0.83)	0.69 (0.41,1.15)	0.28	0.95 (0.78,1.15)	0.79 (0.57,1.09)	0.30	0.91 (0.76,1.09)
≥5 years	1.28 (1.08,1.52)	0.88 (0.61,1.28)	0.93 (0.67,1.30)	0.66 (0.40,1.08)	0.72 (0.50,1.03)	***0.002***	1.16 (1.02,1.32)	0.81 (0.64,1.03)	***0.004***	1.09 (0.96,1.22)
Past use	1.00 (0.87,1.14)	0.87 (0.65,1.15)	0.89 (0.70,1.14)	0.84 (0.58,1.22)	1.08 (0.84,1.40)	0.61	0.99 (0.89,1.09)	1.03 (0.86,1.24)	0.64	1.00 (0.91,1.10)
Past, duration of										
<5 years	1.05 (0.86,1.27)	0.84 (0.55,1.29)	0.79 (0.54,1.16)	0.83 (0.50,1.40)	1.25 (0.90,1.72)	0.28	0.99 (0.85,1.15)	1.06 (0.83,1.36)	0.57	1.00 (0.87,1.16)
≥5 years	1.12 (0.92,1.37)	0.89 (0.58,1.35)	0.99 (0.70,1.41)	0.98 (0.59,1.63)	1.00 (0.68,1.48)	0.84	1.02 (0.88,1.20)	1.05 (0.80,1.38)	0.86	1.03 (0.89,1.19)
Past, time since										
<5 years	1.20 (0.97,1.48)	1.24 (0.82,1.86)	1.40 (1.00,1.96)	1.07 (0.64,1.80)	1.02 (0.68,1.53)	0.78	1.24 (1.04,1.47)	1.09 (0.82,1.46)	0.43	1.21 (1.03,1.42)
5-10 years	1.00 (0.77,1.30)	0.56 (0.28,1.09)	0.56 (0.32,1.00)	1.15 (0.63,2.09)	1.12 (0.71,1.76)	0.14	0.83 (0.66,1.04)	0.97 (0.68,1.39)	0.42	0.86 (0.70,1.05)
≥10 years	0.98 (0.75,1.27)	0.69 (0.37,1.28)	0.57 (0.32,1.04)	0.52 (0.21,1.27)	1.34 (0.86,2.07)	0.06	0.84 (0.67,1.06)	1.12 (0.79,1.59)	0.14	0.90 (0.72,1.11)
Overweight										
Current use	0.75 (0.62,0.90)	0.48 (0.32,0.71)	0.81 (0.57,1.15)	0.79 (0.47,1.34)	0.58 (0.40,0.84)	0.18	0.76 (0.67,0.87)	0.71 (0.56,0.91)	0.59	0.75 (0.66,0.85)
Current, duration										
<5 years	0.29 (0.17,0.50)	0.45 (0.21,0.98)	0.45 (0.20,1.05)	0.97 (0.45,2.08)	0.31 (0.14,0.67)	0.12	0.49 (0.37,0.66)	0.56 (0.36,0.86)	0.61	0.52 (0.40,0.67)
≥5 years	0.80 (0.62,1.03)	0.32 (0.17,0.60)	0.95 (0.62,1.47)	0.62 (0.31,1.26)	0.67 (0.43,1.04)	***0.05***	0.80 (0.67,0.96)	0.73 (0.54,0.99)	0.56	0.78 (0.66,0.92)
Past use	0.78 (0.65,0.95)	0.80 (0.57,1.13)	0.79 (0.55,1.13)	0.82 (0.47,1.44)	0.70 (0.48,1.03)	0.98	0.82 (0.72,0.94)	0.71 (0.54,0.94)	0.33	0.81 (0.71,0.92)
Past, duration of										
<5 years	0.85 (0.63,1.16)	1.12 (0.69,1.81)	1.14 (0.70,1.86)	0.84 (0.38,1.84)	1.00 (0.62,1.60)	0.76	0.95 (0.77,1.17)	0.81 (0.56,1.17)	0.42	0.91 (0.75,1.12)
≥5 years	0.79 (0.59,1.06)	0.61 (0.35,1.08)	0.59 (0.33,1.07)	0.76 (0.35,1.69)	0.40 (0.19,0.83)	0.43	0.76 (0.61,0.95)	0.62 (0.40,0.95)	0.35	0.73 (0.59,0.90)
Past, time since										
<5 years	0.68 (0.46,1.00)	0.90 (0.50,1.63)	0.54 (0.26,1.13)	0.60 (0.22,1.68)	0.44 (0.20,0.96)	0.62	0.87 (0.66,1.15)	0.63 (0.38,1.05)	0.24	0.82 (0.63,1.06)
5-10 years	0.91 (0.61,1.35)	0.82 (0.41,1.67)	0.90 (0.46,1.76)	1.04 (0.41,2.65)	0.89 (0.45,1.75)	0.99	0.86 (0.63,1.19)	0.89 (0.52,1.50)	0.93	0.86 (0.64,1.16)
≥10 years	0.86 (0.59,1.27)	0.80 (0.41,1.57)	1.05 (0.56,1.96)	0.82 (0.29,2.31)	1.00 (0.53,1.89)	0.96	0.86 (0.63,1.18)	0.95 (0.57,1.58)	0.72	0.87 (0.64,1.17)
Obese										
Current use	0.66 (0.50,0.86)	0.43 (0.25,0.74)	0.42 (0.24,0.77)	0.56 (0.25,1.25)	0.65 (0.43,0.99)	0.47	0.67 (0.55,0.81)	0.60 (0.43,0.84)	0.54	0.65 (0.54,0.78)
Current, duration										
<5 years	0.48 (0.27,0.86)	0.18 (0.04,0.75)	<0.01 (<0.01,****)	0.23 (0.03,1.68)	0.56 (0.27,1.17)	0.65	0.49 (0.33,0.74)	0.63 (0.36,1.11)	0.41	0.53 (0.37,0.76)
≥5 years	0.60 (0.41,0.88)	0.29 (0.13,0.67)	0.47 (0.22,0.98)	0.73 (0.31,1.73)	0.61 (0.36,1.03)	0.50	0.60 (0.46,0.78)	0.49 (0.32,0.77)	0.41	0.57 (0.44,0.74)
Past use	0.84 (0.67,1.07)	0.77 (0.51,1.18)	0.59 (0.37,0.94)	1.07 (0.59,1.94)	0.57 (0.38,0.86)	0.21	0.70 (0.59,0.84)	0.56 (0.40,0.77)	0.15	0.67 (0.57,0.80)
Past, duration of										
<5 years	0.76 (0.53,1.10)	0.75 (0.41,1.35)	0.53 (0.26,1.08)	0.77 (0.32,1.85)	0.42 (0.23,0.76)	0.36	0.65 (0.49,0.86)	0.41 (0.25,0.65)	***0.05***	0.59 (0.45,0.76)
≥5 years	1.14 (0.80,1.64)	0.85 (0.44,1.64)	0.73 (0.37,1.46)	1.49 (0.68,3.28)	0.86 (0.49,1.51)	0.51	0.85 (0.63,1.15)	0.82 (0.51,1.31)	0.89	0.83 (0.62,1.11)
Past, time since										
<5 years	1.55 (0.95,2.52)	0.82 (0.32,2.10)	0.69 (0.24,1.96)	1.77 (0.67,4.68)	1.16 (0.57,2.33)	0.40	1.21 (0.79,1.84)	1.21 (0.68,2.18)	0.98	1.21 (0.81,1.80)

© 2025 Le Cornet C et al. *JAMA Network Open*.

5-10 years	0.81 (0.48,1.35)	0.86 (0.38,1.95)	0.57 (0.22,1.48)	1.07 (0.37,3.10)	0.37 (0.14,0.96)	0.50	0.77 (0.51,1.17)	0.42 (0.20,0.89)	0.11	0.69 (0.46,1.02)
≥10 years	0.82 (0.54,1.25)	0.90 (0.46,1.76)	0.75 (0.37,1.53)	0.71 (0.25,2.07)	0.48 (0.25,0.90)	0.54	0.79 (0.55,1.13)	0.43 (0.24,0.77)	***0.03***	0.68 (0.47,0.97)

The model was adjusted for reference age (age at diagnosis for cases, age at interview for controls), study, OC and EPT use (never, former, current).

Phet: Heterogeneity by breast cancer subtypes (e.g. ER+ and ER- or across intrinsic-like subtypes) was evaluated by comparing models assuming the same association versus different associations with exogenous hormones.

Lean/normal (BMI 18.5-<25), overweight (BMI 25-<30), obese (BMI >=30).

Intrinsic-like subtype definitions: luminal A-like (HR-positive, ERBB2-negative, grade 1&2), luminal B-like (HR-positive, ERBB2-negative, grade 3), luminal B-ERBB2-like (HR-positive, ERBB2-positive, any grade), ERBB2-enriched-like (HR-negative, ERBB2-positive, any grade), and triple-negative (HR-negative, ERBB2-negative, any grade). ER=estrogen receptor; PR=progesterone receptor.

Studies excluded: ABCFS, AHS, BCEES, BCINIS, CTS, ESTHER, GESBC, KARMA, KBCP, LAABC, MASTOS, MCCS, MISS, NBHS, PLCO, SBCGS, SMC.

© 2025 Le Cornet C et al. *JAMA Network Open*.

eTable 12. Odds Ratios and 95% CIs of Associations Between Oral Contraceptive (OC) Use and Breast Cancer Subtypes, in Premenopausal Women Overall in a Restricted Set of Studies With at Least 10 Cases Reporting OC Use

	Luminal A-like	Luminal B-like	Luminal B-ERBB2-like	ERBB2-enriched-like	Triple-negative	Phet	ER+	ER-	Phet	Invasive breast cancer
Current use	1.19 (0.98,1.45)	1.37 (1.00,1.88)	0.95 (0.71,1.27)	0.89 (0.57,1.38)	0.95 (0.73,1.24)	0.21	0.96 (0.85,1.09)	0.84 (0.70,1.01)	0.17	0.92 (0.82,1.04)
Current, duration										
<5 years	0.69 (0.42,1.14)	1.22 (0.61,2.42)	0.69 (0.31,1.51)	0.39 (0.09,1.64)	0.64 (0.33,1.24)	0.48	0.80 (0.59,1.10)	0.69 (0.45,1.06)	0.51	0.78 (0.59,1.04)
≥5 years	1.29 (1.04,1.61)	1.44 (1.02,2.03)	1.04 (0.76,1.42)	0.95 (0.59,1.52)	0.98 (0.74,1.30)	0.24	1.00 (0.87,1.14)	0.85 (0.70,1.04)	0.14	0.95 (0.84,1.08)
Past use	0.87 (0.77,0.99)	0.99 (0.80,1.24)	0.92 (0.76,1.12)	0.99 (0.74,1.33)	1.05 (0.88,1.26)	0.37	0.95 (0.87,1.04)	1.08 (0.95,1.22)	0.06	0.98 (0.91,1.06)
Past, duration of										
<5 years	0.85 (0.73,0.99)	1.04 (0.80,1.34)	0.96 (0.76,1.21)	0.80 (0.56,1.14)	0.93 (0.75,1.15)	0.58	0.93 (0.84,1.03)	0.95 (0.82,1.10)	0.78	0.93 (0.85,1.02)
≥5 years	0.84 (0.73,0.98)	0.94 (0.73,1.21)	0.92 (0.74,1.15)	1.10 (0.80,1.51)	1.10 (0.90,1.34)	0.11	0.97 (0.88,1.07)	1.15 (1.00,1.31)	***0.02***	1.01 (0.93,1.11)
Past, time since										
<5 years	1.03 (0.81,1.31)	1.25 (0.86,1.83)	1.25 (0.90,1.72)	1.97 (1.30,2.98)	1.50 (1.14,1.97)	***0.03***	1.27 (1.11,1.45)	1.53 (1.28,1.82)	***0.04***	1.36 (1.20,1.54)
>5-10 years	1.06 (0.84,1.32)	1.10 (0.76,1.60)	1.21 (0.89,1.66)	1.07 (0.67,1.71)	1.42 (1.09,1.86)	0.36	1.12 (0.97,1.29)	1.27 (1.06,1.53)	0.18	1.16 (1.02,1.32)
≥10 years	0.80 (0.70,0.92)	0.91 (0.72,1.14)	0.83 (0.68,1.03)	0.81 (0.59,1.12)	0.90 (0.74,1.09)	0.76	0.87 (0.79,0.95)	0.94 (0.82,1.07)	0.29	0.88 (0.81,0.96)

The model was adjusted for reference age (age at diagnosis for cases, age at interview for controls), study, and BMI (lean, overweight, obese).

Phet: Heterogeneity by breast cancer subtypes (e.g. ER+ and ER- or across intrinsic-like subtypes) was evaluated by comparing models assuming the same association versus different associations with exogenous hormones.

Intrinsic-like subtype definitions: luminal A-like (HR-positive, ERBB2-negative, grade 1&2), luminal B-like (HR-positive, ERBB2-negative, grade 3), luminal B-ERBB2-like (HR-positive, ERBB2-positive, any grade), ERBB2-enriched-like (HR-negative, ERBB2-positive, any grade), and triple-negative (HR-negative, ERBB2-negative, any grade). ER=estrogen receptor; PR=progesterone receptor.

Studies excluded: AHS, CPSII, CTS, ESTHER, KARMA, KBCP, LAABC, MASTOS, MEC, NHS, PLCO, PROCAS, SASBAC, SBCGS, SMC.

© 2025 Le Cornet C et al. *JAMA Network Open*.

eTable 13. Associations Between Estrogen-Progestin Therapy (EPT) Use and Breast Cancer Intrinsic-Like Subtypes, in Postmenopausal Women, Stratified by Body Mass Index (BMI) and Study Design

	Luminal A-like	Luminal B-like	Luminal B-ERBB2-like	ERBB2-enriched-like	Triple-negative	Phet	ER+	ER-	Phet	Invasive breast cancer
Lean/normal										
Current use										
Cohort	2.94 (2.35,3.69)	2.53 (1.62,3.95)	1.95 (1.22,3.13)	2.48 (1.05,5.87)	0.94 (0.42,2.10)	***0.01***	2.57 (2.22,2.99)	1.17 (0.77,1.76)	***<0.001***	2.41 (2.08,2.79)
Case-control	2.35 (2.08,2.66)	1.17 (0.89,1.55)	1.91 (1.54,2.38)	0.63 (0.43,0.93)	1.02 (0.79,1.32)	***<0.001***	2.00 (1.81,2.20)	1.09 (0.91,1.29)	***<0.001***	1.77 (1.61,1.94)
Past use										
Cohort	1.34 (0.97,1.86)	1.61 (0.90,2.87)	1.44 (0.78,2.65)	1.92 (0.68,5.40)	1.22 (0.55,2.72)	0.96	1.14 (0.92,1.41)	1.24 (0.79,1.96)	0.79	1.15 (0.94,1.42)
Case-control	1.13 (0.97,1.31)	0.99 (0.73,1.35)	0.95 (0.72,1.25)	0.87 (0.61,1.25)	1.12 (0.87,1.44)	0.51	1.05 (0.93,1.18)	1.04 (0.86,1.26)	0.95	1.04 (0.94,1.16)
Overweight										
Current use										
Cohort	3.46 (2.63,4.56)	1.12 (0.55,2.25)	3.05 (1.69,5.51)	2.43 (0.91,6.48)	2.11 (0.97,4.58)	***0.03***	2.55 (2.10,3.10)	1.25 (0.77,2.03)	***0.003***	2.37 (1.95,2.86)
Case-control	1.53 (1.20,1.96)	0.87 (0.54,1.39)	1.37 (0.91,2.05)	0.82 (0.40,1.67)	0.73 (0.45,1.18)	***0.005***	1.55 (1.29,1.87)	0.90 (0.65,1.24)	***0.0005***	1.38 (1.16,1.64)
Past use										
Cohort	1.18 (0.82,1.70)	1.27 (0.65,2.49)	0.91 (0.35,2.35)	0.82 (0.18,3.70)	2.02 (0.95,4.30)	0.64	0.92 (0.71,1.20)	0.89 (0.51,1.56)	0.85	0.92 (0.71,1.18)
Case-control	1.45 (1.14,1.84)	0.89 (0.55,1.43)	0.67 (0.41,1.11)	0.93 (0.48,1.83)	0.82 (0.51,1.31)	***0.008***	1.09 (0.90,1.31)	0.75 (0.53,1.07)	***0.04***	1.01 (0.84,1.21)
Obese										
Current use										
Cohort	2.99 (1.98,4.53)	1.90 (0.82,4.43)	3.75 (1.85,7.57)	<0.01 (<0.01,****)	3.72 (1.32,10.5)	0.78	2.60 (1.94,3.49)	0.96 (0.43,2.16)	***0.01***	2.36 (1.77,3.15)
Case-control	0.66 (0.39,1.12)	0.22 (0.05,0.92)	0.92 (0.43,1.98)	0.41 (0.10,1.72)	0.78 (0.40,1.54)	0.44	0.86 (0.59,1.25)	0.81 (0.47,1.40)	0.67	0.84 (0.59,1.20)
Past use										
Cohort	2.39 (1.61,3.55)	1.25 (0.51,3.08)	1.27 (0.48,3.39)	0.97 (0.11,8.33)	3.68 (1.58,8.60)	0.27	1.59 (1.17,2.17)	1.07 (0.54,2.11)	0.20	1.51 (1.12,2.04)
Case-control	1.24 (0.84,1.83)	1.00 (0.50,2.00)	0.99 (0.50,1.98)	0.93 (0.36,2.39)	0.44 (0.20,0.99)	0.18	1.24 (0.90,1.69)	0.60 (0.34,1.07)	***0.01***	1.09 (0.80,1.48)

The model was adjusted for reference age (age at diagnosis for cases, age at interview for controls), study, OC and ET use (never, former, current)

Phet: Heterogeneity by breast cancer subtypes (e.g. ER+ and ER- or across intrinsic-like subtypes) was evaluated by comparing models assuming the same association versus different associations with exogenous hormones.

Lean/normal (BMI 18.5-<25), overweight (BMI 25-<30), obese (BMI >=30)

Intrinsic-like subtype definitions: luminal A-like (HR-positive, ERBB2-negative, grade 1&2), luminal B-like (HR-positive, ERBB2-negative, grade 3), luminal B-ERBB2-like (HR-positive, ERBB2-positive, any grade), ERBB2-enriched-like (HR-negative, ERBB2-positive, any grade), and triple-negative (HR-negative, ERBB2-negative, any grade). ER=estrogen receptor; PR=progesterone receptor.

© 2025 Le Cornet C et al. *JAMA Network Open*.

eTable 14. Associations Between Estrogen-Only Therapy (ET) Use and Breast Cancer Intrinsic-Like Subtypes, in Postmenopausal Women, Stratified by Body Mass Index (BMI) and Study Design

	Luminal A-like	Luminal B-like	Luminal B-ERBB2-like	ERBB2-enriched-like	Triple-negative	Phet	ER+	ER-	Phet	Invasive breast cancer
Lean/normal										
Current use										
Cohort	1.25 (0.99,1.59)	1.24 (0.78,1.98)	1.56 (1.01,2.43)	0.54 (0.16,1.86)	0.78 (0.37,1.64)	0.42	1.34 (1.16,1.56)	0.93 (0.63,1.37)	***0.05***	1.29 (1.12,1.50)
Case-control	1.16 (0.98,1.36)	0.74 (0.51,1.08)	0.82 (0.59,1.12)	0.62 (0.39,0.98)	0.73 (0.53,1.00)	***0.002***	1.00 (0.88,1.13)	0.78 (0.63,0.97)	***0.03***	0.95 (0.84,1.07)
Past use										
Cohort	0.65 (0.49,0.86)	0.72 (0.41,1.28)	0.88 (0.51,1.52)	0.76 (0.22,2.65)	0.61 (0.25,1.49)	0.93	0.80 (0.67,0.96)	0.59 (0.35,0.99)	0.25	0.79 (0.66,0.94)
Case-control	1.12 (0.97,1.30)	0.88 (0.64,1.22)	0.92 (0.70,1.21)	0.90 (0.61,1.33)	1.20 (0.92,1.56)	0.24	1.08 (0.95,1.22)	1.16 (0.95,1.40)	0.55	1.10 (0.98,1.23)
Overweight										
Current use										
Cohort	0.96 (0.72,1.27)	0.86 (0.49,1.50)	1.21 (0.67,2.20)	1.14 (0.41,3.19)	1.57 (0.82,3.01)	0.51	0.91 (0.76,1.09)	0.91 (0.60,1.38)	0.88	0.91 (0.77,1.08)
Case-control	0.57 (0.44,0.75)	0.32 (0.18,0.56)	0.67 (0.43,1.04)	0.68 (0.37,1.26)	0.38 (0.24,0.62)	0.11	0.61 (0.50,0.73)	0.57 (0.42,0.78)	0.66	0.59 (0.50,0.71)
Past use										
Cohort	0.69 (0.51,0.94)	0.68 (0.36,1.27)	0.75 (0.37,1.53)	0.29 (0.04,2.27)	0.59 (0.22,1.57)	0.92	0.68 (0.56,0.83)	0.32 (0.16,0.61)	***0.02***	0.65 (0.54,0.79)
Case-control	0.85 (0.67,1.08)	0.87 (0.58,1.30)	0.81 (0.54,1.22)	1.13 (0.65,1.98)	0.79 (0.52,1.19)	0.87	0.94 (0.78,1.12)	0.87 (0.65,1.18)	0.59	0.93 (0.78,1.10)
Obese										
Current use										
Cohort	1.06 (0.73,1.52)	0.80 (0.38,1.71)	0.77 (0.36,1.65)	0.50 (0.06,4.23)	1.14 (0.40,3.20)	0.85	0.88 (0.69,1.12)	0.57 (0.30,1.07)	0.15	0.84 (0.66,1.06)
Case-control	0.40 (0.27,0.61)	0.25 (0.11,0.57)	0.19 (0.07,0.52)	0.52 (0.22,1.23)	0.53 (0.33,0.86)	0.23	0.45 (0.33,0.61)	0.52 (0.35,0.78)	0.53	0.47 (0.35,0.62)
Past use										
Cohort	0.65 (0.44,0.96)	0.77 (0.37,1.59)	0.26 (0.08,0.85)	2.84 (0.78,10.4)	0.95 (0.34,2.66)	0.11	0.48 (0.36,0.63)	0.51 (0.27,0.97)	0.95	0.48 (0.37,0.63)
Case-control	1.05 (0.78,1.40)	0.85 (0.51,1.40)	0.81 (0.48,1.36)	0.88 (0.45,1.73)	0.66 (0.42,1.02)	0.24	0.95 (0.74,1.21)	0.63 (0.43,0.92)	***0.02***	0.87 (0.68,1.10)

The model was adjusted for reference age (age at diagnosis for cases, age at interview for controls), study, OC and EPT use (never, former, current).

Phet: Heterogeneity by breast cancer subtypes (e.g. ER+ and ER- or across intrinsic-like subtypes) was evaluated by comparing models assuming the same association versus different associations with exogenous hormones.

Lean/normal (BMI 18.5-<25), overweight (BMI 25-<30), obese (BMI >=30).

Intrinsic-like subtype definitions: luminal A-like (HR-positive, ERBB2-negative, grade 1&2), luminal B-like (HR-positive, ERBB2-negative, grade 3), luminal B-ERBB2-like (HR-positive, ERBB2-positive, any grade), ERBB2-enriched-like (HR-negative, ERBB2-positive, any grade), and triple-negative (HR-negative, ERBB2-negative, any grade). ER=estrogen receptor; PR=progesterone receptor.

© 2025 Le Cornet C et al. *JAMA Network Open*.

eTable 15. Associations Between Oral Contraceptive (OC) Use and Breast Cancer Intrinsic-Like Subtypes, in Premenopausal Women Overall, Stratified by Study Design

	Luminal A-like	Luminal B-like	Luminal B-ERBB2-like	ERBB2-enriched-like	Triple-negative	Phet	ER+	ER-	Phet	Invasive breast cancer
Current use										
Cohort	1.85 (1.32,2.59)	1.69 (0.91,3.14)	1.23 (0.66,2.29)	1.61 (0.42,6.22)	3.46 (1.85,6.48)	0.13	1.30 (1.04,1.62)	1.19 (0.78,1.82)	0.89	1.27 (1.03,1.57)
Case-control	0.93 (0.73,1.18)	1.14 (0.79,1.64)	0.92 (0.66,1.29)	0.80 (0.50,1.29)	0.76 (0.57,1.02)	0.08	0.96 (0.83,1.12)	0.92 (0.76,1.12)	0.31	0.95 (0.82,1.08)
Past use										
Cohort	1.06 (0.85,1.33)	1.18 (0.80,1.76)	1.20 (0.84,1.72)	2.08 (0.97,4.50)	1.53 (0.94,2.49)	0.21	0.98 (0.86,1.12)	1.04 (0.80,1.34)	0.32	0.99 (0.87,1.12)
Case-control	0.83 (0.71,0.96)	0.94 (0.73,1.22)	1.00 (0.79,1.26)	0.98 (0.71,1.34)	1.09 (0.89,1.33)	0.64	1.14 (1.03,1.26)	1.32 (1.16,1.51)	0.16	1.19 (1.08,1.31)

The model was adjusted for reference age (age at diagnosis for cases, age at interview for controls), study, and BMI (lean, overweight, obese).

Phet: Heterogeneity by breast cancer subtypes (e.g. ER+ and ER- or across intrinsic-like subtypes) was evaluated by comparing models assuming the same association versus different associations with exogenous hormones.

Intrinsic-like subtype definitions: luminal A-like (HR-positive, ERBB2-negative, grade 1&2), luminal B-like (HR-positive, ERBB2-negative, grade 3), luminal B-ERBB2-like (HR-positive, ERBB2-positive, any grade), ERBB2-enriched-like (HR-negative, ERBB2-positive, any grade), and triple-negative (HR-negative, ERBB2-negative, any grade). ER=estrogen receptor; PR=progesterone receptor.

© 2025 Le Cornet C et al. *JAMA Network Open*.

eTable 16. Heterogeneity Between Studies Set Up Before and After 2000 for the Associations Between Estrogen-Progestin Therapy (EPT) Use and Breast Cancer Intrinsic-Like Subtypes, in Postmenopausal Women, Stratified by Body Mass Index (BMI)

	Luminal A-like	Luminal B-like	Luminal B-ERBB2-like	ERBB2-enriched-like	Triple-negative	Phet	ER+	ER-	Phet	Invasive breast cancer
Lean/normal										
Current use										
Before	2.52 [2.00, 3.17]	2.29 [1.45, 3.62]	1.95 [1.22, 3.13]	1.46 [0.57, 3.69]	0.90 [0.41, 1.97]	0.06	2.17 [1.88, 2.50]	1.32 [0.93, 1.88]	***0.02***	2.04 [1.78, 2.35]
After	2.67 [2.35, 3.03]	1.35 [1.01, 1.79]	1.84 [1.47, 2.32]	0.68 [0.46, 1.02]	1.28 [0.98, 1.67]	***<0.001***	2.27 [2.04, 2.53]	1.2 [0.99, 1.46]	***<0.001***	2.00 [1.81, 2.21]
Past use										
Before	1.23 [0.85, 1.77]	1.28 [0.64, 2.56]	1.45 [0.75, 2.80]	1.83 [0.60, 5.60]	1.33 [0.57, 3.08]	0.98	1.15 [0.92, 1.45]	1.54 [0.98, 2.41]	0.17	1.19 [0.96, 1.48]
After	1.16 [1.00, 1.36]	1.04 [0.76, 1.43]	0.92 [0.69, 1.23]	0.94 [0.65, 1.34]	1.27 [0.97, 1.67]	0.36	1.08 [0.95, 1.22]	1.08 [0.88, 1.32]	0.95	1.08 [0.96, 1.21]
Overweight										
Current use										
Before	3.08 (2.32,4.08)	1.36 (0.70,2.64)	3.36 (1.85,6.11)	2.61 (1.00,6.79)	1.25 (0.51,3.07)	***0.04***	2.50 (2.06,3.04)	1.36 (0.87,2.13)	***0.02***	2.31 (1.92,2.78)
After	2.30 (1.77,2.99)	1.13 (0.67,1.89)	1.72 (1.11,2.65)	1.38 (0.66,2.89)	1.13 (0.66,1.95)	***0.01***	1.99 (1.61,2.46)	1.22 (0.83,1.80)	***0.009***	1.81 (1.48,2.21)
Past use										
Before	1.14 (0.76,1.71)	1.29 (0.62,2.69)	1.20 (0.46,3.10)	1.06 (0.23,4.82)	1.90 (0.83,4.32)	0.92	0.92 (0.69,1.23)	0.99 (0.55,1.77)	0.85	0.93 (0.71,1.21)
After	1.46 (1.13,1.89)	0.83 (0.49,1.41)	0.63 (0.36,1.09)	1.17 (0.57,2.41)	1.00 (0.61,1.67)	***0.03***	1.10 (0.90,1.36)	0.96 (0.65,1.41)	0.41	1.08 (0.89,1.30)
Obese										
Current use										
Before	2.36 (2.34,2.38)	1.88 (1.85,1.91)	3.15 (3.10,3.20)	0.28 (0.26,0.30)	2.23 (2.18,2.28)	0.91	2.34 (1.73,3.16)	1.14 (0.51,2.57)	0.06	2.17 (1.62,2.91)
After	1.36 (0.74,2.53)	0.59 (0.14,2.53)	1.66 (0.70,3.91)	0.47 (0.06,3.59)	2.04 (0.81,5.16)	0.54	1.36 (0.85,2.17)	1.32 (0.60,2.93)	0.82	1.34 (0.86,2.08)
Past use										
Before	2.52 (2.50,2.55)	1.32 (1.29,1.34)	0.99 (0.96,1.01)	1.07 (1.02,1.12)	3.84 (3.77,3.90)	0.24	1.72 (1.20,2.47)	1.43 (0.67,3.06)	0.58	1.68 (1.19,2.37)
After	1.39 (0.90,2.14)	1.34 (0.58,3.10)	0.55 (0.19,1.59)	1.35 (0.45,3.99)	0.74 (0.26,2.12)	0.40	1.19 (0.84,1.70)	0.87 (0.42,1.81)	0.37	1.13 (0.81,1.59)

The model was adjusted for reference age (age at diagnosis for cases, age at interview for controls), study, OC and ET use (never, former, current)

Phet: Heterogeneity by breast cancer subtypes (e.g. ER+ and ER- or across intrinsic-like subtypes) was evaluated by comparing models assuming the same association versus different associations with exogenous hormones.

Lean/normal (BMI 18.5-<25), overweight (BMI 25-<30), obese (BMI >=30)

Intrinsic-like subtype definitions: luminal A-like (HR-positive, ERBB2-negative, grade 1&2), luminal B-like (HR-positive, ERBB2-negative, grade 3), luminal B-ERBB2-like (HR-positive, ERBB2-positive, any grade), ERBB2-enriched-like (HR-negative, ERBB2-positive, any grade), and triple-negative (HR-negative, ERBB2-negative, any grade). ER=estrogen receptor; PR=progesterone receptor.

© 2025 Le Cornet C et al. *JAMA Network Open*.

eTable 17. Heterogeneity Between Studies Set Up Before and After 2000 for the Associations Between Estrogen-Only Therapy (ET) Use and Breast Cancer Intrinsic-Like Subtypes, in Postmenopausal Women, Stratified by Body Mass Index (BMI)

	Luminal A-like	Luminal B-like	Luminal B-ERBB2-like	ERBB2-enriched-like	Triple-negative	Phet	ER+	ER-	Phet	Invasive breast cancer
Lean/normal										
Current use										
Before	1.13 (0.89,1.45)	1.24 (0.77,2.01)	1.56 (0.99,2.46)	0.54 (0.16,1.87)	0.89 (0.43,1.84)	0.39	1.29 (1.12,1.49)	1.32 (0.95,1.84)	0.98	1.28 (1.12,1.47)
After	1.44 (1.21,1.71)	0.85 (0.58,1.26)	0.82 (0.58,1.17)	0.76 (0.47,1.23)	0.73 (0.49,1.09)	*<0.001*	1.26 (1.09,1.46)	0.86 (0.66,1.13)	*0.007*	1.16 (1.02,1.33)
Past use										
Before	0.64 (0.48,0.86)	0.66 (0.36,1.21)	1.03 (0.60,1.77)	1.15 (0.38,3.54)	1.18 (0.59,2.35)	0.20	0.89 (0.74,1.07)	1.27 (0.83,1.93)	0.08	0.93 (0.78,1.11)
After	1.19 (1.02,1.39)	0.93 (0.66,1.30)	0.88 (0.65,1.18)	0.92 (0.60,1.38)	0.99 (0.72,1.36)	0.25	1.10 (0.97,1.25)	1.01 (0.81,1.27)	0.47	1.08 (0.96,1.22)
Overweight										
Current use										
Before	1.02 (0.77,1.36)	0.86 (0.48,1.55)	1.35 (0.72,2.52)	1.34 (0.47,3.79)	1.49 (0.77,2.89)	0.66	0.97 (0.81,1.17)	1.14 (0.77,1.69)	0.58	0.99 (0.84,1.18)
After	0.99 (0.72,1.35)	0.58 (0.31,1.09)	0.91 (0.54,1.52)	0.57 (0.20,1.60)	0.68 (0.34,1.38)	0.45	0.86 (0.66,1.10)	0.71 (0.43,1.16)	0.44	0.82 (0.65,1.04)
Past use										
Before	0.72 (0.53,0.98)	0.75 (0.40,1.38)	0.80 (0.38,1.69)	0.91 (0.25,3.23)	0.52 (0.19,1.38)	0.95	0.75 (0.61,0.92)	0.54 (0.30,0.99)	0.30	0.74 (0.60,0.90)
After	0.81 (0.60,1.07)	0.58 (0.32,1.04)	0.81 (0.50,1.29)	0.80 (0.36,1.78)	0.83 (0.47,1.48)	0.88	0.88 (0.71,1.09)	0.84 (0.55,1.27)	0.86	0.87 (0.71,1.07)
Obese										
Current use										
Before	0.85 (0.84,0.85)	0.88 (0.86,0.89)	0.73 (0.72,0.74)	0.51 (0.49,0.54)	0.70 (0.68,0.72)	0.97	0.88 (0.67,1.14)	0.76 (0.39,1.49)	0.60	0.86 (0.67,1.11)
After	0.98 (0.56,1.71)	0.20 (0.03,1.49)	0.17 (0.02,1.27)	0.34 (0.04,2.57)	1.51 (0.61,3.76)	0.14	0.73 (0.45,1.18)	0.95 (0.44,2.08)	0.64	0.77 (0.50,1.19)
Past use										
Before	0.58 (0.58,0.59)	0.90 (0.88,0.91)	0.31 (0.30,0.31)	1.91 (1.86,1.95)	1.09 (1.07,1.11)	0.13	0.58 (0.44,0.78)	0.80 (0.41,1.57)	0.42	0.61 (0.46,0.81)
After	0.96 (0.62,1.49)	0.83 (0.36,1.90)	0.34 (0.12,0.97)	0.36 (0.08,1.55)	0.51 (0.18,1.47)	0.25	0.74 (0.52,1.07)	0.45 (0.20,1.02)	0.21	0.69 (0.49,0.97)

The model was adjusted for reference age (age at diagnosis for cases, age at interview for controls), study, OC and EPT use (never, former, current).
Phet: Heterogeneity by breast cancer subtypes (e.g. ER+ and ER- or across intrinsic-like subtypes) was evaluated by comparing models assuming the same association versus different associations with exogenous hormones.
Lean/normal (BMI 18.5-<25), overweight (BMI 25-<30), obese (BMI >=30).
Intrinsic-like subtype definitions: luminal A-like (HR-positive, ERBB2-negative, grade 1&2), luminal B-like (HR-positive, ERBB2-negative, grade 3), luminal B-ERBB2-like (HR-positive, ERBB2-positive, any grade), ERBB2-enriched-like (HR-negative, ERBB2-positive, any grade), and triple-negative (HR-negative, ERBB2-negative, any grade). ER=estrogen receptor; PR=progesterone receptor.

© 2025 Le Cornet C et al. *JAMA Network Open*.

eTable 18. Number of Women Included in the Associations Between Estrogen-Progestin Therapy (EPT) Use and Breast Cancer Intrinsic-Like Subtypes, in Postmenopausal Women, Stratified by Body Mass Index (BMI) (Corresponds to Results in eTables 3 and 7)

	Luminal A-like	Luminal B-like	Luminal B-ERBB2-like	ERBB2-enriched-like	Triple-negative	ER+	ER-	Invasive breast cancer	Controls
Lean/normal	4396	838	979	485	1003	10698	2465	13163	23345
Never	2276	473	585	329	610	5486	1502	6988	14119
Current use	802	112	178	41	93	1667	227	1894	2055
Current, duration of use									
<5 years	155	19	44	11	21	339	66	405	524
≥5 years	517	68	101	24	64	889	137	1026	1052
Past use	334	68	78	43	90	652	183	835	1554
Past, duration of use									
<5 years	119	26	34	22	42	232	92	324	633
≥5 years	188	39	37	20	46	332	81	413	763
Past, time since last use									
<5 years	163	37	40	26	60	288	109	397	755
5-10 years	75	16	20	11	19	130	34	164	300
≥10 years	35	4	8	4	5	54	13	67	151
Overweight	3018	673	611	328	662	7514	1629	9143	15046
Never	1849	434	405	214	440	4408	1054	5462	9512
Current use	231	34	52	18	30	563	76	639	672
Current, duration of use									
<5 years	59	5	14	6	5	132	21	153	200
≥5 years	111	22	27	8	19	248	42	290	251
Past use	159	32	23	12	31	304	55	359	615
Past, duration of use									
<5 years	57	13	9	6	9	114	22	136	252
≥5 years	81	13	11	6	18	141	28	169	272
Past, time since last use									
<5 years	71	13	11	8	15	125	27	152	230
5-10 years	33	6	6	2	5	54	9	63	113
≥10 years	17	5	3	2	4	32	7	39	67
Obese	1939	469	418	202	549	4625	1077	5702	7446
Never	1322	358	284	152	416	3003	785	3788	4881
Current use	61	9	20	2	16	174	25	199	225
Current, duration of use									
<5 years	16	2	5	0	8	48	13	61	73
≥5 years	27	5	10	2	5	66	9	75	79
Past use	99	16	15	6	17	189	26	215	236
Past, duration of use									
<5 years	25	7	6	2	5	69	10	79	101
≥5 years	51	7	6	4	10	78	14	92	88

© 2025 Le Cornet C et al. *JAMA Network Open*.

Past, time since last use									
<5 years	25	2	3	4	7	39	13	52	65
5-10 years	29	4	4	1	4	46	5	51	47
≥10 years	15	4	5	1	2	36	3	39	18

© 2025 Le Cornet C et al. *JAMA Network Open*.

eTable 19. Number of Women Included in the Associations Between Menopausal Hormonal Therapy (MHT) Use and Breast Cancer Subtypes, in Postmenopausal Women Overall (Corresponds to Results in eTable 4)

	Luminal A-like	Luminal B-like	Luminal B-ERBB2-like	ERBB2-enriched-like	Triple-negative	ER+	ER-	Invasive breast cancer	Controls
EPT	9815	2079	2192	1060	2337	24751	5617	30368	49405
Never	5655	1304	1360	714	1512	13416	3466	16882	29497
Current use	1126	159	262	65	144	2465	343	2808	3073
Current, duration of use									
<5 years	242	26	65	18	34	535	104	639	829
≥5 years	670	99	143	36	91	1230	196	1426	1450
Past use	612	119	124	62	140	1186	270	1456	2489
Past, duration of use									
<5 years	205	47	54	31	56	428	126	554	1018
≥5 years	333	60	57	30	75	571	126	697	1164
Past, time since last use									
<5 years	270	52	58	38	83	471	151	622	1085
5-10 years	142	28	32	15	28	239	51	290	488
≥10 years	67	13	18	7	11	125	23	148	241
ET	9815	2079	2192	1060	2337	24751	5617	30368	49405
Never	6078	1332	1421	719	1519	13691	3471	17162	28666
Current use	627	111	153	52	128	1661	306	1967	3374
Current, duration of use									
<5 years	88	22	29	13	34	283	90	373	781
≥5 years	349	51	90	35	82	804	182	986	1533
Past use	709	138	158	68	157	1568	301	1869	2804
Past, duration of use									
<5 years	280	61	67	31	92	593	157	750	1094
≥5 years	304	54	68	34	59	564	127	691	954
Past, time since last use									
<5 years	212	47	59	27	52	393	104	497	685
5-10 years	152	25	34	22	40	262	68	330	492
≥10 years	183	37	38	15	53	309	85	394	455

© 2025 Le Cornet C et al. *JAMA Network Open*.

eTable 20. Number of Women Included in the Associations Between Estrogen-Only Therapy (ET) Use and Breast Cancer Intrinsic-Like Subtypes, in Postmenopausal Women, Stratified by ET (Corresponds to Results in eTables 5 and 8)

	Luminal A-like	Luminal B-like	Luminal B-ERBB2-like	ERBB2-enriched-like	Triple-negative	ER+	ER-	Invasive breast cancer	Controls
Lean/normal	4396	838	979	485	1003	10698	2465	13163	23345
Never	2690	526	674	354	654	5990	1597	7587	14303
Current use	355	61	81	25	59	923	153	1076	1817
Current, duration of use									
<5 years	50	10	20	4	17	165	44	209	392
≥5 years	208	33	44	18	37	459	91	550	861
Past use	359	60	82	34	84	759	165	924	1462
Past, duration of use									
<5 years	156	24	31	16	51	290	89	379	603
≥5 years	154	25	38	18	31	278	68	346	521
Past, time since last use									
<5 years	127	27	43	17	31	230	63	293	428
5-10 years	83	9	13	12	23	124	38	162	290
≥10 years	83	11	12	5	26	124	41	165	257
Overweight	3018	673	611	328	662	7514	1629	9143	15046
Never	1908	428	397	206	433	4341	1020	5361	8942
Current use	165	31	42	18	36	460	91	551	1008
Current, duration of use									
<5 years	15	8	6	8	7	64	26	90	233
≥5 years	91	11	26	9	25	217	56	273	428
Past use	182	45	39	17	34	470	70	540	807
Past, duration of use									
<5 years	63	22	21	8	23	174	39	213	276
≥5 years	74	15	13	7	8	154	25	179	273
Past, time since last use									
<5 years	41	13	8	5	8	92	20	112	178
5-10 years	41	9	10	5	10	76	18	94	114
≥10 years	43	12	13	5	12	84	21	105	115
Obese	1939	469	418	202	549	4625	1077	5702	7446
Never	1269	337	282	139	387	2873	734	3607	4443
Current use	83	15	13	7	30	214	49	263	469
Current, duration of use									
<5 years	15	2	0	1	9	37	17	54	124
≥5 years	41	6	8	6	19	98	27	125	211
Past use	145	31	22	15	34	283	55	338	429
Past, duration of use									
<5 years	54	15	9	6	16	112	25	137	175
≥5 years	63	12	10	8	17	106	27	133	122

© 2025 Le Cornet C et al. *JAMA Network Open*.

Past, time since last use									
<5 years	32	5	4	5	12	50	19	69	54
5-10 years	26	7	5	4	6	50	10	60	65
≥10 years	54	14	10	4	13	95	17	112	69

© 2025 Le Cornet C et al. *JAMA Network Open*.

eTable 21. Number of Women Included in the Associations Between Oral Contraceptive Use and Breast Cancer Subtypes, in Premenopausal Women Overall (Corresponds to Results in eTables 6 and 9)

	Luminal A-like	Luminal B-like	Luminal B-ERBB2-like	ERBB2-enriched-like	Triple-negative	ER+	ER-	Invasive breast cancer	Controls
Total	2590	753	896	438	1193	8624	3277	11901	21667
Never	755	178	203	98	260	1935	719	2654	4312
Current use	211	74	80	33	111	648	263	911	1568
Current, duration of use									
<5 years	20	10	7	2	11	65	29	94	147
≥5 years	163	58	69	29	95	537	221	758	1041
Past use	1160	355	440	208	656	3840	1653	5493	7437
Past, duration of use									
<5 years	456	151	174	64	238	1471	565	2036	2587
≥5 years	601	182	249	138	401	2162	1032	3194	3751
Past, time since last use									
<5 years	111	42	61	46	114	587	378	965	932
5-10 years	134	43	67	28	120	514	289	803	880
≥10 years	813	244	292	122	395	2500	921	3421	4375

© 2025 Le Cornet C et al. *JAMA Network Open*.

eTable 22. Number of Women Included in the Associations Between Estrogen-Progestin Therapy (EPT) Use and Breast Cancer Intrinsic-Like Subtypes, in Postmenopausal Women, Stratified by Body Mass Index (BMI) in a Restricted Set of Studies With at Least 10 Cases Reporting EPT Use (Corresponds to Results in eTable 10)

	Luminal A-like	Luminal B-like	Luminal B-ERBB2-like	ERBB2-enriched-like	Triple-negative	ER+	ER-	Invasive breast cancer	Controls
Lean/normal	2883	543	712	329	640	6630	1466	8096	13032
Never	1603	324	437	235	432	3740	957	4697	8438
Current use	802	112	178	41	93	1666	225	1891	2049
Current, duration of use									
<5 years	155	19	44	11	21	339	64	403	520
≥5 years	517	68	101	24	64	888	137	1025	1050
Past use	334	68	78	43	90	651	183	834	1551
Past, duration of use									
<5 years	119	26	34	22	42	231	92	323	630
≥5 years	188	39	37	20	46	332	81	413	763
Past, time since last use									
<5 years	163	37	40	26	60	287	109	396	752
5-10 years	75	16	20	11	19	130	34	164	300
≥10 years	35	4	8	4	5	54	13	67	151
Overweight	1513	367	352	153	345	3981	821	4802	7120
Never	1024	272	247	117	272	2644	610	3254	5096
Current use	231	34	52	18	30	562	76	638	670
Current, duration of use									
<5 years	59	5	14	6	5	131	21	152	199
≥5 years	111	22	27	8	19	248	42	290	250
Past use	159	32	23	12	31	304	55	359	613
Past, duration of use									
<5 years	57	13	9	6	9	114	22	136	251
≥5 years	81	13	11	6	18	141	28	169	271
Past, time since last use									
<5 years	71	13	11	8	15	125	27	152	229
5-10 years	33	6	6	2	5	54	9	63	112
≥10 years	17	5	3	2	4	32	7	39	67
Obese	991	240	236	98	318	2505	587	3092	3506
Never	748	200	177	86	278	1787	480	2267	2655
Current use	61	9	20	2	16	174	25	199	220
Current, duration of use									
<5 years	16	2	5	0	8	48	13	61	70
≥5 years	27	5	10	2	5	66	9	75	77
Past use	99	16	15	6	17	189	26	215	236
Past, duration of use									
<5 years	25	7	6	2	5	69	10	79	101

© 2025 Le Cornet C et al. *JAMA Network Open*.

≥5 years	51	7	6	4	10	78	14	92	88
Past, time since last use									
<5 years	25	2	3	4	7	39	13	52	65
5-10 years	29	4	4	1	4	46	5	51	47
≥10 years	15	4	5	1	2	36	3	39	18

© 2025 Le Cornet C et al. *JAMA Network Open*.

eTable 23. Number of Women Included in the Associations Between Estrogen-Only Therapy (ET) and Breast Cancer Intrinsic-Like Subtypes, in Postmenopausal Women, Stratified by Body Mass Index (BMI) in a Restricted Set of Studies With at Least 10 Cases Reporting ET Use (Corresponds to Results in eTable 11)

	Luminal A-like	Luminal B-like	Luminal B-ERBB2-like	ERBB2-enriched-like	Triple-negative	ER+	ER-	Invasive breast cancer	Controls
Lean/normal	2883	543	712	329	640	6630	1466	8096	13032
Never	2014	373	522	260	475	4267	1053	5320	8734
Current use	354	61	81	25	58	922	152	1074	1809
Current, duration of use									
<5 years	49	10	20	4	17	164	44	208	389
≥5 years	208	33	44	18	36	459	90	549	856
Past use	353	60	81	33	78	751	157	908	1438
Past, duration of use									
<5 years	152	24	30	16	47	285	84	369	590
≥5 years	152	25	38	17	30	276	66	342	516
Past, time since last use									
<5 years	127	27	42	16	27	229	57	286	415
5-10 years	81	9	13	12	22	122	37	159	287
≥10 years	79	11	12	5	25	119	40	159	249
Overweight	1513	367	352	153	345	3981	821	4802	7120
Never	1073	263	243	109	263	2579	575	3154	4580
Current use	165	30	42	18	36	459	91	550	1004
Current, duration of use									
<5 years	15	7	6	8	7	63	26	89	231
≥5 years	91	11	26	9	25	217	56	273	426
Past use	170	43	38	15	33	455	67	522	784
Past, duration of use									
<5 years	60	21	20	7	22	169	37	206	264
≥5 years	68	14	13	7	8	147	25	172	269
Past, time since last use									
<5 years	34	13	8	4	7	85	18	103	169
5-10 years	37	9	10	5	10	72	18	90	110
≥10 years	42	10	12	4	12	80	20	100	106
Obese	991	240	236	98	318	2505	587	3092	3506
Never	687	177	176	72	247	1654	427	2081	2217
Current use	83	15	13	7	30	214	49	263	466
Current, duration of use									
<5 years	15	2	0	1	9	37	17	54	122
≥5 years	41	6	8	6	19	98	27	125	210
Past use	139	29	22	15	32	275	53	328	415
Past, duration of use									
<5 years	53	14	9	6	14	110	23	133	168

© 2025 Le Cornet C et al. *JAMA Network Open*.

≥5 years	61	11	10	8	17	103	27	130	119
Past, time since last use									
<5 years	30	5	4	5	11	48	18	66	52
5-10 years	25	7	5	4	5	49	9	58	60
≥10 years	51	12	10	4	13	90	17	107	63

© 2025 Le Cornet C et al. *JAMA Network Open*.

eTable 24. Number of Women Included in the Associations Between Oral Contraceptive (OC) Use and Breast Cancer Subtypes, in Premenopausal Women Overall in a Restricted Set of Studies With At Least 10 Cases Reporting OC Use (Corresponds to Results in eTable 12)

	Luminal A-like	Luminal B-like	Luminal B-ERBB2-like	ERBB2-enriched-like	Triple-negative	ER+	ER-	Invasive breast cancer	Controls
All	2009	572	700	330	1004	5931	2475	8406	11662
Never	622	142	180	86	233	1477	559	2036	2531
Current use	211	73	80	33	111	641	260	901	1564
Current, duration									
<5 years	20	10	7	2	11	65	29	94	145
≥5 years	163	57	69	29	95	530	218	748	1039
Past use	1157	351	434	206	654	3634	1592	5226	6983
Past, duration of									
<5 years	455	148	170	63	237	1361	534	1895	2326
≥5 years	600	181	247	137	400	2071	1003	3074	3567
Past, time since									
<5 years	111	42	61	46	113	567	367	934	917
>5-10 years	134	43	67	28	120	503	285	788	859
≥10 years	813	243	288	122	395	2394	887	3281	4195

© 2025 Le Cornet C et al. *JAMA Network Open*.

eTable 25. Number of Women Included in the Associations Between Estrogen-Progestin Therapy (EPT) Use and Breast Cancer Intrinsic-Like Subtypes, in Postmenopausal Women, Stratified by Body Mass Index (BMI) and Study Design (Corresponds to Results in eTable 13)

	Luminal A-like	Luminal B-like	Luminal B-ERBB2-like	ERBB2-enriched-like	Triple-negative	ER+	ER-	Invasive breast cancer	Controls
Lean/normal									
Never									
Cohort	738	165	145	43	111	2061	261	2322	6636
Case-control	1538	308	440	286	499	3425	1241	4666	7483
Current use									
Cohort	178	38	32	9	8	617	34	651	658
Case-control	624	74	146	32	85	1050	193	1243	1397
Past use									
Cohort	56	15	13	5	8	160	25	185	317
Case-control	278	53	65	38	82	492	158	650	1237
Overweight									
Never									
Cohort	760	167	112	48	106	1927	253	2180	5271
Case-control	1089	267	293	166	334	2481	801	3282	4241
Current use									
Cohort	116	12	19	9	9	314	25	339	322
Case-control	115	22	33	9	21	249	51	300	350
Past use									
Cohort	47	11	5	2	10	103	16	119	217
Case-control	112	21	18	10	21	201	39	240	398
Obese									
Never									
Cohort	482	122	86	27	64	1169	167	1336	2924
Case-control	840	236	198	125	352	1834	618	2452	1957
Current use									
Cohort	41	7	12	0	5	120	7	127	135
Case-control	20	2	8	2	11	54	18	72	90
Past use									
Cohort	52	6	5	1	10	92	11	103	131
Case-control	47	10	10	5	7	97	15	112	105

© 2025 Le Cornet C et al. *JAMA Network Open*.

eTable 26. Number of Women Included in the Associations Between Estrogen-Only Therapy (ET) Use and Breast Cancer Intrinsic-Like Subtypes, in Postmenopausal Women, Stratified by Body Mass Index (BMI) and Study Design (Corresponds to Results in eTable 14)

	Luminal A-like	Luminal B-like	Luminal B-ERBB2-like	ERBB2-enriched-like	Triple-negative	ER+	ER-	Invasive breast cancer	Controls
Lean/normal									
Never									
Cohort	748	167	131	49	107	1961	257	2218	6153
Case-control	1942	359	543	305	547	4029	1340	5369	8150
Current use									
Cohort	128	27	33	3	9	443	37	480	835
Case-control	227	34	48	22	50	480	116	596	982
Past use									
Cohort	73	15	17	3	6	274	18	292	490
Case-control	286	45	65	31	78	485	147	632	972
Overweight									
Never									
Cohort	762	157	106	48	104	1810	243	2053	4753
Case-control	1146	271	291	158	329	2531	777	3308	4189
Current use									
Cohort	84	17	16	5	15	272	32	304	579
Case-control	81	14	26	13	21	188	59	247	429
Past use									
Cohort	69	13	10	1	5	224	10	234	404
Case-control	113	32	29	16	29	246	60	306	403
Obese									
Never									
Cohort	483	116	91	22	69	1131	160	1291	2587
Case-control	786	221	191	117	318	1742	574	2316	1856
Current use									
Cohort	49	9	9	1	5	138	12	150	316
Case-control	34	6	4	6	25	76	37	113	153
Past use									
Cohort	41	10	3	4	5	100	12	112	263
Case-control	104	21	19	11	29	183	43	226	166

© 2025 Le Cornet C et al. *JAMA Network Open*.

eTable 27. Number of Women Included in the Associations Between Oral Contraceptive Use and Breast Cancer Intrinsic-Like Subtypes, In Premenopausal Women Overall Stratified by Study Design (Corresponds to Results in eTable 15)

	Luminal A-like	Luminal B-like	Luminal B-ERBB2-like	ERBB2-enriched-like	Triple-negative	ER+	ER-	Invasive breast cancer	Controls
Lean/normal									
Never									
Cohort	174	55	49	10	25	589	96	685	1763
Case-control	581	123	154	88	235	1346	623	1969	2549
Current use									
Cohort	68	16	14	3	21	169	33	202	752
Case-control	143	58	66	30	90	479	230	709	816
Past use									
Cohort	396	108	126	39	90	1258	265	1523	3688
Case-control	764	247	314	169	566	2582	1388	3970	3749

© 2025 Le Cornet C et al. *JAMA Network Open*.

eTable 28. Number of Women Included in the Analysis on the Heterogeneity Between Studies Set Up Before and After 2000 for the Associations Between Estrogen-Progestin Therapy (EPT) Use and Breast Cancer Intrinsic-Like Subtypes, in Postmenopausal Women, Stratified by Body Mass Index (BMI) (Corresponds to Results in eTable 16)

	Luminal A-like	Luminal B-like	Luminal B-ERBB2-like	ERBB2-enriched-like	Triple-negative	ER+	ER-	Invasive breast cancer	Controls
Lean/normal									
Before	1481	294	254	96	246	4127	642	4769	7100
After	2566	483	607	304	586	4916	1277	6193	14188
Never									
Before	695	149	131	48	134	2066	346	2412	4129
After	1378	289	368	209	327	2543	764	3307	8579
Current use									
Before	42	10	11	4	7	135	25	160	265
After	269	53	61	38	76	454	140	594	1207
Past use									
Before	167	35	31	7	8	582	47	629	680
After	601	75	130	30	82	898	164	1062	1234
Overweight									
Before	1511	291	227	119	236	3623	595	4218	5855
After	1173	292	300	131	279	2527	611	3138	7660
Never									
Before	844	167	125	62	141	2086	356	2442	3595
After	763	200	209	81	169	1492	373	1865	4764
Current use									
Before	35	9	5	2	8	80	14	94	184
After	96	17	15	9	19	166	34	200	374
Past use									
Before	104	12	19	8	6	284	28	312	324
After	106	19	30	9	17	188	36	224	257
Obese									
Before	995	231	178	55	173	2259	358	2617	3181
After	513	126	142	87	135	1187	296	1483	3313
Never									
Before	597	151	105	36	101	1391	234	1625	1944

© 2025 Le Cornet C et al. *JAMA Network Open*.

After	351	101	96	60	90	751	198	949	2140
Current use									
Before	38	5	3	1	8	68	9	77	80
After	34	7	4	4	4	59	9	68	108
Past use									
Before	37	7	11	0	4	105	7	112	122
After	15	2	7	1	6	33	8	41	56

© 2025 Le Cornet C et al. *JAMA Network Open*.

eTable 29. Number of Women Included in the Analysis on the Heterogeneity Between Studies Set Up Before and After 2000 for the Associations Between Estrogen-Only Therapy (ET) Use and Breast Cancer Intrinsic-Like Subtypes, In Postmenopausal Women, Stratified by Body Mass Index (BMI) (Corresponds to Results in eTable 17)

	Luminal A-like	Luminal B-like	Luminal B-ERBB2-like	ERBB2-enriched-like	Triple-negative	ER+	ER-	Invasive breast cancer	Controls
Lean/normal									
Before	1481	294	254	96	246	4127	642	4769	7100
After	2566	483	607	304	586	4916	1277	6193	14188
Never									
Before	717	151	119	51	131	1932	335	2267	3693
After	1779	341	468	231	410	3160	900	4060	9314
Current use									
Before	113	26	31	3	10	472	56	528	856
After	208	31	37	20	28	321	68	389	749
Past use									
Before	67	13	18	4	12	249	30	279	445
After	258	42	55	27	48	408	100	508	892
Overweight									
Before	1511	291	227	119	236	3623	595	4218	5855
After	1173	292	300	131	279	2527	611	3138	7660
Never									
Before	856	161	122	62	139	1990	347	2337	3182
After	834	212	214	88	182	1597	395	1992	4795
Current use									
Before	79	16	15	5	14	267	37	304	538
After	57	11	18	4	9	95	19	114	257
Past use									
Before	68	14	9	3	5	195	13	208	358
After	67	13	22	7	14	145	28	173	328
Obese									
Before	995	231	178	55	173	2259	358	2617	3181
After	513	126	142	87	135	1187	296	1483	3313
Never									
Before	613	147	109	33	107	1372	232	1604	1716

© 2025 Le Cornet C et al. *JAMA Network Open*.

After	350	102	101	62	90	763	199	962	2102
Current use									
Before	34	11	3	4	7	88	12	100	189
After	32	7	4	2	4	52	7	59	119
Past use									
Before	38	9	8	1	3	113	11	124	250
After	18	1	1	1	6	26	8	34	78

© 2025 Le Cornet C et al. *JAMA Network Open*.

eFigure 1. Odds Ratios and 95% CIs for Case-Control Analyses of Associations Between Estrogen-Progestin Therapy (EPT) Use and Breast Cancer Overall as well as Estrogen Receptor (ER)-Positive and ER-Negative Breast Cancer According to Body Mass Index (BMI)

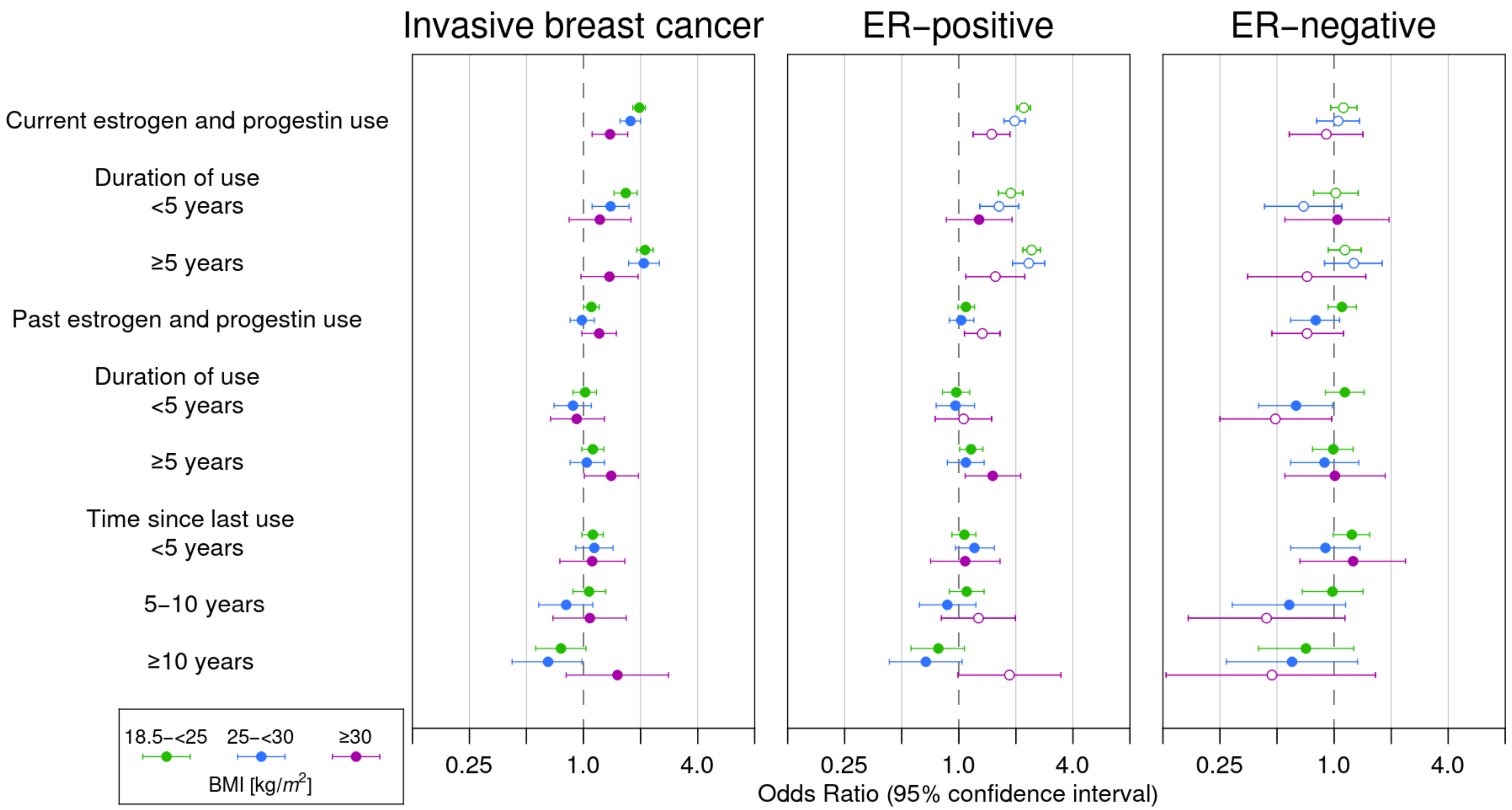

Three polytomous logistic regression models were fit: EPT use, duration of EPT use, and time since last EPT use in association with subtypes. The multivariable model was adjusted for reference age (age at diagnosis for cases, age at interview for controls), study, estrogen therapy use, and oral contraceptive use. Heterogeneity in EPT use associations between ER+ and ER- subtypes was evaluated by comparing models that assume common associations across subtypes to those allowing different associations by subtype. Statistical significance was considered with P-values less than 0.05. Subtype heterogeneity is denoted with open circles. The error bars represent 95% confidence intervals.

© 2025 Le Cornet C et al. *JAMA Network Open*.

eFigure 2. Odds Ratios and 95% CIs for Case-Control Analyses of Associations Between Estrogen Therapy (ET) Use and Breast Cancer Overall as well as Estrogen Receptor (ER)-Positive and ER-Negative Breast Cancer According to Body Mass Index (BMI)

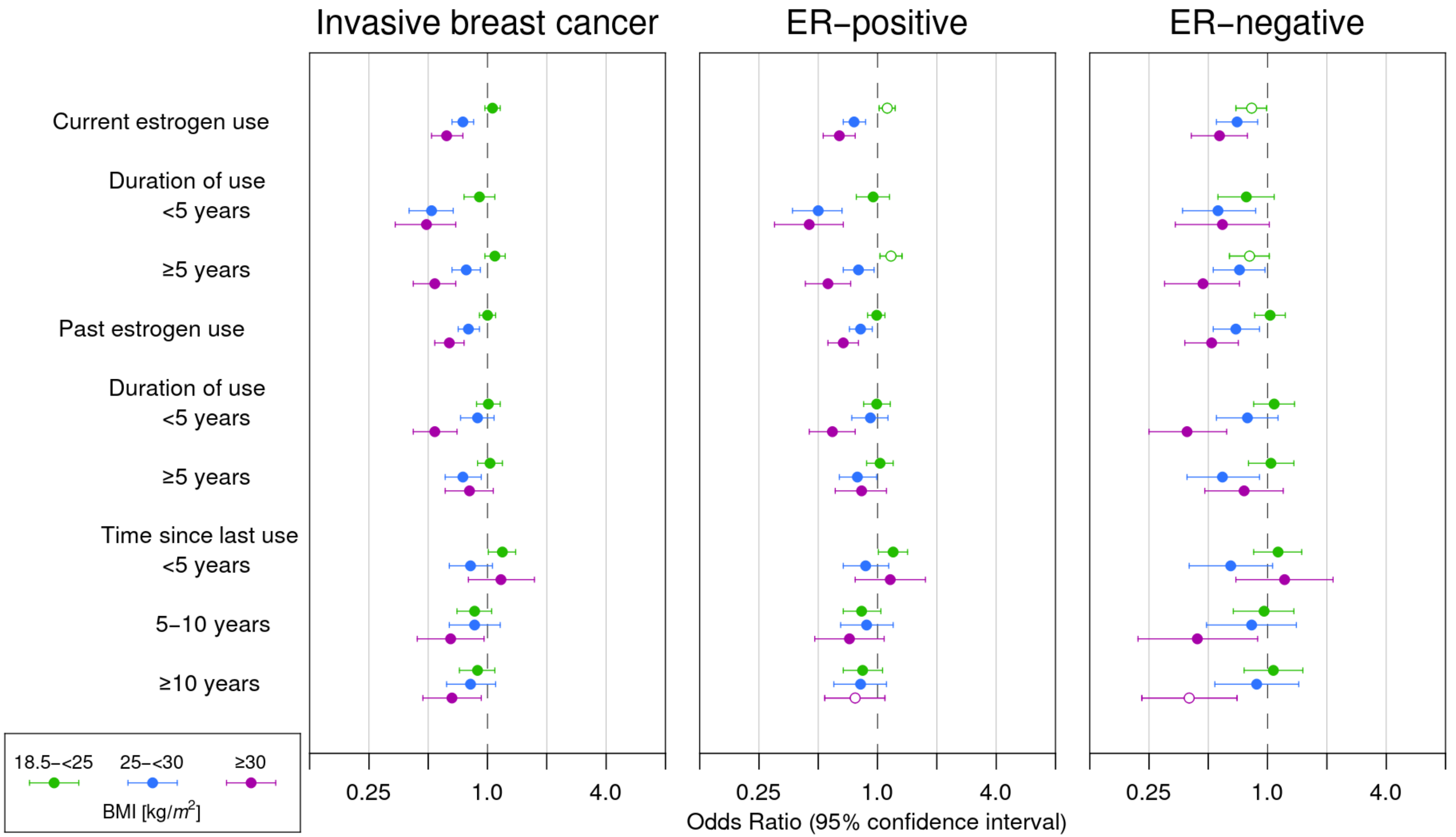

Three polytomous logistic regression models were fit: ET use, duration of ET use, and time since last ET use in association with subtypes. The multivariable model was adjusted for reference age (age at diagnosis for cases, age at interview for controls), study, estrogen-progestin therapy use, and oral contraceptive use. Heterogeneity in ET use associations between ER+ and ER- subtypes was evaluated by comparing models that assume common associations across subtypes to those allowing different associations by subtype. Statistical significance was considered with P-values less than 0.05. Subtype heterogeneity is denoted with open circles. The error bars represent 95% confidence intervals.

© 2025 Le Cornet C et al. *JAMA Network Open*.

eFigure 3. Odds Ratios and 95% CIs for Case-Control Analyses of Associations Between Oral Contraceptive (OC) Use and Breast Cancer Overall as well as Estrogen Receptor (ER)-Positive and ER-Negative Breast Cancer

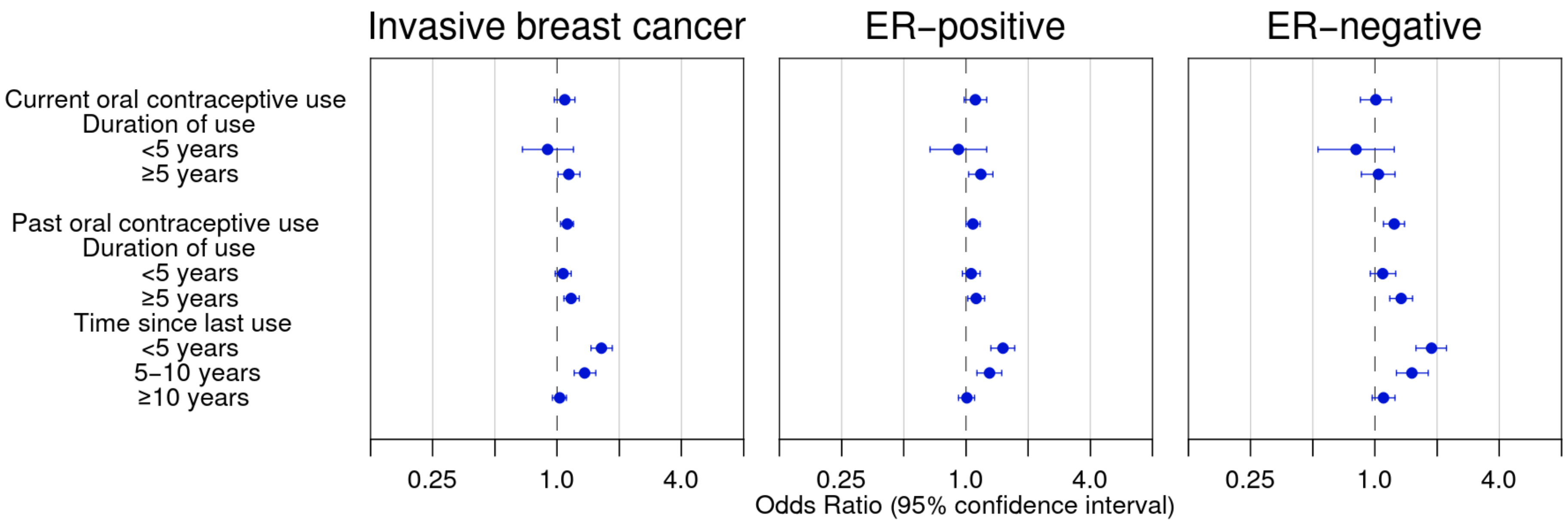

Three polytomous logistic regression models were fit: OC use, duration of OC use, and time since last OC use in association with subtypes. The multivariable model was adjusted for reference age (age at diagnosis for cases, age at interview for controls), study, and body mass index. Heterogeneity in ET use associations between ER+ and ER- subtypes was evaluated by comparing models that assume common associations across subtypes to those allowing different associations by subtype. Statistical significance was considered with P-values less than 0.05. Subtype heterogeneity is denoted with open circles. The error bars represent 95% confidence intervals of associations between oral contraceptive use and breast cancer overall and by ER status, in premenopausal women overall.

© 2025 Le Cornet C et al. *JAMA Network Open*.

eReferences

1. Jung AY, Ahearn TU, Behrens S, Middha P, Bolla MK, Wang Q*, et al.* Distinct Reproductive Risk Profiles for Intrinsic-Like Breast Cancer Subtypes: Pooled Analysis of Population-Based Studies. J Natl Cancer Inst **2022**;114:1706-19
2. Koutros S, Alavanja MC, Lubin JH, Sandler DP, Hoppin JA, Lynch CF*, et al.* An update of cancer incidence in the Agricultural Health Study. J Occup Environ Med **2010**;52:1098-105
3. Calle EE, Rodriguez C, Jacobs EJ, Almon ML, Chao A, McCullough ML*, et al.* The American Cancer Society Cancer Prevention Study II Nutrition Cohort: rationale, study design, and baseline characteristics. Cancer **2002**;94:2490-501
4. Bernstein L, Allen M, Anton-Culver H, Deapen D, Horn-Ross PL, Peel D*, et al.* High breast cancer incidence rates among California teachers: results from the California Teachers Study (United States). Cancer Causes Control **2002**;13:625-35
5. Riboli E, Hunt KJ, Slimani N, Ferrari P, Norat T, Fahey M*, et al.* European Prospective Investigation into Cancer and Nutrition (EPIC): study populations and data collection. Public Health Nutr **2002**;5:1113-24
6. Li J, Humphreys K, Eriksson M, Dar H, Brandberg Y, Hall P*, et al.* Worse quality of life in young and recently diagnosed breast cancer survivors compared with female survivors of other cancers: A cross-sectional study. Int J Cancer **2016**;139:2415-25
7. Milne RL, Fletcher AS, MacInnis RJ, Hodge AM, Hopkins AH, Bassett JK*, et al.* Cohort Profile: The Melbourne Collaborative Cohort Study (Health 2020). Int J Epidemiol **2017**;46:1757-i
8. Kolonel LN, Henderson BE, Hankin JH, Nomura AM, Wilkens LR, Pike MC*, et al.* A multiethnic cohort in Hawaii and Los Angeles: baseline characteristics. Am J Epidemiol **2000**;151:346-57
9. Olsson HL, Ingvar C, Bladstrom A. Hormone replacement therapy containing progestins and given continuously increases breast carcinoma risk in Sweden. Cancer **2003**;97:1387-92
10. Olson JE, Sellers TA, Scott CG, Schueler BA, Brandt KR, Serie DJ*, et al.* The influence of mammogram acquisition on the mammographic density and breast cancer association in the Mayo Mammography Health Study cohort. Breast Cancer Res **2012**;14:R147
11. Hankinson SE, Willett WC, Manson JE, Colditz GA, Hunter DJ, Spiegelman D*, et al.* Plasma sex steroid hormone levels and risk of breast cancer in postmenopausal women. J Natl Cancer Inst **1998**;90:1292-9
12. Tworoger SS, Missmer SA, Eliassen AH, Spiegelman D, Folkerd E, Dowsett M*, et al.* The association of plasma DHEA and DHEA sulfate with breast cancer risk in predominantly premenopausal women. Cancer Epidemiol Biomarkers Prev **2006**;15:967-71
13. Pfeiffer RM, Park Y, Kreimer AR, Lacey JV, Jr., Pee D, Greenlee RT*, et al.* Risk prediction for breast, endometrial, and ovarian cancer in white women aged 50 y or older: derivation and validation from population-based cohort studies. PLoS Med **2013**;10:e1001492
14. Suzuki R, Ye W, Rylander-Rudqvist T, Saji S, Colditz GA, Wolk A. Alcohol and postmenopausal breast cancer risk defined by estrogen and progesterone receptor status: a prospective cohort study. J Natl Cancer Inst **2005**;97:1601-8
15. Dite GS, Jenkins MA, Southey MC, Hocking JS, Giles GG, McCredie MR*, et al.* Familial risks, early-onset breast cancer, and BRCA1 and BRCA2 germline mutations. J Natl Cancer Inst **2003**;95:448-57
16. Fritschi L, Erren TC, Glass DC, Girschik J, Thomson AK, Saunders C*, et al.* The association between different night shiftwork factors and breast cancer: a case-control study. Br J Cancer **2013**;109:2472-80
17. Rennert G, Pinchev M, Rennert HS. Use of bisphosphonates and risk of postmenopausal breast cancer. J Clin Oncol **2010**;28:3577-81
18. Grundy A, Schuetz JM, Lai AS, Janoo-Gilani R, Leach S, Burstyn I*, et al.* Shift work, circadian gene variants and risk of breast cancer. Cancer Epidemiol **2013**;37:606-12
19. Menegaux F, Truong T, Anger A, Cordina-Duverger E, Lamkarkach F, Arveux P*, et al.* Night work and breast cancer: a population-based case-control study in France (the CECILE study). Int J Cancer **2013**;132:924-31
20. Widschwendter M, Apostolidou S, Raum E, Rothenbacher D, Fiegl H, Menon U*, et al.* Epigenotyping in peripheral blood cell DNA and breast cancer risk: a proof of principle study. PLoS One **2008**;3:e2656
21. Pesch B, Ko Y, Brauch H, Hamann U, Harth V, Rabstein S*, et al.* Factors modifying the association between hormone-replacement therapy and breast cancer risk. Eur J Epidemiol **2005**;20:699-711
22. Chang-Claude J, Eby N, Kiechle M, Bastert G, Becher H. Breastfeeding and breast cancer risk by age 50 among women in Germany. Cancer Causes Control **2000**;11:687-95
23. Hartikainen JM, Tuhkanen H, Kataja V, Dunning AM, Antoniou A, Smith P*, et al.* An autosome-wide scan for linkage disequilibrium-based association in sporadic breast cancer cases in eastern Finland: three candidate regions found. Cancer Epidemiol Biomarkers Prev **2005**;14:75-80

© 2025 Le Cornet C et al. *JAMA Network Open*.

24. Wu AH, Yu MC, Tseng CC, Stanczyk FZ, Pike MC. Dietary patterns and breast cancer risk in Asian American women. Am J Clin Nutr **2009**;89:1145-54
25. Flesch-Janys D, Slanger T, Mutschelknauss E, Kropp S, Obi N, Vettorazzi E*, et al.* Risk of different histological types of postmenopausal breast cancer by type and regimen of menopausal hormone therapy. Int J Cancer **2008**;123:933-41
26. Hadjisavvas A, Loizidou MA, Middleton N, Michael T, Papachristoforou R, Kakouri E*, et al.* An investigation of breast cancer risk factors in Cyprus: a case control study. BMC Cancer **2010**;10:447
27. Zheng W, Long J, Gao YT, Li C, Zheng Y, Xiang YB*, et al.* Genome-wide association study identifies a new breast cancer susceptibility locus at 6q25.1. Nat Genet **2009**;41:324-8
28. Newman B, Moorman PG, Millikan R, Qaqish BF, Geradts J, Aldrich TE*, et al.* The Carolina Breast Cancer Study: integrating population-based epidemiology and molecular biology. Breast Cancer Res Treat **1995**;35:51-60
29. Garcia-Closas M, Egan KM, Newcomb PA, Brinton LA, Titus-Ernstoff L, Chanock S*, et al.* Polymorphisms in DNA double-strand break repair genes and risk of breast cancer: two population-based studies in USA and Poland, and meta-analyses. Hum Genet **2006**;119:376-88
30. Evans DG, Astley S, Stavrinos P, Harkness E, Donnelly LS, Dawe S*, et al.* Improvement in risk prediction, early detection and prevention of breast cancer in the NHS Breast Screening Programme and family history clinics: a dual cohort study. Southampton (UK)2016.
31. Wedren S, Lovmar L, Humphreys K, Magnusson C, Melhus H, Syvanen AC*, et al.* Oestrogen receptor alpha gene haplotype and postmenopausal breast cancer risk: a case control study. Breast Cancer Res **2004**;6:R437-49

© 2025 Le Cornet C et al. *JAMA Network Open*.

eAppendix 1. BCAC Funders

The Australian Breast Cancer Family Study (ABCFS) was supported by grant UM1 CA164920 from the National Cancer Institute (USA). The content of this manuscript does not necessarily reflect the views or policies of the National Cancer Institute or any of the collaborating centers in the Breast Cancer Family Registry (BCFR), nor does mention of trade names, commercial products, or organizations imply endorsement by the USA Government or the BCFR. The ABCFS was also supported by the National Health and Medical Research Council of Australia, the New South Wales Cancer Council, the Victorian Health Promotion Foundation (Australia) and the Victorian Breast Cancer Research Consortium. J.L.H. is a National Health and Medical Research Council (NHMRC) Senior Principal Research Fellow. M.C.S. is a NHMRC Senior Research Fellow.

The AHS study is supported by the intramural research program of the National Institutes of Health, the National Cancer Institute (grant number Z01-CP010119), and the National Institute of Environmental Health Sciences (grant number Z01-ES049030).

The BCEES was funded by the National Health and Medical Research Council, Australia and the Cancer Council Western Australia and acknowledges funding from the National Breast Cancer Foundation (JS).

The BCINIS study is supported in part by the Breast Cancer Research Foundation (BCRF).

CBCS is funded by the Canadian Cancer Society (grant # 313404) and the Canadian Institutes of Health Research.

The CECILE study was supported by Fondation de France, Institut National du Cancer (INCa), Ligue Nationale contre le Cancer, Agence Nationale de Sécurité Sanitaire, de l'Alimentation, de l'Environnement et du Travail (ANSES), Agence Nationale de la Recherche (ANR).

The American Cancer Society funds the creation, maintenance, and updating of the CPS-II cohort.

The California Teachers Study (CTS) and the research reported in this publication were supported by the National Cancer Institute of the National Institutes of Health under award number U01-CA199277; P30-CA033572; P30-CA023100; UM1-CA164917; and R01-CA077398. The content is solely the responsibility of the authors and does not necessarily represent the official views of the National Cancer Institute or the National Institutes of Health. The collection of cancer incidence data used in the California Teachers Study was supported by the California Department of Public Health pursuant to California Health and Safety Code Section 103885; Centers for Disease Control and Prevention's National Program of Cancer Registries, under cooperative agreement 5NU58DP006344; the National Cancer Institute's Surveillance, Epidemiology and End Results Program under contract HHSN261201800032I awarded to the University of California, San Francisco, contract HHSN261201800015I awarded to the University of Southern California, and contract HHSN261201800009I awarded to the Public Health Institute.

The coordination of EPIC is financially supported by the European Commission (DG-SANCO) and the International Agency for Research on Cancer. The national cohorts are supported by: Ligue Contre le Cancer, Institut Gustave Roussy, Mutuelle Générale de l'Education Nationale, Institut National de la Santé et de la Recherche Médicale (INSERM) (France); German Cancer Aid, German Cancer Research Center (DKFZ), Federal Ministry of Education and Research (BMBF) (Germany); the Hellenic Health Foundation, the Stavros Niarchos Foundation (Greece); Associazione Italiana per la Ricerca sul Cancro-AIRC-Italy and National Research Council (Italy); Dutch Ministry of Public Health, Welfare and Sports (VWS), Netherlands Cancer Registry (NKR), LK Research Funds, Dutch Prevention Funds, Dutch ZON (Zorg Onderzoek Nederland), World Cancer Research Fund (WCRF), Statistics Netherlands (The Netherlands); Health Research Fund (FIS), PI13/00061 to Granada, PI13/01162 to EPIC-Murcia, Regional Governments of Andalucía, Asturias, Basque Country, Murcia and Navarra, ISCIII RETIC (RD06/0020) (Spain); Cancer Research UK (14136 to EPIC-Norfolk; C570/A16491 and C8221/A19170 to EPIC-Oxford), Medical Research Council (1000143 to EPIC-Norfolk, MR/M012190/1 to EPIC-Oxford) (United Kingdom).

The ESTHER study was supported by a grant from the Baden Württemberg Ministry of Science, Research and Arts. Additional cases were recruited in the context of the VERDI study, which was supported by a grant from the German Cancer Aid (Deutsche Krebshilfe).

PROCAS are funded from NIHR grant PGfAR 0707-10031. DGE, AH and WGN are supported by the NIHR Manchester Biomedical Research Centre (IS-BRC-1215-20007). The GC-HBOC (German Consortium of Hereditary Breast and Ovarian Cancer) is supported by the German Cancer Aid (grant no 110837 and 70114178, coordinator: Rita K. Schmutzler, Cologne) and the Federal Ministry of Education and Research, Germany (grant no 01GY1901). This work was also funded by the European Regional Development Fund and Free State of Saxony, Germany (LIFE - Leipzig Research Centre for Civilization Diseases, project numbers 713-241202, 713-241202, 14505/2470, 14575/2470).
© 2025 Le Cornet C et al. *JAMA Network Open*.

The GENICA was funded by the Federal Ministry of Education and Research (BMBF) Germany grants 01KW9975/5, 01KW9976/8, 01KW9977/0 and 01KW0114, the Robert Bosch Foundation, Stuttgart, Deutsches Krebsforschungszentrum (DKFZ), Heidelberg, the Institute for Prevention and Occupational Medicine of the German Social Accident Insurance, Institute of the Ruhr University Bochum (IPA), Bochum, as well as the Department of Internal Medicine, Johanniter GmbH Bonn, Johanniter Krankenhaus, Bonn, Germany.

The GESBC was supported by the Deutsche Krebshilfe e. V. [70492] and the German Cancer Research Center (DKFZ).

The KARMA study was supported by Märit and Hans Rausings Initiative Against Breast Cancer.

The KBCP was financially supported by the special Government Funding (VTR) of Kuopio University Hospital grants, Cancer Fund of North Savo, the Finnish Cancer Organizations, and by the strategic funding of the University of Eastern Finland.

LAABC is supported by grants (1RB-0287, 3PB-0102, 5PB-0018, 10PB-0098) from the California Breast Cancer Research Program. Incident breast cancer cases were collected by the USC Cancer Surveillance Program (CSP) which is supported under subcontract by the California Department of Health. The CSP is also part of the National Cancer Institute's Division of Cancer Prevention and Control Surveillance, Epidemiology, and End Results Program, under contract number N01CN25403.

The MARIE study was supported by the Deutsche Krebshilfe e.V. [70-2892-BR I, 106332, 108253, 108419, 110826, 110828], the Hamburg Cancer Society, the German Cancer Research Center (DKFZ) and the Federal Ministry of Education and Research (BMBF) Germany [01KH0402].

The MASTOS study was supported by "Cyprus Research Promotion Foundation" grants 0104/13 and 0104/17, and the Cyprus Institute of Neurology and Genetics. MBCSG is supported by grants from the Italian Association for Cancer Research (AIRC).

The Melbourne Collaborative Cohort Study (MCCS) cohort recruitment was funded by VicHealth and Cancer Council Victoria. The MCCS was further augmented by Australian National Health and Medical Research Council grants 209057, 396414 and 1074383 and by infrastructure provided by Cancer Council Victoria. Cases and their vital status were ascertained through the Victorian Cancer Registry and the Australian Institute of Health and Welfare, including the Australian Cancer Database.

The MEC was supported by NIH grants CA63464, CA54281, CA098758, CA132839 and CA164973.

The MISS study was supported by funding from ERC-2011-294576 Advanced grant, Swedish Cancer Society CAN 2018/675, Swedish Research Council, Local hospital funds, Berta Kamprad Foundation FBKS 2021-19, Gunnar Nilsson.

The MMHS study was supported by NIH grants CA97396, CA128931, CA116201, CA140286 and CA177150.

The NBHS was supported by NIH grant R01CA100374. Biological sample preparation was conducted the Survey and Biospecimen Shared Resource, which is supported by P30 CA68485.

The Carolina Breast Cancer Study (NCBCS) was funded by Komen Foundation, the National Cancer Institute (P50 CA058223, R01 CA253450), and the North Carolina University Cancer Research Fund.

The NHS was supported by NIH grants P01 CA87969, UM1 CA186107, and U19 CA148065.

The NHS2 was supported by NIH grants UM1 CA176726 and U19 CA148065.

The PBCS was funded by Intramural Research Funds of the National Cancer Institute, Department of Health and Human Services, USA.

Genotyping for PLCO was supported by the Intramural Research Program of the National Institutes of Health, NCI, Division of Cancer Epidemiology and Genetics. The PLCO is supported by the Intramural Research Program of the Division of Cancer Epidemiology and Genetics and supported by contracts from the Division of Cancer Prevention, National Cancer Institute, National Institutes of Health.

The SASBAC study was supported by funding from the Agency for Science, Technology and Research of Singapore (A*STAR), the US National Institute of Health (NIH) and the Susan G. Komen Breast Cancer Foundation.

The SBCGS was supported primarily by NIH grants R01CA64277, R01CA148667, UMCA182910, and R37CA70867. Biological sample preparation was conducted the Survey and Biospecimen Shared Resource, which is supported by

© 2025 Le Cornet C et al. *JAMA Network Open*.

P30 CA68485. The scientific development and funding of this project were, in part, supported by the Genetic Associations and Mechanisms in Oncology (GAME-ON) Network U19 CA148065.

The SMC is funded by the Swedish Cancer Foundation and the Swedish Research Council (VR 2017-00644) grant for the Swedish Infrastructure for Medical Population-based Life-course Environmental Research (SIMPLER).

© 2025 Le Cornet C et al. *JAMA Network Open*.

eAppendix 2. BCAC Additional Contributions

ABCFS thank Maggie Angelakos, Judi Maskiell, Gillian Dite.

BCEES thanks Allyson Thomson, Christobel Saunders, Jennifer Girschik, Jane Heyworth and Terry Boyle.

The BCINIS study would not have been possible without the major contribution of Ms. H. Rennert, and the contributions of Dr M. Pinchev, Dr O. Barnet, Dr N. Gronich, Dr K. Landsman, Dr A. Flugelman, Dr W. Saliba, Dr E. Liani, Dr I. Cohen, Dr S. Kalet, Dr V. Friedman of the NICCC in Haifa, and all the contributing family medicine, surgery, pathology and oncology teams in all medical institutes in Northern Israel.

CBCS thanks study participants, co-investigators, collaborators and staff of the Canadian Breast Cancer Study, and project coordinators Agnes Lai and Celine Morissette.

Investigators from the CPS-II cohort thank the participants and Study Management Group for their invaluable contributions to this research. They also acknowledge the contribution to this study from central cancer registries supported through the Centers for Disease Control and Prevention National Program of Cancer Registries, as well as cancer registries supported by the National Cancer Institute Surveillance Epidemiology and End Results program.

The authors would like to thank the California Teachers Study Steering Committee that is responsible for the formation and maintenance of the Study within which this research was conducted. A full list of California Teachers Study (CTS) team members is available at https://www.calteachersstudy.org/team.

We thank the participants and the investigators of EPIC (European Prospective Investigation into Cancer and Nutrition).

ESTHER thanks Hartwig Ziegler, Sonja Wolf, Volker Hermann, Christa Stegmaier, Katja Butterbach.

PROCAS thank NIHR for funding.

The GENICA Network: Dr Margarete Fischer-Bosch-Institute of Clinical Pharmacology, Stuttgart, and University of Tübingen, Germany [HB, RH, Wing-Yee Lo], Department of Internal Medicine, Johanniter GmbH Bonn, Johanniter Krankenhaus, Bonn, Germany [Yon-Dschun Ko, Christian Baisch], Institute of Pathology, University of Bonn, Germany [Hans-Peter Fischer], Molecular Genetics of Breast Cancer, Deutsches Krebsforschungszentrum (DKFZ), Heidelberg, Germany [Ute Hamann], Institute for Prevention and Occupational Medicine of the German Social Accident Insurance, Institute of the Ruhr University Bochum (IPA), Bochum, Germany [Thomas Brüning, Beate Pesch, Sylvia Rabstein, Anne Lotz]; and Institute of Occupational Medicine and Maritime Medicine, University Medical Center Hamburg-Eppendorf, Germany [Volker Harth].

KARMA and SASBAC thank the Swedish Medical Research Counsel.

KBCP thanks Eija Myöhänen.

LAABC thanks all the study participants and the entire data collection team, especially Annie Fung and June Yashiki.

MARIE thanks Petra Seibold, Ursula Eilber and Muhabbet Celik.

MASTOS thanks all the study participants and express appreciation to the doctors: Yiola Marcou, Eleni Kakouri, Panayiotis Papadopoulos, Simon Malas and Maria Daniel, as well as to all the nurses and volunteers who provided valuable help towards the recruitment of the study participants.

The MCCS was made possible by the contribution of many people, including the original investigators, the teams that recruited the participants and continue working on follow-up, and the many thousands of Melbourne residents who continue to participate in the study.

The MISS study group acknowledges the former Principal Investigator, Professor Håkan Olsson.

© 2025 Le Cornet C et al. *JAMA Network Open*.

We thank the coordinators, the research staff and especially the MMHS participants for their continued collaboration on research studies in breast cancer.

NBHS and SBCGS thank study participants and research staff for their contributions and commitment to the studies.

For NHS and NHS2 the study protocol was approved by the institutional review boards of the Brigham and Women's Hospital and Harvard T.H. Chan School of Public Health, and those of participating registries as required. We would like to thank the participants and staff of the NHS and NHS2 for their valuable contributions as well as the following state cancer registries for their help: AL, AZ, AR, CA, CO, CT, DE, FL, GA, ID, IL, IN, IA, KY, LA, ME, MD, MA, MI, NE, NH, NJ, NY, NC, ND, OH, OK, OR, PA, RI, SC, TN, TX, VA, WA, WY. The authors assume full responsibility for analyses and interpretation of these data.

PBCS thanks Louise Brinton, Mark Sherman, Neonila Szeszenia-Dabrowska, Beata Peplonska, Witold Zatonski, Pei Chao, Michael Stagner.

© 2025 Le Cornet C et al. *JAMA Network Open*.
