# Supplementary material for: Exogenous Hormones, Tumor Intrinsic Subtypes, and Breast Cancer
Source: JAMA Netw Open. 2025 Jul 7;8(7):e2519236. doi: 10.1001/jamanetworkopen.2025.19236 (PMC12235495; doi:10.1001/jamanetworkopen.2025.19236)
Supplement: Supplement 2. — Data Sharing Statement [file jamanetwopen-e2519236-s002.pdf]

Data Sharing Statement

Le Cornet. Exogenous Hormones, Tumor Intrinsic Subtypes, and Breast Cancer. *JAMA Netw Open*. Published July 07, 2025. doi:10.1001/jamanetworkopen.2025.19236

Data
Data available: No

Additional Information
Explanation for why data not available: The data underlying this article cannot be shared publicly due to ethical guidelines, aiming to protect the privacy of individuals that participated in the study. The data may be shared on reasonable request to the corresponding author, after permission from the Institutional Review Board.
